# Supplementary material for: A vaccine targeting mutant IDH1 in newly diagnosed glioma
Source: Nature. 2021 Mar 24;592(7854):463–8. doi: 10.1038/s41586-021-03363-z (PMC8046668; doi:10.1038/s41586-021-03363-z)
Supplement: Supplementary file 1 — Nine supplementary tables including legends show demographic information, clinical, molecular, and genetic baseline characteristics on patients in the NOA16 trial across trial sites and a control cohort, give an overview on screening failures and AEs, and sum up primary endpoint and exploratory cellular immune response analyses. [file 41586_2021_3363_MOESM1_ESM.pdf]

nature portfolio

https://doi.org/10.1038/s41586-021-03363-z

Supplementary information

A vaccine targeting mutant IDH1 in newly diagnosed glioma

In the format provided by the authors and unedited

Supplementary Information to:

A vaccine targeting mutant IDH1 in newly diagnosed glioma

Supplementary Tables

Supplementary Table 1. NOA16 trial sites.

Trial sites (all in Germany)	Number of pts with completed screening	Number of pts enrolled	Number of pts treated (SDS)	Number of pts evaluable (IDS)
Heidelberg	20	14	14	14
Freiburg	3	2	2	2
Berlin	5	4	4	4
Essen	6	3	2	0
Dresden	3	3	3	3
Frankfurt	3	3	3	3
Tübingen	4	4	4	4
All centers	44	33	32	30

NOA16 was a multi-center trial at seven sites across Germany. In total, 44 patients were screened and 30 patients were evaluable for immunogenicity. Pts, patients; SDS, safety data set; IDS, immunogenicity data set.

Supplementary Table 2. Reasons for screening failure.

Screening ID	Reason due to molecular screening	Reason regarding Inclusion-/Exclusion criteria
01-001		Incl. criteria violated: Evidence of two informed consent documents personally signed and dated by the patient
		Incl. criteria violated: Patient is willing and able to comply with scheduled visits, treatment plan, laboratory tests, and other study procedures.
01-002	IDH1R132H absent	
01-006		Incl. criteria violated: Woman of child-bearing potential
		Excl. criteria met: Pregnancy or lactation
01-007	no Absence of chromosomal 1p/19q co-deletion, no loss of ATRX expression	
01-008	no Loss of ATRX expression	
01-014	no Absence of chromosomal 1p/19q co-deletion, no loss of ATRX expression	
02-002	IDH1R132H absent, no loss of ATRX expression	
03-003		Excl. criteria met: Abnormal (= Grade 2 CTCAE v4.0) laboratory values for hematology, liver and renal function (serum creatinine).
04-004		Excl. criteria met: Progressive (incl. PsPD) or recurrent disease after radiation therapy, chemotherapy or radiochemotherapy based on local MRI assessment
04-005		Incl. criteria violated: Evidence of two informed consent documents personally signed and dated by the patient
04-006		Excl. criteria met: Progressive (incl. PsPD) or recurrent disease after radiation therapy, chemotherapy or radiochemotherapy based on local MRI assessment

Screening failed due to either molecular parameters or violated inclusion / met exclusion criteria. First two digits of screening ID refer to trial center number. N = 11 screening failures.

Supplementary Table 3. Baseline characteristics and PsPD in NOA16 and control cohort of astrocytoma

pt ID	sex	Age (years)	WHO grade	localization	extent of resection ST: subtotal C: complete	SOC pre IDH1-vac Concomitant (c)TMZ: 75 mg/m^2 BSA daily during RT Monotherapy (mono-) TMZ: 3 cycles RT: 30 x 2 Gy, if not other specified	SOC during IDH1-vac Adjuvant (a)TMZ: 150-200 mg/m^2 BSA 5/28 days, 6 cycles if not other specified	methylation class low grade (L) high grade (H) n.d.: not determined
ID01	f	31	3	overlapping	ST	cTMZ + RT	aTMZ	L
ID02	m	40	4	frontal	C	cTMZ + RT	aTMZ	L
ID03	m	44	3	frontal	C	cTMZ + RT	aTMZ	L
ID04	f	40	3	frontal	biopsy	RT	no aTMZ	n.d.
ID05	m	47	3	temporal	ST	cTMZ + RT	aTMZ	H
ID06	f	46	4	frontal	ST	cTMZ + RT	aTMZ	H
ID07	m	37	4	frontal	C	cTMZ + RT	aTMZ	H
ID08	f	33	4	frontal	ST	cTMZ + RT	aTMZ	n.d.
ID09	f	43	4	frontal	ST	cTMZ + RT	aTMZ	H
ID10	f	54	3	frontal	biopsy	mono-TMZ	aTMZ	n.d.
ID11	m	39	3	frontal	ST	mono-TMZ	aTMZ (12 cycles)	n.d.
ID12	f	29	4	frontal	ST	cTMZ + RT	aTMZ	H
ID13	m	35	3	frontal	C	RT (59.4 Gy)	aTMZ	L
ID14	f	46	4	frontal	C	cTMZ + RT	aTMZ	H
ID15	f	26	3	other	ST	cTMZ + RT	no aTMZ	n.d.
ID17	f	46	3	frontal	C	mono-TMZ	aTMZ (12 cycles)	n.d.
ID18	m	49	3	other	C	cTMZ + RT	aTMZ	n.d.
ID19	m	43	3	frontal	C	RT (59.4 Gy)	aTMZ (10 cycles)	L
ID20	m	25	3	frontal	C	RT	aTMZ	L
ID21	m	35	3	temporal	ST	cTMZ + RT	aTMZ (8 cycles)	n.d.
ID22	f	35	4	frontal	C	cTMZ + RT	aTMZ	H
ID23	m	53	3	frontal	biopsy	cTMZ + RT (50.0 Gy)	aTMZ	H
ID24	m	46	3	frontal	C	cTMZ + RT	aTMZ	L
ID25	m	41	3	frontal	ST	RT	no aTMZ	L
ID26	m	34	4	frontal	ST	cTMZ + RT	aTMZ	L
ID27	m	42	3	frontal	C	cTMZ + RT (59.4 Gy)	aTMZ	L
ID28	m	40	3	temporal	C	cTMZ + RT (54.0 Gy)	aTMZ	H
ID29	m	24	4	temporal	C	cTMZ + RT	aTMZ	L
ID30	m	28	3	frontal	C	cTMZ + RT	aTMZ (3 cycles)	H
ID31	m	56	3	frontal	C	cTMZ + RT	aTMZ	L
ID32	m	53	4	other	ST	cTMZ + RT	aTMZ	L
ID33	f	54	3	frontal	C	RT (59.4 Gy)	aTMZ	L
control cohort								
pt ID	sex	age (years)	WHO grade	localization	extent of resection	Treatment	Methylation class	PsPD
HD01	m	26	3	temporal	biopsy	RT	L	no
HD02	m	27	3	frontal	C	mono-TMZ	L	no

HD03	f	34	3	frontal	C	mono-TMZ	L	no
HD04	f	31	3	frontal	ST	RT	L	no
HD05	m	23	3	temporal	biopsy	mono-TMZ	L	no
HD06	m	27	4	frontal	C	RT	n.d.	no
HD07	f	34	4	frontal	C	RT	L	yes
HD08	f	53	4	frontal	C	cTMZ + RT	L	yes
HD09	m	64	4	frontal	SC	cTMZ + RT	L	no
HD10	f	27	3	frontal	C	mono-TMZ	L	no
HD11	f	40	3	frontal	ST	cTMZ + RT + aTMZ	L	yes
HD12	f	24	4	frontal	C	cTMZ + RT + aTMZ	L	no
HD13	m	33	3	frontal	ST	cTMZ + RT + aTMZ	n.d.	no
HD14	m	31	4	frontal	C	cTMZ + RT + aTMZ	n.d.	no
HD15	f	45	3	other	C	cTMZ + RT + aTMZ	L	no
HD16	m	41	3	frontal	C	cTMZ + RT + aTMZ	n.d.	no
HD17	m	32	3	other	C	cTMZ + RT + aTMZ	L	yes
HD18	f	27	3	frontal	ST	cTMZ + RT + aTMZ	L	no
HD19	f	30	3	frontal	biopsy	cTMZ + RT + aTMZ	L	no
HD20	m	32			ST	cTMZ + RT + aTMZ	L	no
HD21	m	36	3	temporal	biopsy	cTMZ + RT + aTMZ	L	no
HD22	m	41	4	frontal	ST	cTMZ + RT + aTMZ	L	no
HD23	m	21	3	frontal	C	cTMZ + RT + aTMZ	L	no
HD24	f	20	3	frontal	C	cTMZ + RT + aTMZ	L	yes
HD25	m	56	3	frontal	ST	cTMZ + RT + aTMZ	L	no
HD26	m	40	4	frontal	ST	cTMZ + RT + aTMZ	L	no
HD27	f	32	3	frontal	C	cTMZ + RT + aTMZ	L	no
HD28	m	34	3	temporal	C	cTMZ + RT + aTMZ	n.d.	no
HD29	f	27	4	temporal	C	cTMZ + RT + aTMZ	L	no
HD30	m	52	3	frontal	C	cTMZ + RT	L	no
HD31	m	33	3	frontal	C	mono-TMZ	L	no
HD32	m	31	4	other	ST	cTMZ + RT + aTMZ	L	yes
HD33	m	35	3	frontal	C	cTMZ + RT + aTMZ	L	yes
HD34	m	56	3	temporal	C	cTMZ + RT + aTMZ	L	no
HD35	f	22	3	temporal	C	cTMZ + RT + aTMZ	L	no
HD36	m	44	3	frontal	C	cTMZ + RT + aTMZ	n.d.	no
HD37	m	41	3	overlapping	C	cTMZ + RT + aTMZ	L	no
HD38	m	47	3	frontal	ST	cTMZ + RT + aTMZ	L	no
HD39	m	43	3	frontal	ST	cTMZ + RT + aTMZ	L	yes
HD40	f	44	4	frontal	C	cTMZ + RT + aTMZ	H	no
HD41	m	56	4	temporal	C	cTMZ + RT + aTMZ	H	no
HD42	f	51	4	overlapping	C	cTMZ + RT + aTMZ	H	no
HD43	m	52	4	frontal	ST	cTMZ + RT + aTMZ	H	no
HD44	m	48	4	frontal	C	cTMZ + RT + aTMZ	H	no
HD45	f	36	4	frontal	C	cTMZ + RT + aTMZ	H	yes
HD46	f	34	4	frontal	C	cTMZ + RT + aTMZ	H	no
HD47	m	61	4	temporal	ST	mono-TMZ	H	no
HD48	f	63	4	frontal	C	cTMZ + RT + aTMZ	H	no
HD49	m	51	4	frontal	C	cTMZ + RT + aTMZ	H	no
HD50	m	43	4	temporal	ST	RT	H	no
HD51	m	27	4	temporal	C	cTMZ + RT + aTMZ	H	no
HD52	m	29	4	frontal	C	RT	H	no
HD53	f	33	4	temporal	ST	cTMZ + RT + aTMZ	n.d.	no
HD54	f	45	4	overlapping	ST	cTMZ + RT + aTMZ	H	no
HD55	f	37	4	overlapping	C	cTMZ + RT + aTMZ	H	yes
HD56	f	29	4	frontal	C	cTMZ + RT + aTMZ	H	no
HD57	m	33	4	frontal	C	cTMZ + RT + aTMZ	n.d.	no
HD58	m	48	4	frontal	C	cTMZ + RT + aTMZ	H	no

HD59	f	45	4	frontal	ST	cTMZ + RT + aTMZ	H	no
HD60	f	50	4	frontal	C	cTMZ + RT + aTMZ	H	no

All patients of the control cohort treated in Heidelberg, median observation time, 7.3 years. Pt, patient; m, male; f, female; ST, subtotal resection; C, complete resection; RT, radiotherapy, 30 x 2 Gy, if not specified differently; TMZ, temozolomide; (c)TMZ, concomitant TMZ, 75mg/m2 body surface area (BSA) daily during RT; (a)TMZ, adjuvant TMZ, 150-200 mg/m2 BSA 5/28 days, 6 cycles if not specified differently; mono-TMZ, monotherapy with TMZ, 3 cycles; L, methylation class low grade; H, methylation class high grade; n.d., not determined. N(control cohort) = 60 patients; N(NOA16 cohort) = 32 patients.

Supplementary Table 4. Treatment emergent AEs of the SDS.

**Safety Data Set (N=32)**<br>**Patient-days of total IDH1-vac exposure: 4834 days**	**AEs**<br>**All causalities n (%)**	**AEs**<br>**Treatment-related n (%)**
Patients with AE	32 (100)	29 (90.6)
Patients with SAEs	2 (6.3)	1 (3.1)
Patients with severe AEs (CTCAE grade 3/4)	5 (15.6)	-
Patients discontinued due to AE	1 (3.1)	-
Patients with dose reduction or temporary discontinuation due to AE	3 (9.4)	1 (3.1)
**AEs (all causalities) by Preferred Term in > 5 % of patients (MedDRA 20.1)**	**Frequencies n (%, 95%-CI)**	
Injection site induration	21 (65.6, 95%-CI: 46.81-81.43)	
Injection site erythema	15 (46.9, 95%-CI: 29.09-65.26)	
Erythema	5 (15.6, 95%-CI: 5.28-32.79)	
Influenza like illness	3 (9.4, 95%-CI: 1.98-25.02)	
Injection site pruritus	3 (9.4, 95%-CI: 1.98-25.02)	
Injection site reaction	2 (6.3, 95%-CI: 0.77-20.81)	

AE, adverse event; SAE, serious AE; CTCAE, NCI Common Terminology Criteria for Adverse Events.

Supplementary Table 5. All adverse events according to System Organ Class.

System Organ Class	No. of pts (%) with AE all causalities	No. of pts (%) with AE related to IDH1-vac
General disorders and administration site conditions	29 (90.6)	27 (84.4)
Gastrointestinal disorders	16 (50.0)	-
Infections and infestations	11 (34.4)	1 (3.1)
Nervous system disorders	10 (31.3)	1 (3.1)
Investigations	8 (25.0)	1 (3.1)
Skin and subcutaneous tissue disorders	8 (25.0)	8 (25.0)
Musculoskeletal and connective tissue disorders	7 (21.9)	1 (1.3)
Blood and lymphatic system disorders	3 (9.4)	-
Eye disorders	2 (6.3)	-
Psychiatric disorders	2 (6.3)	1 (3.1)
Respiratory, thoracic and mediastinal disorders	2 (6.3)	-
Ear and labyrinth disorders	1 (3.1)	-
Endocrine disorders	1 (3.1)	-
Immune system disorders	1 (3.1)	1 (3.1)
Injury, poisoning and procedural complications	1 (3.1)	-
Metabolism and nutrition disorders	1 (3.1)	-
Neoplasms benign, malignant and unspecified (incl cycts and polyps)	1 (3.1)	-
Vascular disorders	1 (3.1)	-

System Organ Class according to MedDRA 20.1. Adverse events in the safety data set (SDS) are shown and classified as treatment-related or with all causalities. AE, adverse event; pts, patients; IDH1-vac, IDH1R132H vaccine. N = 32 patients in the SDS.

Supplementary Table 6. HLA types of the Safety Data Set

ID	HLA-A 1	HLA-A 2	HLA-B 1	HLA-B 2	HLA-C 1	HLA-C 2	HLA-DRB 1	HLA-DRB 2	HLA-DQB 1	HLA-DQB 2	HLA-DPB 1	HLA-DPB 2	DRB3 1	DRB3 2	DRB4 1	DRB4 2	DRB5 1	DRB5 2	HLA-DQA1 1	HLA-DQA1 2	HLA-DPA1 1	HLA-DPA1 2
1	24:02:0	03:01:0	35:02:0	47:01:0	04:01:0	06:02:0	11:04:0	13:01:0	06:03:0	03:01:0	02:01	04:01:0	01:BZF	02:ERV	NNNN	NNNN	NNNN	NNNN	01:03:0	05:BZC	01:CES	01:CES
2	02:01:0	26:01:0	38:01:0	15:01:0	12:03:0	03:03:0	14:01:0	01:03:0	05:AW	03:01:0	04:FNV	04:HJM	02:24	NNNN	NNNN	NNNN	NNNN	NNNN	01:CAV	05:BZC	01:CES	01:CES
3	24:02:0	25:01:0	18:01:0	18:01:0	07:01:0	12:03:0	01:03	01:01:0	05:01:0	05:01:0	04:01:0	02:01	NNNN	NNNN	NNNN	NNNN	NNNN	NNNN	01:01:0	01:01:0	01:03:0	01:03:0
4	01:01:0	02:01:0	40:01:0	50:01:0	05:01:0	03:04:0	13:02:0	07:01:0	06:04:0	02:02:0	03:FYK	04:ADC	03:GM	NNNN	01:03:0	NNNN	NNNN	NNNN	01:CAV	02:01:0	01:CES	01:CES
5	29:02:0	25:01:0	18:01:0	44:03:0	16:AUE	12:AUE	04:01:0	07:01:0	03:211	02:02:0	01:AET	04:01:0	NNNN	NNNN	01:01:0	01:03:0	NNNN	NNNN	02:01:0	03:01:0	01:CES	02:RYH
6	03:01:0	03:01:0	07:02:0	44:02:0	07:02:0	07:04:0	14:54:0	16:01:0	05:02:0	05:03:0	04:01:0	02:01	02:03	NNNN	NNNN	NNNN	02:XA	NNNN	01:02	01:CAV	01:CES	01:CES
7	02:01:0	11:01:0	40:01:0	35:01:0	04:01:0	03:04:0	15:01:0	01:01:0	06:02:0	05:01:0	04:FNV	04:HJM	NNNN	NNNN	NNNN	NNNN	01:HUJ	NNNN	01:CAV	01:CAV	01:CES	01:CES
8	02:01:0	32:01:0	51:01:0	44:02:0	05:01:0	15:02:0	04:02:0	13:01:0	06:03:0	03:02:0	04:01:0	04:01:0	02:ERV	NNNN	01:03:0	NNNN	NNNN	NNNN	01:03:0	03:01:0	01:CES	01:CES
9	23:01:0	01:01:0	57:01:0	41:02:0	17:01:0	06:02:0	11:04:0	07:01:0	03:03:0	03:01:0	13:AKX	13:AKX	02:ERV	NNNN	01:03:0	NNNN	NNNN	NNNN	02:01:0	05:BZC	02:CJA	02:CJA
10	01:01:0	03:01:0	07:02:0	08:01:0	07:01:0	07:02:0	04:07:0	03:01:0	02:01:0	03:01:0	03:FYK	04:ADC	01:BZF	NNNN	01:03:0	NNNN	NNNN	NNNN	03:CEY	05:01	01:CES	01:CES
11	30:02:0	24:02:0	18:01:0	35:03:0	05:01:0	04:01:0	03:01:0	13:02:0	06:04:0	02:01:0	04:HJM	04:FNV	02:ERV	03:GM	NNNN	NNNN	NNNN	NNNN	01:CAV	05:01	01:CES	01:CES
12	02:01:0	02:01:0	51:01:0	15:01:0	14:02:0	04:01:0	08:01:0	08:01:0	04:02:0	04:02:0	05:RGP	03:FNV	NNNN	NNNN	NNNN	NNNN	NNNN	NNNN	04:01:0	04:02	01:CES	02:RYH
13	30:01:0	02:01:0	57:01:0	13:02:0	06:02:0	06:02:0	07:01:0	07:01:0	03:03:0	02:02:0	03:FYK	02:AHZ	NNNN	NNNN	01:03:0	01:03:0	NNNN	NNNN	02:01:0	02:01:0	01:CES	01:CES
14	02:05:0	23:01:0	49:01:0	50:01:0	06:02:0	07:01:0	04:05:0	07:01:0	03:02:0	02:02:0	03:FYK	04:ADC	NNNN	NNNN	01:03:0	01:03:0	NNNN	NNNN	02:01:0	03:01:0	01:CES	01:CES

15	02:01:0	02:01:0	07:02:0	44:02:0	05:01:0	07:02:0	15:01:0	15:01:0	06:03:0	06:02:0	04:01:0	04:01:0	NNNN	NNNN	NNNN	NNNN	01:HUJ	01:HUJ	01:CAV	01:CAV	01:CES	01:CES
17	01:01:0	03:01:0	07:02:0	08:01:0	07:AM	07:AUD	15:01:0	03:01:0	06:02:0	02:01:0	03:FYK	04:ADC	01:BZF	NNNN	NNNN	NNNN	01:HUJ	NNNN	01:CAV	05:01	01:CES	02:01:0
18	01:01:0	02:01:0	07:04	08:01:0	07:AM	07:AUD	03:01:0	15:01:0	06:02:0	02:01:0	01:AET	04:01:0	01:BZF	NNNN	NNNN	NNNN	01:HUJ	NNNN	01:CAV	05:01	01:CES	02:01:0
19	02:01:0	66:01:0	40:01:0	41:02:0	03:04:0	17:01:0	13:03:0	13:02:0	06:04:0	03:01:0	03:FYK	02:AHZ	01:BZF	03:GM	NNNN	NNNN	NNNN	NNNN	01:CAV	05:BZC	01:CES	01:CES
20	32:01:0	26:01:0	57:01:0	44:02:0	05:01:0	06:02:0	12:JV	07:01:0	03:03:0	03:01:0	04:HJM	04:FNV	02:ERV	NNNN	01:03:0	NNNN	NNNN	NNNN	02:01:0	05:BZC	01:CES	01:CES
21	03:01:0	03:01:0	07:02:0	08:01:0	07:AM	07:AUD	03:01:0	01:01:0	05:01:0	02:01:0	04:01:0	04:01:0	01:BZF	NNNN	NNNN	NNNN	NNNN	NNNN	01:CAV	05:01	01:CES	01:CES
22	02:01:0	03:01:0	49:01:0	15:01:0	01:02:0	07:01:0	04:05:0	13:01:0	06:03:0	03:02:0	13:AKX	05:RGP	02:ERV	NNNN	01:03:0	NNNN	NNNN	NNNN	01:03:0	03:CEY	02:01:0	02:RYH
23	25:01:0	25:01:0	55:01:0	44:02:0	03:03:0	07:04:0	14:54:0	11:01:0	05:03:0	03:01:0	03:FYK	04:ADC	02:02:0	02:02:0	NNNN	NNNN	NNNN	NNNN	01:01:0	05:01:0	01:03:0	01:03:0
24	03:01:0	29:02:0	35:01:0	44:03:0	16:01:0	04:01:0	01:01:0	07:01:0	05:01:0	02:02:0	04:01:0	04:01:0	NNNN	NNNN	01:01:0	NNNN	NNNN	NNNN	01:CAV	02:01:0	01:CES	01:CES
25	01:01:0	11:01:0	52:01:0	08:01:0	12:02:0	07:01:0	15:02:0	15:01:0	06:02:0	06:01	14:01:0	02:01	NNNN	NNNN	NNNN	NNNN	01:02	01:HUJ	01:03:0	01:CAV	01:CES	02:CJA
26	01:01:0	01:01:0	52:01:0	37:01:0	12:02:0	06:02:0	16:01:0	10:01:0	05:01:0	05:02:0	03:FYK	04:ADC	NNNN	NNNN	NNNN	NNNN	02:XA	NNNN	01:02	01:CAV	01:CES	01:CES
27	24:02:0	03:01:0	07:02:0	44:02:0	05:01:0	07:02:0	11:01:0	13:01:0	06:03:0	03:01:0	13:01:0	02:AHZ	01:BZF	02:ERV	NNNN	NNNN	NNNN	NNNN	01:03:0	05:CG	01:CES	02:01:0
28	02:01:0	11:01:0	35:03:0	18:01:0	05:01:0	12:03:0	14:54:0	01:01:0	05:01:0	05:03:0	02:01	02:01	02:ERV	NNNN	NNNN	NNNN	NNNN	NNNN	01:CAV	01:CAV	01:CES	01:CES
29	02:01:0	02:01:0	15:01:0	07:02:0	03:04:0	07:02:0	04:01:0	04:01:0	03:02:0	03:02:0	15:01:0	04:01:0	NNNN	NNNN	01:03:0	01:03:0	NNNN	NNNN	03:01:0	03:01:0	01:04	01:CES
30	23:01:0	23:01:0	49:01:0	44:03:0	07:01:0	04:01:0	11:01:0	07:01:0	03:01:0	02:02:0	04:01:0	02:01	02:ERV	NNNN	01:01:0	NNNN	NNNN	NNNN	02:01:0	05:BZC	01:CES	01:CES
31	31:01:0	24:02:0	35:01:0	44:02:0	05:01:0	01:02:0	14:54:0	11:04:0	05:03:0	03:01:0	02:01	04:01:0	02:ERV	02:ERV	NNNN	NNNN	NNNN	NNNN	01:CAV	05:BZC	01:CES	01:CES

32	01:01:0	26:01:0	49:01:0	55:01:0	03:03:0	12:03:0	11:01:0	07:01:0	02:02:0	03:01:0	11:01:0	04:01:0	02:ERV	NNNN	01:01:0	NNNN	NNNN	NNNN	02:01:0	05:BZC	01:CES	02:CJA
33	01:01:0	02:01:0	15:01:0	07:02:0	03:03:0	07:02:0	04:01:0	07:01:0	03:02:0	02:02:0	10:01:0	04:02:0	NNNN	NNNN	01:01:0	01:03:0	NNNN	NNNN	02:01:0	03:01:0	01:CES	02:CJA

HLA types of 16 alleles and 6 paralogues are shown. NNNN, not detected. For decoding multiple allele codes (MAC) please visit : https://hml.nmdp.org/MacUI/. N = 32 patients in the SDS.

Supplementary Table 7. Mutation-specificity scores in the Immunogenicity Data Set

ID	MSS	ID	MSS
ID01	87.78	ID17	43.78
ID02	9.00	ID18	25.78
ID03	110.11	ID20	82.78
ID04	64.89	ID22	113.67
ID05	10.44	ID23	89.44
ID06	231.44	ID24	41.11
ID07	33.00	ID25	113.22
ID08	36.56	ID26	39.88
ID09	42.56	ID27	130.33
ID10	45.33	ID28	95.89
ID11	115.76	ID29	21.00
ID12	146.56	ID30	12.00
ID13	24.11	ID31	77.11
ID14	21.00	ID32	62.44
ID15	55.33	ID33	108.89

Mutation-specificity scores were calculated from longitudinal ELISpot data of T cell responses to mutated and wildtype IDH1 peptide as depicted in ED Figure 7. MSS, mutation-specificity score. N = 30 patients in the IDS.

Supplementary Table 8. Exploratory analyses performed within the Safety Data Set.

ID	PLA^{primary}	PLA^{relapse}	TCRB^{PsPD}	(scRNA-TCR-seq)^{PsPD}	longitudinal TCRB^{PBMC}	(RNA-panel + 850k methylation)^{primary}	(RNA-panel + 850k methylation)^{relapse}	HLA typing	Flow cytometry^{PBMC}
01	x					x		x	x
02						x		x	x
03					x	x		x	x
04					x			x	x
05	x				x	x		x	x
06					x	x		x	x
07	x					x		x	x
08			x	x	x			x	x
09		x			x	x		x	x
10	x				x			x	x
11					x			x	x
12	x				x	x		x	x
13					x	x		x	x
14	x					x		x	x
15					x			x	x
17					x			x	x
18								x	x
19						x		x	
20	x				x	x		x	x
21					x			x	
22	x				x	x		x	x
23					x	x		x	x
24					x	x		x	x
25	x	x			x	x	x	x	x
26	x				x	x		x	x
27					x	x		x	x
28	x				x	x		x	x
29						x		x	x
30					x	x		x	x
31	x				x	x		x	x
32	x				x	x		x	x
33					x	x		x	x
	N=13	N=2	N=1	N=1	N=25	N=24	N=1	N=32	N=30

Cellular and molecular analyses were performed on tumor tissues, peripheral blood and peripheral blood-derived cells at indicated time points during the study. PLA, proximity ligation assay on FFPE tissues; TCRB, TCR beta deep sequencing on cryopreserved tissues or PBMC; ScRNA-TCR-seq, single cell TCR- and single cell RNA-sequencing on slow-frozen T cells; RNA panel, targeted RNA panel sequencing on FFPE tissue; HLA typing, human leukocyte antigen next-generation sequencing; primary, primary tissue; relapse, post-treatment relapse tissue; PsPD, pseudoprogression. N = 32 patients in the SDS.

Supplementary Table 9. Molecular analyses of the Safety Data Set

ID	CDKN2A/B deletions	tsne cluster methylation	CNV-L
ID01	0	L	L
ID02	0	L	L
ID03	het	L	L
ID04	n.d.	n.d.	n.d.
ID05	het*	H	H
ID06	het*	H	H
ID07	het	H	H
ID08	n.d.	n.d.	n.d.
ID09	homo	H	H
ID10	n.d.	n.d.	n.d.
ID11	n.d.	n.d.	n.d.
ID12	homo	H	H
ID13	0	L	L
ID14	0	H	H
ID15	n.d.	n.d.	n.d.
ID17	n.d.	n.d.	n.d.
ID18	n.d.	n.d.	n.d.
ID19	het*	L	L
ID20	0	L	L
ID21	n.d.	n.d.	n.d.
ID22	homo	H	H
ID23	het	H	L
ID24	0	L	L
ID25	0 (P) het (R)	L (P+R)	L (P+R)
ID26	0	L	L
ID27	0	L	L
ID28	0	H	L
ID29	0	L	L
ID30	homo	H	H
ID31	0	L	L
ID32	0	L	L
ID33	0	L	L

CDKN2A/B status, methylation, and copy number variations are listed. CDKN, cyclin-dependent kinase inhibitor; 0, no CDKN2A/B deletion; homo, homozygous CDKN2A/B deletions; het, heterozygous CDKN2A/B deletion; *, heterozygous CDKN2A/B deletion ambiguous; H (tsne), methylation class high grade astrocytomas; L (tsne), methylation class low grade astrocytomas 31; CNV-L, copy number variation load; H (CNV-L), high CNV-L (>350 Mb); L (CNV-L), low CNV-L (< 350 Mb); P, primary tissue; R, relapse tissue; n.d. not determined. N = 32 patients in the SDS.
