# Supplementary material for: A vaccine targeting mutant IDH1 in newly diagnosed glioma
Source: Nature. 2021 Mar 24;592(7854):463–8. doi: 10.1038/s41586-021-03363-z (PMC8046668; doi:10.1038/s41586-021-03363-z)
Supplement: Supplementary file 3 — This file contains the clinical trial protocol [file 41586_2021_3363_MOESM3_ESM.pdf]

NATIONALES CENTRUM
FÜR TUMORERKRANKUNGEN
HEIDELBERG

getragen von:
Deutsches Krebsforschungszentrum
Universitätsklinikum Heidelberg
Thoraxklinik-Heidelberg
Deutsche Krebshilfe

Clinical Trial Protocol

Targeting IDH1R132H in WHO grade III-IV IDH1R132H-mutated gliomas by a peptide vaccine – a Phase I safety, tolerability and immunogenicity multicenter trial

(NOA-16)

Clinical Trial Code: NCT-2013-0216

EudraCT No.: 2014-000503-27

Clinical Phase: Phase I, first-in-man
Version and date: Final version 2.1, 31.03.2015

Sponsor: Ruprecht-Karls-University Heidelberg, Medical Faculty
- represented in law by Universitätsklinikum Heidelberg and its Commercial Director Ms. Irmtraut Gürkan -
Im Neuenheimer Feld 672
69120 Heidelberg, Germany

Phone: +49 (0)6221 / 56-7002
Fax: +49 (0)6221 / 56-4888
E-Mail: Irmtraut.Guerkan@med.uni-heidelberg.de

CONFIDENTIAL: This protocol contains confidential information and is intended solely for the guidance of the clinical investigation. This protocol may not be disclosed to parties not associated with the clinical investigation or used for any purpose without the prior written consent of the Principal Investigator/ Coordinating Investigator.

Responsibilities

Coordinating Investigator (Leiter der klinischen Prüfung *)

Prof. Dr. Michael Platten

Neurology Clinic
University Hospital Heidelberg
Neurooncology Program at the NCT

Im Neuenheimer Feld 400
69120 Heidelberg, Germany

Phone: +49 (0)6221 / 56-6804
Fax: +49 (0)6221 / 56-7554
E-Mail: michael.platten@med.uni-heidelberg.de

* according to § 40 German Drug Law (AMG)

Sponsor

Ruprecht-Karls-University Heidelberg, Medical Faculty
- represented in law by Universitätsklinikum Heidelberg and its Commercial Director Irmtraut Gürkan -

Im Neuenheimer Feld 672
69120 Heidelberg, Germany

Phone: +49 (0)6221 / 56-7002
Fax: +49 (0)6221 / 56-4888
E-Mail: Irmtraut.Guerkan@med.uni-heidelberg.de

Co-Investigator

Prof. Dr. Wolfgang Wick

Neurology Clinic
University Hospital Heidelberg
Neurooncology Program at the NCT

Im Neuenheimer Feld 400
69120 Heidelberg, Germany

Phone: +49 (0)6221 / 56-7075
Fax: +49 (0)6221 / 56-7554
E-Mail: wolfgang.wick@med.uni-heidelberg.de

Biometrics

Dipl.-Volksw. Irini Karapanagiotou-Schenkel

NCT Trial Center
National Center for Tumor Diseases (NCT)

Im Neuenheimer Feld 350
69120 Heidelberg, Germany

Phone: +49 (0)6221 / 56-6057
Fax: +49 (0)6221 / 56-5863
E-Mail: irini.schenkel@nct-heidelberg.de

Trial Coordination

Dr. Daniela Schilling

NCT Trial Center
National Center for Tumor Diseases (NCT)

Im Neuenheimer Feld 350
69120 Heidelberg, Germany

Phone: +49 (0)6221 / 56-6553
Fax: +49 (0)6221 / 56-5863
E-Mail: daniela.schilling@nct-heidelberg.de

Data Management

Marlene Diewald

NCT Trial Center
National Center for Tumor Diseases (NCT)

Im Neuenheimer Feld 350
69120 Heidelberg, Germany

Phone: +49 (0)6221 / 56-6017
Fax: +49 (0)6221 / 56-5861
E-Mail: marlene.diewald@nct-heidelberg.de

Clinical monitoring

KKS Heidelberg

University Hospital Heidelberg

Voßstraße 2
69115 Heidelberg, Germany

Phone: +49 (0)6221 / 56-34502
Fax: +49 (0)6221 / 56-33508

Pharmacovigilance

KKS Heidelberg

University Hospital Heidelberg

Voßstraße 2
69115 Heidelberg, Germany

Phone: +49 (0)6221 / 56-34507
Fax: +49 (0)6221 / 56-33725
E-Mail: kks_sae@med.uni-heidelberg

GMP Core Facility

Prof. Dr. Michael Schmitt

GMP Core Facility
Innere Medizin V
University Hospital Heidelberg
Im Neuenheimer Feld 410
69120 Heidelberg, Germany

Phone: +49 (0)6221 / 56-6614
Fax: +49 (0)6221 / 56-5740
E-Mail:
Michael.Schmitt@med.uni-heidelberg.de

Central Immune Laboratory

Dr. Theresa Schumacher

Neurology Clinic and
CCU Brain Tumor Immunology
University Hospital Heidelberg
Im Neuenheimer Feld 280
69120 Heidelberg, Germany

Phone: +49 (0)6221 / 42-3843
Fax: +49 (0)6221 / 42-3849
E-Mail: t.schumacher@dkfz.de

Central Neuropathology Laboratory

Prof. Dr. Andreas von Deimling

Department of Neuropathology
University Hospital Heidelberg
Im Neuenheimer Feld 224
69120 Heidelberg, Germany

Phone: +49 (0)6221 / 56-4650
Fax: +49 (0)6221 / 56-4566
E-Mail: andreas.vondeimling@med.
uni-heidelberg.de

Central Neuroradiology

Prof. Dr. Martin Bendszus

Department of Neuroradiology
University Hospital Heidelberg
Im Neuenheimer Feld 400
69120 Heidelberg, Germany

Phone: +49 (0)6221 / 56-7566
Fax: +49 (0)6221 / 56-4673
E-Mail: martin_bendszus@med.uni-
heidelberg.de

Summary

The study „Targeting IDH1R132H in WHO grade III-IV IDH1R132H-mutated gliomas by a peptide vaccine – a Phase I safety, tolerability and immunogenicity multicenter trial" (NOA-16) is a non-controlled, open-label, single arm, multicenter first-in-man phase I trial involving patients with gliomas carrying the IDH1R132H mutation.

The trial NOA-16 is a study of the German Cancer Consortium (DKTK). This trial will be performed within the Neurooncology Program, Neurology Clinic and NCT at the University Hospital Heidelberg and presumably 7 other centers in Germany, which are part of the DKTK. The trial is supported by the Neurooncology Working Group of the German Cancer Society (NOA) and will potentially include other NOA sites if resources suffice.

Within this trial, the IDH1 peptide vaccine – a 20mer peptide encompassing the IDH1R132H-mutated region emulsified in Montanide® – will be administered to 39 patients.

The patient population will be molecularly defined and include IDH1R132H mutant grade III and IV gliomas without co-deletion of 1p/19q and with loss of alpha-thalassemia/mental retardation syndrome X-linked (ATRX) expression. Patients must have received radiotherapy alone (treatment group 1), 3 cycles chemotherapy with temozolomide (TMZ; treatment group 2) or combined radiochemotherapy with TMZ (treatment group 3) prior to enrollment.

The IDH1 peptide vaccine will contain the IDH1R132H peptide emulsified in Montanide® and will be administered subcutaneously in combination with topical imiquimod (Aldara®). The vaccine will be administered in weeks 1, 3, 5, 7, 11, 15, 19 and 23 (visits 3-10). In treatment group 1 vaccination treatment will be done alone starting 4-6 weeks post radiotherapy. In treatment groups 2 and 3 vaccination treatment will be done in parallel with TMZ chemotherapy starting at day 10 of the 4th TMZ cycle (treatment group 2) or at day 10 of the 1st TMZ cycle post concomitant radiochemotherapy (treatment group 3). To be able to assess safety, tolerability and immunogenicity of the peptide vaccine 30 evaluable patients (39 patients in total) will be enrolled.

Diffuse and anaplastic gliomas are intrinsic malignant tumors of the central nervous system affecting 3/100,000 adults per year. Despite modern therapeutic approaches involving operation, radiotherapy and chemotherapy with TMZ these tumors are incurable with a median overall survival of 4-7 years. This poor outcome is mainly due to inherent malignant progression and secondary resistance to genotoxic therapies. A principle characteristic largely contributing to this aggressive biology is the highly invasive behavior, which allows the active evasion of glioma cells from the main tumor mass not only into the surrounding but also into distant normal brain tissue.

Effective therapeutic measures need to take into account both, distant tumor spread and secondary resistance to genotoxic therapies. Remarkably, these tumors may remain stable without clinical impairment of the affected patients for many years. Recurrence and malignant transformation, however, are inevitable. To date, there are no effective measures preventing recurrence and/or malignant transformation. Achieving an effective prevention by stimulating the immune system to control transformed tumor cells could result in long-term remission of the disease.

In 70-80% of diffuse and anaplastic gliomas mutations in the IDH1 gene occur. In the vast majority (> 90%) IDH1 mutations affect the catalytic site of the protein resulting in an amino acid exchange (Arg to His) at position 132 of the protein, hence the nomenclature IDH1R132H. IDH1R132H is the earliest mutation in diffuse and anaplastic gliomas rendering all tumor cells even during malignant progression positive for IDH1R132H. Hence, this mutation is a unique characteristic of these gliomas.

From an immunological point of view IDH1R132H represents an attractive tumor antigen specifically expressed in tumor but not normal cells. Patients with IDH1R132H-mutated gliomas may harbor mutation-specific T cells and antibodies, indicating that IDH1R132H is specifically

presented to and recognized by the immune system in a mutation-specific manner. Vaccination of humanized mice with the IDH1 peptide vaccine results in an anti-tumor immune response effective in controlling IDH1R132H-expressing tumors in a preventive and a therapeutic manner without causing toxicity.

The aim of this phase I trial is to evaluate the safety and tolerability of and immune response to the IDH1 peptide vaccine in patients with IDH1R132H-mutated gliomas without 1p/19q co-deletion and with loss of ATRX.

Zusammenfassung

Die klinische Prüfung „Gezielter Angriff der IDH1R132H-Mutation in Grad III-IV Gliomen mit einem Peptid-Impfstoff – eine Phase I-Studie zur Analyse der Sicherheit, Verträglichkeit und Immunogenität" (NOA-16) ist eine nicht-kontrollierte, offene, einarmige, multizentrische *first-in-man* Phase I-Studie an Patienten mit IDH1R132H-mutierten Gliomen.

Diese klinische Prüfung ist eine Studie des Deutschen Konsortiums für Translationale Krebsforschung (DKTK). Sie wird im Rahmen des Neuroonkologie-Programms in der Neurologischen Klinik und am NCT des Universitätsklinikums Heidelberg und voraussichtlich 7 weiteren Zentren in Deutschland durchgeführt, die Teil des DKTK sind. Die Studie wird durch die Neuroonkologische Arbeitsgemeinschaft der Deutschen Krebsgesellschaft (NOA) unterstützt und abhängig von den Ressourcen auch in weiteren NOA-Zentren geöffnet werden.

In dieser Studie wird 39 Patienten die IDH1-Peptidvakzine verabreicht – ein 20mer Peptid, das die IDH1-Region mit der R132H-Mutation umfasst und in Montanid® emulgiert ist.

Die Patientenpopulation ist molekular definiert durch IDH1R132H-mutierte Gliome des Grades III und IV ohne 1p/19q-Codeletion und mit Verlust der ATRX (*alpha-thalassemia/mental retardation syndrome X-linked*)-Expression. Vor Einschluss in die Studie haben die Patienten bereits eine Radiotherapie (Behandlungsgruppe 1), 3 Zyklen einer Temozolomid (TMZ)-Chemotherapie (Behandlungsgruppe 2) oder eine kombinierte TMZ-Radiochemotherapie (Behandlungsgruppe 3) erhalten.

Die IDH1-Peptidvakzine enthält das in Montanid® emulgierte IDH1R132H-Peptid und wird in Kombination mit topisch appliziertem Imiquimod (Aldara®) subkutan verabreicht. Eine Vakzinierung erfolgt in den Wochen 1, 3, 5, 7, 11, 15, 19 und 23. In Behandlungsgruppe 1 beginnt die Vakzinierung 4 – 6 Wochen nach abgeschlossener Radiotherapie. In den Behandlungsgruppen 2 bzw. 3 erfolgt die Vakzinierung parallel zur TMZ-Chemotherapie beginnend am Tag 10 des 4. TMZ-Zyklus (Behandlungsgruppe 2) bzw. am Tag 10 des 1. TMZ-Zyklus nach konkomitanter Radiochemotherapie (Behandlungsgruppe 3). Um die Sicherheit, Verträglichkeit und Immunogenität der IDH1-Peptidvakzine beurteilen zu können, sollen 30 auswertbare Patienten (39 Patienten gesamt) eingeschlossen werden.

Diffuse und anaplastische Gliome sind intrinsische maligne Tumore des zentralen Nervensystems und werden bei etwa 3/100.000 Erwachsenen pro Jahr diagnostiziert. Trotz moderner therapeutischer Verfahren wie Operation, Radiotherapie und Chemotherapie mit TMZ sind diese Tumoren nicht kurabel. Die mediane Gesamtüberlebenszeit der Patienten beträgt 4 - 7 Jahre. Diese schlechte Prognose ist hauptsächlich durch maligne Progression und Resistenzentwicklung gegenüber genotoxischen Therapien bedingt. Zu dieser aggressiven Biologie trägt das stark invasive Verhalten der Gliomzellen bei, durch das diese in das umliegende und weiter entfernte gesunde Hirngewebe eindringen können.

Effektive Therapien sollten beide Effekte kontrollieren: die Streuung in das gesunde Hirngewebe und die Resistenzentwicklung gegenüber genotoxischen Therapien. Obwohl die Patienten mit den derzeitigen Therapien über Jahre hinweg einen stabilen Krankheitsverlauf aufweisen, sind maligne Transformation und Rezidivierung nicht vermeidbar. Eine Stimulierung des Immunsystems, so dass dieses die Tumorzellen kontrolliert, könnte eine Langzeit-Remission der Erkrankung bewirken.

70 – 80 % der diffusen und anaplastischen Gliome tragen eine IDH1-Mutation, die in > 90 % der Fälle in der katalytischen Domäne des Proteins lokalisiert ist und zu einem Aminosäureaustausch (Arg zu His) an Position 132 des Proteins führt (Nomenklatur: IDH1R132H). Sie ist die früheste Mutation in diffusen und anaplastischen Gliomen, so dass alle Tumorzellen – auch in progredienten Tumoren – diese Mutation tragen. Die IDH1R132H-Mutation ist somit charakteristisch für Gliome.

Aus immunologischer Sicht stellt die IDH1R132H-Mutation ein geeignetes Tumorantigen dar, da sie spezifisch in Tumor- nicht aber in gesunden Zellen exprimiert wird. In Patienten mit IDH1R132H-mutierten Gliomen wurden zudem mutationsspezifische T-Zellen und Antikörper detektiert. Dies legt nahe, dass das IDH1R132H-Epitop mutationsspezifisch vom Immunsystem erkannt wird. In humanisierten Mäusen resultierte die präventiv oder therapeutisch angewandte IDH1-Peptidvakzine in einer spezifischen Antitumor-Immunantwort und Wachstumskontrolle von IDH1R132H-mutierten Tumoren ohne toxische Effekte zu bewirken.

Das Ziel dieser Phase I-Studie ist die Untersuchung von Sicherheit, Verträglichkeit und Immunogenität der IDH1-Peptidvakzine in Patienten mit IDH1R132H-mutierten Gliomen ohne 1p/19q-Codeletion und mit Verlust der ATRX-Expression.

Table of Contents

TRIAL SCHEDULE **16**
FLOW CHART **18**
1 INTRODUCTION **21**
1.1 SCIENTIFIC BACKGROUND 21
1.1.1 Definition of Glioma 21
1.1.2 Molecular markers in gliomas 22
1.1.3 Grade III (anaplastic) gliomas (AG) 23
1.1.4 Glioblastomas 24
1.1.5 Biological consequences of the IDH1 mutation 25
1.1.6 Targeted vaccination in gliomas 26
1.1.7 The IDH1 mutation as an immunotherapeutic target 27
1.2 TRIAL RATIONALE 28
1.3 BENEFIT/ RISK ASSESSMENT 30
1.4 REFERENCE COMMITTEES 33
1.4.1 Data Monitoring Committee 33
2 TRIAL OBJECTIVES **34**
2.1 PRIMARY OBJECTIVES 34
2.2 SECONDARY OBJECTIVES 34
2.3 TRANSLATIONAL RESEARCH 35
3 TRIAL DESIGN **35**
4 TRIAL DURATION AND SCHEDULE **36**
5 SELECTION OF PATIENTS **36**
5.1 NUMBER OF PATIENTS 36
5.2 INCLUSION CRITERIA 37
5.3 EXCLUSION CRITERIA 38
5.4 CRITERIA FOR WITHDRAWAL 39
5.4.1 Withdrawal of Patients 39
5.4.2 Handling of Withdrawals 40
5.4.3 Handling of Pregnancy 40
5.4.4 Replacement of Patients 40
5.4.5 Premature Closure of the Clinical Trial or a Single Center 40
5.5 PRIOR AND CONCOMITANT ILLNESSES 41
5.6 PRIOR AND CONCOMITANT MEDICATION 41
6 INVESTIGATIONAL MEDICINAL PRODUCT **42**
6.1 GENERAL INFORMATION ABOUT THE INVESTIGATIONAL MEDICINAL PRODUCT 42
6.1.1 IMP: IDH1 peptide vaccine 42
6.1.2 IMP Ingredients 42
6.1.3 Imiquimod 43
6.1.4 Background Treatment 43
6.2 THERAPEUTIC EFFECTS 43
6.2.1 IDH1 peptide vaccine 43
6.2.2 Vaccine formulation with DMSO/PBS/Montanide® 43
6.3 KNOWN SIDE EFFECTS 44
6.3.1 IDH1 peptide vaccine 44
6.3.2 Peptide vaccines in glioma patients 44
6.3.3 Vaccine formulation with DMSO/PBS/Montanide® 44
6.3.4 Aldara® 45
6.4 DOSAGE SCHEDULE 45
6.4.1 Dosage 45
6.4.2 Dosage Adjustment 46

6.5 SPECIAL PRECAUTIONS WITH IDH1 VACCINATIONS 47
6.6 TREATMENT ASSIGNMENT 48
6.7 RANDOMIZATION AND BLINDING 49
6.8 PACKAGING AND LABELING 49
6.9 SUPPLIES AND ACCOUNTABILITY 49
6.10 COMPLIANCE 50
7 TRIAL METHODS 50
7.1 DESCRIPTION OF STUDY VISITS 50
7.1.1 Study visit 1 (days -105 to -23): Molecular Screening 51
7.1.2 Study visit 2 (day -16 to day -2): Clinical Screening 51
7.1.3 Study visit 3 (day 1): Baseline and 1st vaccination 52
7.1.4 Study visit 4 (day 15 ±3): 2nd vaccination 53
7.1.5 Study visit 5 (day 29 ±3): 3rd vaccination 53
7.1.6 Study visit 6 (day 43 ±3): 4th vaccination 53
7.1.7 Study visit 7 (day 71 ±3): 5th vaccination 53
7.1.8 Study visit 8 (day 99 ±3): 6th vaccination 54
7.1.9 Study visit 9 (day 127 ±3): 7th vaccination 54
7.1.10 Study visit 10 (day 155 ±3): 8th vaccination 55
7.1.11 Study visit 11 (day 183 ±3, 4 weeks ±3 days after last vaccination): End of Treatment (EOT) visit 55
7.1.12 Study visit 12 (day 239 ±3, 12 weeks ±3 days after last vaccination): Safety follow-up visit 56
7.1.13 Study visit 13 (day 323 ±3, 24 weeks ±3 days after last vaccination): End of Study (EOS) visit 56
7.2 METHODS OF DATA AND SAMPLE COLLECTION 57
7.2.1 Data Collection and Handling 57
7.2.2 Sample Collection and Handling 57
7.3 MEASUREMENT OF SAFETY PARAMETERS 59
7.3.1 Performance Status 59
7.3.2 Physical examination and vital signs 59
7.3.3 Mini Mental Status Examination (MMSE) 60
7.3.4 Clinical chemistry, hematology and urinalysis 60
7.3.5 Testing for HIV, HBV/HCV and tuberculis 60
7.3.6 Monitoring of autoimmunity and activation of the immune system 61
7.4 MEASUREMENT OF EFFICACY PARAMETERS 61
7.4.1 Immunogenicity 61
7.5 MEASUREMENT OF FURTHER PARAMETERS 61
7.5.1 Radiological tumor assessment and Intratumoral IDH1R132H enzyme activity . 61
7.5.2 IDH1R132H-reactive T cell and antibody subtypes 62
7.5.3 IDH1R132H immunoreactivity in recurrent tumors 62
7.5.4 HLA Typing 62
7.5.5 IDH1R132H DNA in the peripheral circulation 63
8 PLAN FOR TREATMENT OR CARE AFTER THE TRIAL 63
9 ASSESSMENT OF SAFETY 63
9.1 SPECIFICATION OF SAFETY PARAMETERS 63
9.1.1 Adverse Events 63
9.1.2 Serious Adverse Event 64
9.1.3 Expectedness 64
9.1.4 Suspected Unexpected Serious Adverse Reaction (SUSAR) 64
9.2 PERIOD OF OBSERVATION AND DOCUMENTATION 64
9.2.1 Grading of AEs 65
9.2.2 Coherence between AEs and the IMP and Non-IMPs 65
9.2.3 Outcome of AEs 66

9.2.4 Action taken with the IMP and the immunomodulator ... 66
9.2.5 Action taken with the Non-IMP ... 66
9.2.6 Countermeasures ... 67
9.3 REPORTING OF SERIOUS ADVERSE EVENTS BY INVESTIGATOR ... 67
9.4 EXPEDITED REPORTING ... 67
9.5 SECOND ASSESSMENT OF SERIOUS ADVERSE EVENTS ... 67
9.6 EMERGENCY UNBLINDING ... 68
9.7 EMERGENCY TREATMENT ... 68
10 STATISTICAL CONSIDERATIONS ... 68
10.1 STUDY DESIGN ... 68
10.2 PRIMARY OBJECTIVES AND ENDPOINTS ... 68
10.2.1 Regime Limiting Toxicity (RLT) ... 68
10.2.2 Maximum Tolerated Dose (MTD) ... 68
10.2.3 Evaluation of Immunogenicity ... 68
10.3 SECONDARY OBJECTIVES AND ENDPOINTS ... 69
10.4 TRANSLATIONAL RESEARCH ... 69
10.5 SAMPLE SIZE ESTIMATION ... 70
10.5.1 Sample Size Estimation based on Immunogenicity ... 70
10.5.2 Sample Size Estimation based on Safety ... 71
10.6 PATIENT ALLOCATION ... 71
10.7 PATIENT EVALUABILITY, PATIENT REPLACEMENT AND ANALYSIS POPULATIONS ... 71
10.8 STATISTICAL ANALYSIS ... 72
10.8.1 Primary Analyses ... 72
10.8.2 Analysis of the Secondary Endpoints ... 72
10.8.3 Demographic and other Baseline Characteristics ... 72
10.8.4 Safety Data Analysis ... 72
10.8.5 Clinical Response Rate and Survival Analyses ... 73
10.8.6 Other Analyses ... 73
10.8.7 Translational Research Program ... 73
10.9 HANDLING OF MISSING VALUES ... 73
10.10 INTERIM ANALYSIS ... 73
10.11 MULTIPLE COMPARISONS/ MULTIPLICITY ... 74
10.12 EARLY TRIAL TERMINATION, CONTINUOUS MONITORING OF TOXICITY AND STOPPING RULES ... 74
10.12.1 Early trial termination due to toxicity ... 74
10.12.2 Early trial termination due to shortening of PFS ... 75
11 DATA MANAGEMENT ... 77
11.1 DATA COLLECTION ... 77
11.2 DATA HANDLING ... 77
11.3 STORAGE AND ARCHIVING OF DATA ... 78
12 ETHICAL AND LEGAL ASPECTS ... 78
12.1 GOOD CLINICAL PRACTICE ... 78
12.2 PATIENT INFORMATION AND INFORMED CONSENT ... 78
12.3 CONFIDENTIALITY ... 79
12.4 RESPONSIBILITIES OF INVESTIGATOR ... 79
12.5 APPROVAL OF TRIAL PROTOCOL AND AMENDMENTS ... 79
12.6 CONTINUOUS INFORMATION TO INDEPENDENT ETHICS COMMITTEE ... 80
12.7 NOTIFICATION OF REGULATORY AUTHORITIES ... 80
12.8 REGISTRATION OF THE TRIAL ... 80
12.9 INSURANCE ... 80
13 QUALITY ASSURANCE ... 81
13.1 MONITORING ... 81
13.2 INSPECTIONS/ AUDITS ... 81

14 AGREEMENTS 81
14.1 FINANCING OF THE TRIAL 81
14.2 FINANCIAL DISCLOSURE 81
14.3 REPORTS 81
14.4 PUBLICATION 81
15 SIGNATURES 82
16 DECLARATION OF INVESTIGATOR 83
17 REFERENCES 84
18 APPENDICES 88
APPENDIX I: MEASUREMENT OF SAFETY PARAMETERS 88
Performance Status 88
Mini Mental Status Examination 88
APPENDIX II: DISEASE ASSESSMENT BY RANO CRITERIA 90

Protocol Synopsis

Title

Targeting IDH1R132H in WHO grade III-IV IDH1R132H-mutated gliomas by a peptide vaccine – a Phase I safety, tolerability and immunogenicity multicenter trial (NOA-16)

Phase

I (first-in-man)

Sponsor

Ruprecht-Karls-University Heidelberg, Medical Faculty

- Represented in law by Universitätsklinikum Heidelberg and its Commercial Director Ms. Irmtraut Gürkan -

Im Neuenheimer Feld 672

69120 Heidelberg, Germany

Coordinating Investigator (LKP)

Prof. Dr. med. Michael Platten

Neurology Clinic

University Hospital Heidelberg

Neurooncology Program at the NCT

Im Neuenheimer Feld 400

69120 Heidelberg, Germany

Co-Investigator

Prof. Dr. med. Wolfgang Wick

Neurology Clinic

University Hospital Heidelberg

Neurooncology Program at the NCT

Im Neuenheimer Feld 400

69120 Heidelberg, Germany

Financing/ Status of the Sponsor

This is a non-commercial trial, which is financed by the Neurology Clinic, University Hospital Heidelberg, and the German Cancer Consortium (DKTK).

Indication

C71.1-9

Trial Population

<u>Inclusion Criteria</u>

- Patients present with histologically confirmed diagnosis of an IDH1R132H-mutated glioma (with or without measurable residual tumor after primary tumor resection or biopsy)
- Histology may be astrocytoma, oligodendroglioma, or oligoastrocytoma WHO grade III

or IV

- Absence of chromosomal 1p/19q co-deletion in the tumor tissue
- Loss of ATRX expression in the tumor tissue
- Availability of primary tumor tissue for molecular screening (FFPE bulk tissue or biopsy)
- Patients have received radiotherapy (54 - 60 Gy) alone, 3 cycles of chemotherapy with TMZ (150-200 mg/m^2, 5/28 days) or standard combined radiochemotherapy with TMZ prior to enrollment.
- Patients should be immunocompetent (i.e. no concomitant treatment with dexamethasone (or equivalent), or receive stable/decreasing steroid levels not exceeding 2 mg/day dexamethasone (or equivalent) during the last 3 days prior to clinical screening; no severe lymphopenia)
- ≥18 years old, smoking or non-smoking, of any ethnic origin and gender
- Karnofsky Performance Status ≥ 70
- Ability of patient to understand character and individual consequences of the clinical trial
- Evidence of two informed consent documents personally signed and dated by the patient (or a witness in case the patient is unable to write) covering the molecular screening procedure (*short IC*) and the remaining trial-related procedures (*extended IC*) and indicating that the patient has been informed of all pertinent aspects of the study and that the patient consents to participate in the trial.
- Women of child-bearing potential (WOCBP; i.e., those who have not undergone a hysterectomy, bilateral salpingectomy and bilateral oophorectomy or who have not been post-menopausal for at least 24 consecutive months) must have a negative serum pregnancy test (minimum sensitivity 25 IU/L or equivalent units of HCG) within 72 hours prior to the start of the investigational medicinal product (IMP).
- WOCBP must be using an effective method of birth control to avoid pregnancy throughout the study and for 24 weeks after the last dose of the IMP. This includes two different forms of effective contraception (e.g., hormonal contraceptive and condom, IUD/IUS and condom) or sterilization, resulting in a failure rate less than 1% per year.
- Men must be willing and able to use an effective method of birth control throughout the study for up to 24 weeks after the last dose of the IMP, if their sexual partners are WOCBP (acceptable methods see above).
- Patients who are willing and able to comply with scheduled visits, treatment plan, laboratory tests, and other study procedures

<u>Exclusion Criteria</u>

- Progressive (incl. pseudoprogression) or recurrent disease after radiation therapy, chemotherapy or radiochemotherapy based on local MRI assessment
- Previous or concurrent experimental treatment for the tumor. This includes local therapies such as interstitial radiotherapy or local chemotherapy (i.e. BCNU wafers), loco-regional hyperthermia, and antiangiogenic therapy (such as bevacizumab)
- Antitumor treatment other than standard radiotherapy and/or standard TMZ chemotherapy. Daily metronomic TMZ or intensified dosing scheduled as a substitute for maintenance TMZ cycles are not allowed. (Dose reductions of standard TMZ chemotherapy are allowed.)
- Abnormal (≥ Grade 2 CTCAE v4.0) laboratory values for hematology, liver and renal function (serum creatinine). In detail the following values apply as exclusion criteria:
    a) Hemoglobin < 10 g/dL (6.2 mmol/L)

b) White blood cell count (WBC) decrease (<3.0 x 10^9/L) or increase (>10.0 x 10^9/L)
c) Absolute neutrophil count (ANC) decrease (< 1.5 x 10^9/L)
d) Platelet count decrease (< 75 x 10^9/L)
e) Bilirubin > 1.5 x ULN (upper limit of normal according to the performing lab's reference range)
f) ALT > 3 x ULN
g) AST > 3 x ULN
h) GGT > 2.5 x ULN
i) Serum creatinine increase (> 1.5 x ULN)

- Pregnancy and lactation
- Patients with history or presence of HIV and/or HBV/HCV
- Patients with history or known presence of tuberculosis
- Patients with severe infection(s) or signs/symptoms of infection within 2 weeks prior to the first administration of the study drug
- Patients who have received a live, attenuated vaccine within 4 weeks prior to the first administration of the study drug
- Patients with a prior solid organ transplantation or haematopoietic stem cell transplantation
- History of hypersensitivity to the IMP or to any drug with similar chemical structure or to any excipient present in the pharmaceutical form of the IMP
- Participation in other clinical trials or their observation period during the last 30 days before the first administration of the IMP

Objectives

Primary Objectives

- to determine safety and tolerability of repeated fixed dose vaccinations of the IDH1 peptide vaccine administered with topical imiquimod (Aldara®). Primary safety endpoint is the Regime-Limiting Toxicity (RLT).
- to assess immunogenicity of the IDH1 peptide vaccine and hence demonstrate "proof of principle" for the vaccination strategy. The primary immunogenicity endpoint is the presence of an IDH1R132H-specific T-cell and/or antibody response at any time point during the trial measured by IFN-γ ELISpot and ELISA, respectively (response Yes/No).

Secondary Objectives

- to seek evidence of immunogenicity by assessing the IDH1R132H-specific T-cell and antibody response at visits 3, 5, 7, 10, 12, and 13
- to assess progression-free survival (PFS) and overall response rate (ORR)
- to analyze the association between immunogenicity and the clinical outcome parameters

Translational Research

- to determine magnet resonance spectroscopy (MRS) parameters including R-2-hydroxyglutarate (2-HG) for detection of intra-tumoral IDH1R132H enzyme activity (only if the patient has measurable residual disease, if local neuroradiology has implemented the method, and if baseline MRS data are available for visit 2)

- to characterize the IDH1R132H-reactive T cell and antibody subtypes
- to relate immunogenicity to the HLA type
- to relate immunogenicity and clinical outcome to the presence of IDH1R132H DNA in the peripheral circulation
- to analyze IDH1R132H immunoreactivity in recurrent tumors, if reoperation or biopsy is clinically indicated and tissue is available, and if local laboratory has implemented a protocol for sample processing
- to assess IDH1R132H mutation status in recurrent tumors, if reoperation or biopsy is clinically indicated and tissue is available

Trial Design

This is a non-controlled, open-label, single arm, multicenter first-in-man phase I trial to analyze safety, tolerability and immunogenicity of repeated doses of the IDH1 peptide vaccine in patients with IDH1R132H-positive, non-1p/19q co-deleted, ATRX-negative grade III and IV glioma.

The trial population comprises three treatment groups based on the standard treatment the patient has received / is receiving prior to enrollment: radiotherapy alone (treatment group 1), chemotherapy with TMZ alone (treatment group 2) or combined radiochemotherapy with TMZ (treatment group 3).

Investigational Medicinal Product

The IMP – the IDH1 peptide vaccine – is manufactured by the GMP Core Facility, University Hospital Heidelberg. It consists of a 20-mer peptide encompassing the R132H mutation of IDH1R132H emulsified in Montanide®. The IMP is injected subcutaneously (s.c.) and administered in combination with topical imiquimod (5%, Aldara®).

The Vaccine will be administered in weeks 1, 3, 5, 7, 11, 15, 19 and 23 (visits 3-10).

Sample Size

Sample size estimation is primarily based on the accuracy requirements for the primary endpoint 'immune response' to the IDH1 peptide vaccine. In a second step it has been verified that the intended patient number also fulfills the accuracy requirements for the assessment of the RLT.

30 evaluable patients shall be enrolled into the trial. The sample size will be adjusted for non-evaluable patients (for definition refer to section 5.1), except for drop-outs due to RLT. The corresponding dropout rate is expected to be 20% and thus, 39 patients will have to be recruited for this study. The rate of molecular screening failures for inclusion into this study is expected to be about 55%. Based on this assumption the necessary number of molecularly screened patients will be 87.

Recruitment and treatment of patients will be presumably performed in 8 trial sites, which are part of the German Cancer Consortium (DKTK) and/or the Neurooncology Working Group of the German Cancer Society (NOA).

Statistical Analysis

Summary tables will present the number of patients observed with RLTs and immune responses, the corresponding percentages and exact 95% CIs according to Pearson-Clopper.

All secondary variables and variables of translational research will be analyzed using explorative and mainly descriptive methods. Continuous variables will be summarized using standard summary statistics as appropriate. Summary statistics for categorical variables will

include frequency counts and percentages. If appropriate, graphical presentations of data will be created. Appropriate confidence intervals of effect-estimates will be given to quantify the degree of uncertainty. All statistical tests will be two-tailed with a significance level of 5%. Given the low number of patients and the multiplicity of the analyses all statistical tests are of a strictly exploratory nature.

Trial Duration and Dates

Total trial duration:	51 months
Duration of the clinical phase:	39 months
First patient first visit (FPFV):	May 2015
Last patient first visit (LPFV):	May 2017
Last patient last visit (LPLV):	August 2018
Trial Report Completed:	August 2019

Trial Schedule

Study visit	T/R *	1	2	3	4	5	6	7	8	9	10	11	12	13
		Molecular Screening	Clinical Screening	Vaccination								EOT	Safety follow-up	EOS
Days		-105 to -23 [1)]	-16 to -2 [2)]	1 [3)]	15 ±3	29 ±3	43 ±3	71 ±3	99 ±3	127 ±3	155 ±3	183 ±3 [4)]	239 ±3 [4)]	323 ±3 [4)]
Week		-15 to -3	-2 to -1	1	3	5	7	11	15	19	23	27 (4 weeks after last vaccination)	35 (12 weeks after last vaccination)	47 (24 weeks after last vaccination)
Informed consent														
Informed consent molecular screening (*short IC*)	T	x												
Informed consent (*extended IC*)	T		x											
Molecular screening														
assessment of IDH1 mutation, 1p/19q codeletion and ATRX expression in primary tumor tissue	R/T	x												
Clinical assessments														
Screening ID	T	x												
Patient ID	T		x											
Demographics	R	x												
In- and exclusion criteria	T	x	x											
Medical history incl. concomitant diseases	R		x											
Disease grading/ characteristics; extent of resection/ residual tumor	R	x												
Performance status (KPS) #	R		x	x [5)]				x			x		x	x
Mini Mental Status Examination (MMSE) #	R		x	x [5)]				x			x		x	x
Tumor assessment														
MRI	R		x [6)]					x			x		x	x
Safety assessments														
Physical examination (incl. vital signs, height, weight) [7)] #	R		x	x [5)]	x	x	x	x	x	x	x	x	x	x
12-lead ECG #	R		x	x [5)]				x			x		x	x
Prior and concomitant medication #	R		x	x	x	x	x	x	x	x	x	x	x	x
Adverse events	T			x	x	x	x	x	x	x	x	x	x	x
Laboratory assessments														
HIV, HBV/HCV, Tbc	T		x											
Clinical chemistry #	R		x	x [5)]		x		x		x		x	x	x
Hematology #	R		x	x [5)]		x		x		x		x	x	x
Urinalysis #	R		x	x [5)]		x		x		x		x	x	x
Pregnancy test #	R/T		x	x [5)]		x	x	x	x	x	x	x	x	x
Autoimmunity and activation of the immune system *	T		x	x [5)]		x		x		x		x	x	x
Imunogenicity assessment														
65 ml heparin blood and PBMC isolation #	T			x		x		x			x		x	x
15 ml whole blood and serum preparation #	T			x		x		x			x		x	x
Translational analyses														
MRS parameters (e.g. 2-HG-MRS for IDH1R132H enzyme activity) [8)]	T		x					x			x		x	x
7 ml EDTA blood for HLA typing	T			x [9)]										
65 ml heparin blood and PBMC isolation #	T							x						

Study visit	T/R *	1	2	3	4	5	6	7	8	9	10	11	12	13
		Molecular Screening	Clinical Screening	Vaccination								EOT	Safety follow-up	EOS
**Days**		-105 to -23 [1)]	-16 to -2 [2)]	1 [3)]	15 ±3	29 ±3	43 ±3	71 ±3	99 ±3	127 ±3	155 ±3	183 ±3 [4)]	239 ±3 [4)]	323 ±3 [4)]
Week		-15 to -3	-2 to -1	1	3	5	7	11	15	19	23	27 (4 weeks after last vaccination)	35 (12 weeks after last vaccination)	47 (24 weeks after last vaccination)
8 ml EDTA blood and plasma preparation [#]	T			x		x		x			x		x	x
TIL isolation from fresh tissue of recurrent tumor [10)]	T			x										
FFPE tissue of recurrent tumor for assessment of IDH1 mutation [11)]	T			x										
Study treatment														
Vaccination (s.c. IMP + topical imiquimod)	T			x	x	x	x	x	x	x	x			
Background (standard) treatment														
Radiotherapy	R	x												
TMZ	R	x										x [12]		

EOS = end of study, EOT = End of Treatment, FFPE = Formalin-fixed Paraffin-embedded, HBV/HCV = Hepatitis B/C Virus, HIV = Human Immunodeficiency Virus, KPS = Karnofsky Performance Status, MRI = Magnetic Resonance Imaging, Tbc = Tuberculosis, TIL = Tumor-Infiltrating Lymphocytes, TMZ = Temozolomid

[#] Should be performed prior to vaccination if vaccination is intended at the same visit.

* T = trial-related procedures; R = procedures are performed during clinical routine

[1)] If possible, at clinical routine visit

[2)] After results of molecular screening are available

[3)] Treatment starts at day 1, which corresponds to week 5±1 post radiotherapy (treatment group 1), day 10 ±3 of the 4th TMZ cycle (treatment group 2), and day 10 ±3 of the 1st adjuvant TMZ cycle post concomitant radiochemotherapy (treatment group 3)

[4)] In case of early withdrawal or drop-out: EOT will be performed at withdrawal/drop-out, Safety follow-up and EOS will be performed 12 and 24 weeks after the last vaccination, respectively. If for any reason EOS will be performed < 7 days after Safety follow-up, procedures will not have to be repeated, but data collected at Safety follow-up will serve as EOS data.

[5)] Only necessary, if screening examinations and measurements were perform > 72 hours prior to visit 3

[6)] Treatment groups 1 and 3: According to clinical routine MRI assessment *should not* be performed *earlier* than 4 weeks after the end of radiotherapy, unless clinically indicated.

[7)] Assessment of height is only required at visit 2.

[8)] translational MRS parameters (e.g. 2-HG-MRS) are determined only if method is available at study site, for patients with measurable residual tumor at visit 2 and for patients with baseline MRS data (e.g. 2-HG-MRS) available at visit 2; performed together with MRI session for tumor assessment in the local neuroradiology

[9)] Blood sample for HLA typing could also be collected at any other visit during the trial.

[10)] If patient suffers from recurrence, reoperation or biopsy of the tumor is clinically indicated and TIL isolation is available at clinical site (please note that progressive disease is an indicator for patient withdrawal)

[11)] If patient suffers from recurrence, and reoperation or biopsy of the tumor is clinically indicated (please note that progressive disease is an indicator for patient withdrawal)

[12)] In case of early withdrawal if patient still receives standard treatment

Flow Chart

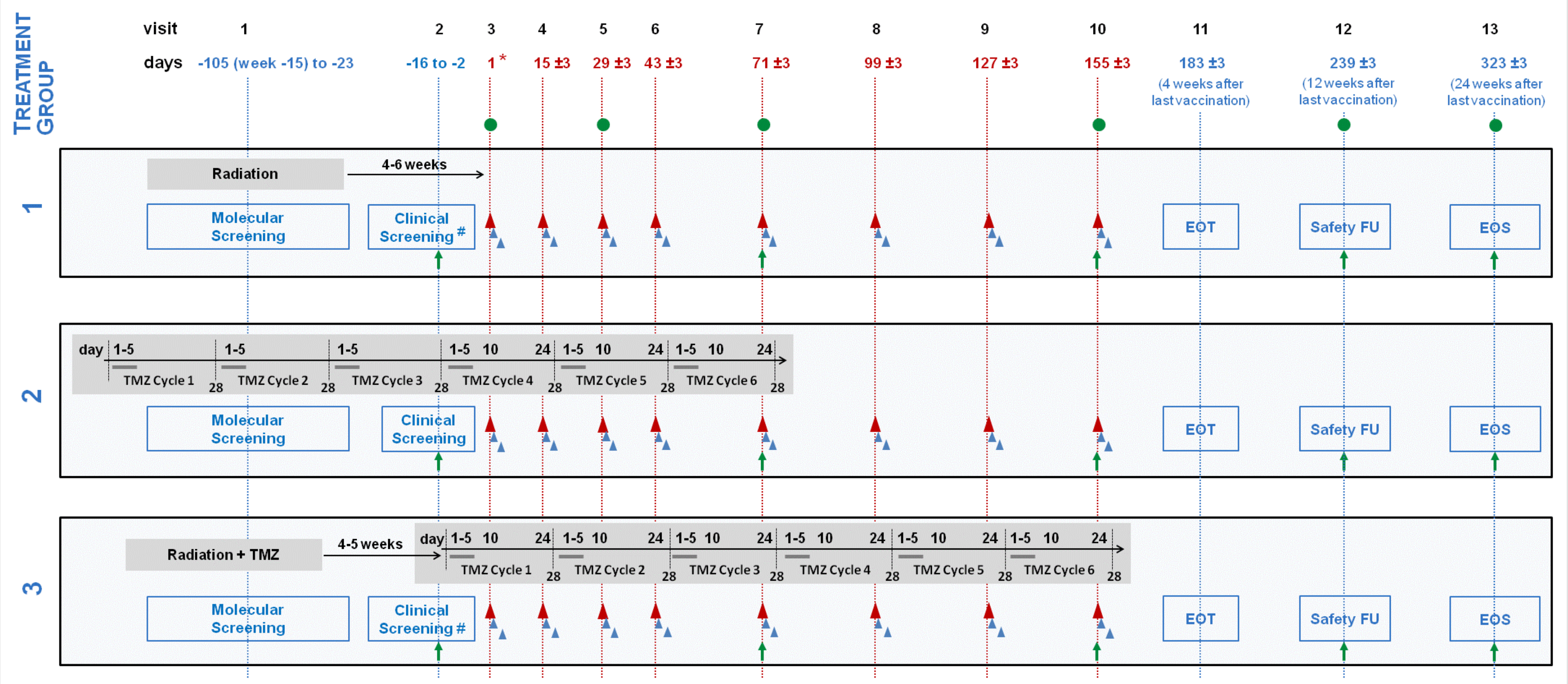

* *day 1 = treatment start:* ***treatment group 1:*** week 5 ±1 post radiotherapy;
treatment group 2: day 10 ±3 of the 4th TMZ cycle;
treatment group 3: day 10 ±3 of the 1st adjuvant TMZ cycle post concomitant radiochemotherapy

\# *Treatment groups 1 and 3:* MRT *should not* be performed *earlier* than 4 weeks after the end of radiotherapy, unless clinically indicated.

▲ IDH1R132H in Montanide (s.c.)
▲ Imiquimod (topical)
↑ MRI assessment
● blood samples for immunogenicity assessment and translational research
Standard Therapy
— TMZ application

Abbreviations

2-HG	R-2-Hydroxy-Glutarate
2-HG-MRS	2-Hydroxy-Glutarate Magnetic Resonance Spectroscopy
90% (95%) CI	90% (95%) confidence interval
AE	Adverse Event
ADL	Activity of Daily Living
AG	Grade III (anaplastic) gliomas
ALT	Alanine Amino Transferase, also known as SGPT
ANC	Absolute Neutrophil Count
AST	Aspartate Amino Transferase, also known as SGOT
ATC	Anatomisch Therapeutisch Chemisches Klassifikationssystem
AMG	German Drug Law (Deutsches Arzneimittelgesetz)
ATRX	α-thalassemia/mental-retardation-syndrome-X-linked
BDSG	Bundesdatenschutzgesetz
BUN	Blood Urea Nitrogen
CI	Coordinating Investigator (LKP)
CRF	Case Report Form
CRP	C Reactive Protein
CT	Computer Tomography
CTCAE	Common Toxicity Criteria for Adverse Events
DBL	Data Base Lock
DKTK	German Cancer Consortium (Deutsches Konsortium für Translationale Krebsforschung)
DMC	Data Monitoring Committee
DMSO	Dimethylsufoxide
DVP	Data Validation Plan
EC	Ethics Committee
ECG	Electrocardiogram
ELISA	Enzyme Linked Immunosorbent Assay
ELISpot	Enzyme Linked Immuno Spot Assay
EMA	European Medicines Agency
EOS	End of Study
EOT	End of Treatment
ESF	Eligibility Screening Forms
FDA	US Food and Drug Administration
FFPE	Formalin-fixed Paraffin-embedded
FPFV	First Patient First Visit
fT4	Tetrajodthyronin / Thyroxin
fT3	Trijodthyronin
FU	Follow-up
GB	Glioblastoma
GCP	Good Clinical Practice

GCP-V	Good Clinical Practice Ordinance (GCP-Verordnung)
GGT	Gamma Glutamyl Transpeptidase
GM-CSF	Granulocyte macrophage colony-stimulating factor
GMP	Good Manufacturing Practice
HBV	Hepatitis B Virus
HCV	Hepatitis C Virus
HIV	Human Immunodeficiency Virus
H-score	Immonohistochemistry Score/Histo Score
IB	Investigator´s Brochure
ICH	International Conference on Harmonization of Technical Requirements for Registration of Pharmaceuticals for Human Use
ICD	International Classification of Disease
i.d.	intradermal
IFA	Incomplete Freund's Adjuvant
IIT	Investigator Initiated Trial
IMP	Investigational Medicinal Product
IMPD	Investigational Medicinal Product Dossier
INN	International Nonproprietary Name
INR	International Normalized Ratio
ISF	Investigator Site File
ISRCTN	International Standard Randomised Controlled Trial Number
ITT	Intention To Treat
IUD	intrauterine device
IUS	intrauterine system
KKS	Coordination Center for Clinical Trials (Koordinierungszentrum für Klinische Studien)
KPS	Karnofsky Performance Status
LDH	Lactate Dehydrogenase
LGG	Grade II (low grade) gliomas
LKP	Coordinating Investigator according to AMG (Leiter der Klinischen Prüfung)
LPFV	Last Patient First Visit
LPLV	Last Patient Last Visit
MCH	Mean Corpuscular Hemoglobin
MCHC	Mean Corpuscular Hemoglobin Concentration
MCV	Mean Corpuscular Volume
MedDRA	Medical Dictionary for Regulatory Activities
MMSE	Mini Mental Status Examination
MRI	Magnetic Resonance Imaging
MRS	Magnetic Resonance Spectroscopy
n.a.	Not applicable
NOA	Neurooncology Working Group of the German Cancer Society (Neuroonkologische Arbeitsgemeinschaft der Deutschen Krebsgesellschaft)

OR	Overall Response
ORR	Overall Response Rate
OS	Overall Survival
PBMC	Peripheral Blood Mononuclear Cells/ Peripheral Blood Monocytes
PBS	Phosphate-buffered Saline
PEI	Paul-Ehrlich-Institut
PT	Prothrombin Time
PTT	Partial Thromboplastin Time
RBC	Red Blood Cells
RLT	Regime-Limiting Toxicity
SAE	Serious Adverse Event
s.c.	subcutaneous
SDV	Source Data Verification
SGPT	Serum Glutamic-Pyruvat Transaminase, also known as ALT
SGOT	Serum Glutamic-Oxaloacetic Transaminase, also known as AST
SOP	Standard Operating Procedure
S(m)PC	Summary of Product Characteristics
SUSAR	Suspected Unexpected Serious Adverse Reaction
Tbc	Tuberculosis
TEAE	Treatment Emergent Adverse Events defined
TIL	Tumor-Infiltrating Lymphocytes
TMF	Trial Master File
TMZ	Temozolomide
TRP	Trial-related Procedure(s)
TSH	Thyreoidea Stimulation Hormon / Thyreotropin
TTP	Time to Progression
ULN	upper limit of normal (according to the performing lab's reference range)
WBC	White Blood Cells
WOCBP	Women of child-bearing potential
WHO	World Health Organization
wt	wildtype

1 Introduction

1.1 Scientific Background

1.1.1 Definition of Glioma

The recent WHO classification of brain tumors [1] maintains the definition of four grades of malignancy for astrocytic and oligodendroglial tumors. The grading is based entirely on histological features. Accordingly, no diagnosis can be made without a surgical procedure to obtain a tissue sample of the tumor.

Pilocytic astrocytoma (WHO grade I) is a tumor mostly encountered in childhood. It is often found in the cerebellum, may reveal large cysts on imaging, and may be cured by resection alone. Diffuse gliomas (WHO grade II) are infiltrative lesions typically localized in the white

matter of the cerebral hemispheres and more rarely in the pons, hypothalamus and spinal cord in adults. Epilepsy is the most common presenting feature. The treatment of these tumors is an area of much controversy. There is a role for surgery, radiotherapy and chemotherapy, but their sequence and timing have remained a matter of debate. Anaplastic gliomas (WHO grade III) and glioblastomas (WHO grade IV) are commonly collectively referred to as malignant gliomas. They may arise anywhere in the brain but the frontal and temporal lobes are most commonly affected.

Diffuse and anaplastic gliomas can be differentiated into astrocytomas and oligodendrogliomas based on their histological appearance. In addition, tumors may bear features of both, oligodendrogliomas and astrocytomas and are then referred to as oligoastrocytomas or mixed gliomas. With respect to prognosis and response to treatment, oligoastrocytomas resemble oligodendrogliomas rather than pure astrocytomas. Numerous studies have now shown that an oligodendroglial differentiation is not only associated with a favorable outcome but also associated with distinct molecular alterations differentiating them from pure astrocytomas. In fact, it has been speculated that astrocytomas and oligodendrogliomas arise from a distinct cell of origin.

1.1.2 Molecular markers in gliomas

Co-deletions on chromosomes 1p and 19q are a feature of oligodendrogliomas and oligoastrocytomas and only very rarely associated with pure astrocytic tumors. These molecular alterations are typically determined using fluorescence in situ hybridization (FISH) or microsatellite polymerase chain reaction (PCR). 1p/19q co-deletions are associated with a favorable prognosis with respect to PFS after genotoxic treatment and to OS as evidenced by several randomized clinical trials (NOA-04, EORTC 26951, RTOG 9402) [2-5]. Long-term analyses of the EORTC 26951 and RTOG 9402 trials looking at the efficacy of combined radiochemotherapy with procarbacine / CCNU / vincristine (PCV) in patients with anaplastic oligodendrogliomas showed that the long-term outcome of patients with co-deletions of 1p/19q was significantly better when treated with combined radiochemotherapy compared with radiotherapy alone. These studies showed for the first time that co-deletion of 1p/19q is not only a prognostic but a predictive marker, indicating better response to combined treatment. As a result patients with anaplastic gliomas and 1p/19q co-deletions are frequently treated with combined radiochemotherapy in clinical practice today.

Mutations and loss of expression of α-thalassemia/mental-retardation-syndrome-X-linked (ATRX) as assessed by immunohistochemistry has been reported to be present in 27% of grade II and 41% of grade III glioma in adult patients. Notably, loss of ATRX expression is more prevalent in astrocytic tumors compared to oligodendroglial tumors and specifically rare in pure oligodendroglioma. Loss of ATRX expression is highly associated with IDH mutation and almost mutually exclusive with 1p/19q co-deletion.

Monoallelic point mutations in the gene for isocitrate dehydrogenase type 1 (IDH1) occur in 70 - 80% of low grade (WHO grade II) and anaplastic (WHO grade III), but only 7% of WHO grade IV gliomas. The vast majority of mutations result in an amino acid exchange from arginine to glutamine at position 132 (IDH1R132H). With the development of a mutation-specific antibody reacting with IDH1R132H but not wild-type IDH1, IDH1R132H can now be faithfully assessed using immunohistochemistry on paraffin-embedded tissue. While mutant IDH1 is associated with a favorable outcome, there is no firm evidence that this molecular marker is predictive for response to a specific therapy. IDH1 mutations are an early event in gliomagenesis even preceding mutations in the TP53 gene. IDH1R132H is associated with a CpG island methylator (CIMP) phenotype.

Hypermethylation of the promoter of the O6-Methylguanine-DNA-Methyltransferase (MGMT) gene can be detected in about 40% of grade II and grade III gliomas. The analysis of MGMT promoter methylation is not yet firmly established in routine diagnosis, partly because of lack of a sufficiently validated test procedure, partly because treatment decision in clinical

practice was not dependent on this information. The most commonly performed procedure is methylation-specific PCR on paraffin-embedded tissue. While in glioblastoma hypermethylation of the MGMT promoter is predictive for response to alkylating chemotherapy, in grade II and grade III gliomas, this alteration is prognostic for a favorable outcome irrespective of the genotoxic treatment modality used. Conceptually, hypermethylation of the MGMT promoter appears to be predictive for response to alkylating chemotherapy only in a setting of an IDH1 wild-type but not IDH1R132H-mutated tumor, explaining the differences between glioblastomas and anaplastic gliomas. As IDH1 mutations result in CIMP including MGMT methylation, there is a strong association of mutant IDH1 with MGMT promoter hypermethylation.

These molecular markers are increasingly implemented into clinical routine. Analyses of several datasets have shown that IDH1-mutated gliomas represent a tumor entity separate from IDH1 wild-type tumors, both with respect to additional genetic alterations and with respect to prognosis. IDH1-mutated gliomas are further sub-classified into tumors with or without co-deletion of 1p/19q. Tumors without 1p/19q co-deletions almost always display loss of ATRX. This subgroup of IDH1R132H-positive, 1p/19q-retained, and ATRX-negative gliomas are almost exclusively of pure astrocytic histology and may be termed „molecular astrocytomas“, even if they contain – by histology – an oligodendroglial component. More importantly, within this subgroup histological grading does not seem to predict outcome.

1.1.3 Grade III (anaplastic) gliomas (AG)

The common practice of care for AG is surgery followed by postoperative radiotherapy alone or chemotherapy alone. The prognostic impact of the surgical resection remains a field of controversy because of the scarcity of prospective clinical data. Although not uniformly assessed by early postoperative imaging as in the 5-aminolevulinic acid (ALA) trial [6], but judged by the neurosurgeon, analysis of surgical data from the NOA-04 trial makes a strong point for the prognostic value of a gross total resection [5]. The German NOA-04 trial analyzed the treatment sequence of radiotherapy and chemotherapy with TMZ or procarbacine, CCNU and vincristine (PCV) in patients with anaplastic gliomas and found no difference in the time to treatment failure of both radio- and one chemotherapy between the group, which started with radiotherapy (42.7 months) and the group, which started with chemotherapy (43.8 months). Here, an oligodendroglial histology (oligodendroglioma or oligoastrocytoma) was a strong predictor of favorable outcome. The standard of care in oligodendroglial tumors is biased by the conclusions that have been drawn from the landmark work of Gregory Cairncross and David Louis introducing procarbazine, lomustine and vincristine (PCV) chemotherapy as well as 1p/19q deletions as a molecular marker into the treatment of these tumors: chemosensitivity – and not as discovered later – therapy sensitivity is the reason for relative success of treating these tumors [7]. Consequently, all large trials in the past years have also focused on PCV. In contrast to glioblastoma [8] and a German trial (NOA 2003) [9], it was felt that sequential radio- and chemotherapy, but not the immediate combination of both could improve outcome. Indeed, long-term analysis of two pivotal trials in anaplastic oligodendroglioma (EORTC26951 and RTOG94-02) has demonstrated that sequential chemoradiotherapy with PCV (irrespective of the sequence) is superior to radiotherapy alone in patients with 1p/19q co-deletion [10]. In fact, the data may suggest that a fraction of patients with 1p/19q co-deleted anaplastic oligodendrogliomas may actually be cured using this regimen.

Besides an oligodendroglial differentiation and 1p/19q co-deletion as prognosticators of favorable outcome, IDH1 and MGMT have been established as additional molecular markers determining outcome based on prospective clinical trials such as NOA-04. Both MGMT promoter hypermethylation and mutant IDH1 are independent prognosticators of favorable outcome regardless of the type of therapy applied [5].

Clearly molecular profiling aids histopathology in determining outcome. In retrospective analyses wild-type IDH1 was an independent prognosticator of unfavorable outcome in patients

with malignant astrocytoma regardless of whether they were grade III or IV [11]. In fact, patients with IDH1 mutant glioblastoma had a more favorable disease course than patients with IDH1 wild-type anaplastic glioma. Despite the ongoing CATNON trial (clinicaltrials.gov: NCT00626990), which assesses the outcome of glioma patients not bearing 1p/19q co-deletions under concomitant and adjuvant TMZ therapy compared to radiotherapy alone, in clinical practice patients with IDH1 wild-type anaplastic astrocytomas are often treated with combined radiochemotherapy like patients with glioblastoma. Analyses of the NOA-08 and the German Glioma Network suggest that only in patients with IDH1 wild-type malignant glioma the MGMT status aid the decision whether or not a patient should receive alkylating chemotherapy [12]. For patients with anaplastic astrocytoma and mutant IDH1, MGMT does not appear to be predictive for response to alkylating chemotherapy, hence in this patient population radiotherapy or chemotherapy alone remains current standard of care until results from the CATNON trial are available.

Also in anaplastic glioma recurrence after genotoxic treatment is inevitable. In the NOA-04 trial PFS was 31 months. Here an oligodendroglial differentiation was associated with a longer PFS (52 months) compared with pure astrocytoma (15 months), too. To date, there is no treatment shown to be effective to prevent or postpone relapse after surgery and genotoxic treatment.

1.1.4 Glioblastomas

Up to 2005, the classical treatment for newly diagnosed glioblastoma included surgical resection when feasible and radiotherapy of the tumor with a peritumoral safety margin of 2-3 cm. Yet, whereas the role of radiotherapy has not been questioned for decades [13], the value of surgical resection was only confirmed in a randomized trial in 2006 by Stummer and colleagues [6]. Using the fluorescent marker 5-aminolevulinic acid to delineate the tumor area under the surgical microscope, they demonstrated an enhanced PFS rate at 6 months in patients who had a complete resection defined by MRI. Chemotherapy in the adjuvant setting was, until recently, largely confined to nitrosourea-based regimens, which conferred a gain in the survival rate at one year from 35% to 41% and at two years from 9% to 13% [14].

The approval of the novel alkylating agent TMZ, which had previously been registered for recurrent anaplastic gliomas [15] and glioblastomas [16], in newly diagnosed glioblastoma around 2005 was probably the most encouraging step ahead in the medical management of malignant gliomas in the last decades. The EORTC 26981-22981 NCIC CE.3 trial compared radiotherapy alone with concurrent and adjuvant TMZ added to radiotherapy. It demonstrated an increase in median survival from 12.1 to 14.6 months and of the 2-year survival rate from 10% to 26% in patients receiving TMZ [17]. In particular, patients with tumors exhibiting methylation of the promoter region of the MGMT gene showed a striking benefit from TMZ [18].

MGMT is a DNA repair enzyme, which is consumed when it is required to repair DNA and therefore classified as a suicide enzyme [19]. It has been suggested for many years that the expression levels of MGMT in the tumor tissue determine the benefit derived from alkylating chemotherapy in patients with malignant gliomas [20,21]. However, it has remained controversial which test is most appropriate to determine the MGMT status.

The emerging role of MGMT in determining resistance to alkylating agents has resulted in various efforts to specifically antagonize this enzyme in the non-responding patient population. Yet, the first efforts to potentiate the clinical activity of nitrosoureas by co-treatment with the MGMT inhibitor, O6-benzylguanine, led, not unexpectedly, to enhanced hematological toxicity, but not to improved tumor control [22].

While differences in MGMT promoter methylation may determine the clinical course in glioblastoma patients treated with TMZ, it is at present not recommended to use the MGMT promoter methylation assay as a clinical guide to decide which glioma patients should receive TMZ and which should not. First, this assay is technically complex, and an independent confirmation of the results of the EORTC NCIC study [18] appears necessary. Second, there is

good reason to believe that alternative, dose-intensified schedules (see below) may produce more benefit for the non-methylators than the conventional 5-out-of-28-days schedule. Third, allocating glioblastoma patients to specific treatments on the basis of MGMT gene promoter methylation status will only assume clinical relevance when effective alternative treatments become available. At present, the only established alternative is nitrosourea-based chemotherapy, which may also depend in its efficacy on the MGMT promoter methylation status [20]. Fourth, it may well be that a differential degree of depletion achieved by continuous TMZ exposure may determine the degree of benefit derived from such treatment in the non-methylators.

IDH1 mutations in primary glioblastomas are rare (approx. 7%). Here, mutant IDH1 is associated with a much more favorable outcome compared with IDH1 wild-type glioblastomas. In fact, IDH1 mutant glioblastomas are associated with a better outcome when compared with IDH1 wild-type anaplastic astrocytomas again underlining the notion that IDH1 mutant gliomas are a separate tumor entity. As mutant IDH1 is associated with a CpG island methylator phenotype (CIMP), there is also a close association with MGMT promoter methylation. Standard of care of patients with IDH1 mutant glioblastomas does not differ from the standard of care of the general glioblastoma population, which is combined radiochemotherapy with TMZ.

1.1.5 Biological consequences of the IDH1 mutation

Since the discovery of monoallelic point mutations in the genes of isocitrate dehydrogenase (IDH) types 1 and 2 in astrocytomas [23-25], acute myeloid leukemias [26,27], sarcomas [28] and other types of tumors, a large body of evidence has accumulated delineating the metabolic consequences of these mutations and their implications in tumorigenesis [29]. IDH1 and IDH2 mutations almost uniformly occur in critical residues in the catalytic site resulting in the inhibition of wild-type (wt) enzymatic activity [30] and a neomorphic dominant enzymatic activity associated with the accumulation of the oncometabolite R-2-Hydroxy-Glutarate (2-HG) [31,32]. This accumulation of 2-HG is sufficient to alter the epigenome of glial and hematopoietic cells [33,34] resulting in a hypermethylation phenotype [35,36], genetic instability, the subsequent acquisition of additional mutations and ultimately malignant transformation [37]. In gliomas and other solid tumors the development of an antibody detecting IDH1R132H [38,39], the most frequent mutated IDH, has not only been implemented in clinical routine diagnostics of gliomas [5] but also guided the concept that IDH1R132H is expressed in all tumor cells constituting this mutation an early event in gliomagenesis even preceding mutations in the TP53 tumor suppressor gene [40]. In grade III astrocytomas and glioblastomas the presence of IDH1R132H is a prognosticator for favorable outcome independent of treatment. In grade III and grade IV astrocytomas pooled the presence of IDH1R132H is a more powerful predictor of favorable outcome than WHO grade. This positive prognostic impact of IDH1R132H does not reflect a tumor suppressive property of IDH1R132H but rather classifies IDH1R132H-mutated tumors as an entirely different tumor entity distinct from IDH1 wild-type tumors. This is also reflected by the fact that IDH1R132H-mutated astrocytoma are associated with a hypermethylator phenotype and young patient age. In low-grade gliomas the prognostic impact of mutant IDH1 is less clear [41].

Collectively, clinical and preclinical data support the view that the IDH1R132H mutation is the first event in gliomagenesis and responsible for genetic instability and subsequent genetic alterations resulting in the formation of diffuse gliomas. Beyond expanding our knowledge on the metabolic and epigenetic control of tumorigenesis and serving as a prognostic parameter in clinical trials [5] the discovery of IDH1 mutations bears important therapeutic implications.

One route is the development of specific inhibitors of the neomorphic enzymatic function of IDH1 and IDH2, which are capable of suppressing tumor growth in preclinical cancer models [42,43]. A different potential strategy may be to explore mutated IDH1 as a cancer immunotherapy target. From an immunological perspective the IDH1R132H mutation represents a potential target for immunotherapy particularly of low grade and anaplastic gliomas as it *(i)* is tumor specific, *(ii)* represents a potential neoantigen with high uniformity and penetrance, and *(iii)* is

expressed in all tumor cells [38,40]. In addition, the development of a mutation-specific antibody in mice [39] suggests that the immune system is – in principle – capable to discriminate between mutant and wild-type IDH1.

Based on recent analyses it seems justified to regard IDH1 mutated glioma as a tumor entity separate from IDH1 wild-type glioma rather than a subgroup with a favorable outcome. Importantly, in patients with IDH1 mutant gliomas the histopathological grading (grade II versus grade III) does not seem to influence prognosis in contrast to patients, who harbor IDH1 wild-type gliomas. Also, when looking at other features important for predicting outcome in IDH1 wild-type gliomas, such as MGMT and proliferation index, these features are not prognostic in IDH1 mutant gliomas.

1.1.6 Targeted vaccination in gliomas

As virtually all glioma patients relapse after resection and standard radio- and/or chemotherapy, induction of tumor-specific immune responses represent an urgently needed and complementary approach.

Firstly, it has been shown that brain tumors are accessible to immune cells including T cells [44]. This is facilitated by a tumor-induced breakdown of the blood-brain barrier [45], but T-cell trafficking through the blood-brain barrier occurs even in healthy brain [46]. Therefore, activated tumor-specific T cells may even reach and efficiently fight tumor cell infiltrates into normal tissues or unresectable portions of the tumor mass. Induction of blood-brain barrier breakdown by irradiation might facilitate the entry of tumor-specific T-cells in the tumor tissue as has been shown for pancreatic cancer [47].

Secondly, T cell-induced tumor cell death is induced by a different mechanism of action from that in radio- and chemotherapy, thus decreasing the chance of malignant cells being able to develop resistance against a therapeutic approach combining both principles. Some evidence for this hypothesis has been provided by Liu and colleagues who demonstrate with clinical and preclinical data that glioblastoma cells are sensitized to chemotherapy after tumor-targeting immunotherapy [48].

Thirdly, targeting a single tumor antigen with a peptide vaccine has been shown to be safe and induce promising immune and clinical responses in GB patients.

According to the Coordinating Investigator´s knowledge, the most advanced cancer vaccine in terms of stage of clinical development for GB is the peptide vaccine Rindopepimut® (CDX-110, Celldex, Needham, MA, USA). It targets the variant III of the epidermal growth factor receptor (EGFRvIII), which like IDH1R132H represents a tumor-specific neoepitope and is, unlike IDH1R132H, expressed in a subset of glioblastomas but not grade II or grade III astrocytoma. In a Phase II study with 22 enrolled patients, CDX-110 vaccine was administered either in combination with standard TMZ maintenance cycles (5/28 at 200 mg/m²/d; N=12) or dose intensified TMZ cycles (21/28 at 100 mg/m²/d; N=10) in newly diagnosed GB patients. In this trial, strong sustained immune responses to EGFRvIII were seen in 100% of evaluated patients: median PFS post-surgery was 15.2 months, and median OS was 23.6 months [49]. CDX-110 treatment was generally well tolerated. Adverse events included one allergic reaction, but no other SAEs were observed. Importantly, the majority of tumors that escaped immunotherapy in the trial had lost EGFRvIII expression.

The promising results have been corroborated in a second Phase II study (ACTIVATE, N=18) [50] and a Phase IIb trial (ACT III, N=65) [51]. In the ACT III trial, Rindopepimut® was administered in combination with standard TMZ maintenance cycles (5/28 at 200 mg/m²/d) to 65 newly diagnosed patients with EGRFvIII-positive GB following tumor resection and chemoradiation. After three initial vaccinations every 2 weeks, Rindopepimut® was administered every 4 weeks for a median of 7.4 months (range: 0.5 – 42.3+ months). Strong immune responses to EGFRvIII

increasing during treatment were observed in 85 % of the patients. PFS post-surgery was 66 % (compared to 45 % estimated from published results for standard of care), and median OS was 21.8 months. Long-term treatment was well tolerated with grade 1 – 2 injection site reactions in almost all patients. 3 SAEs potentially related to Rindopepimut® were observed: toxic epidermal necrolysis, transient grade 2 hypersensitivity reaction, and grade 3 uticarial rash.

A randomized Phase III trial, in which the peptide vaccine is given in addition to conventional radiochemotherapy, is ongoing (ACT IV, www.clinicaltrials.gov: NCT01480479).

Another vaccine, following a different approach, is DCVax-Brain (Northwest Biotherapeutics, Maryland, USA), which has been investigated in two uncontrolled Phase I and I/II trials with 19 newly diagnosed GB patients. In contrast to antigen-specific vaccines, DCVax-Brain uses autologous whole tumor cell lysate loaded ex vivo onto autologous DCs for patient vaccination. Median time to progression (TTP) and median OS were 18.1 and 33.8 months, respectively. The 2-year survival was 68% (compared to 26% with standard care), the 3-year survival 53%, the 4-year survival 35%, and 25% of patients have lived longer than five years [52]. A phase III trial with limited information about the scientific details is currently ongoing (www.clinicaltrials.gov: NCT00045968).

Lastly, based on the immunopeptidome eluted from MHC class I molecules in glioblastoma tissue a multipeptide vaccine (IMA950) has been designed for the treatment of HLA-A2-positive patients with glioblastoma [53]. It includes 12 peptides, 9 HLA-A*02 class I tumor-associated peptides (TUMAPs), an elongated class I TUMAP, one class II TUMAP, and the synthetic Hepatitis B virus marker peptide IMA-HBV-001. IMA950 is investigated in a first-in-man study (Sponsor protocol number: CR0902-11, EUDRACT-2009-015971-28, NCT01222221) in 45 patients with newly diagnosed glioblastoma receiving IMA950 plus GM-CSF introduced at different time points in relation to standard therapy, i.e. TMZ chemoradiotherapy followed by maintenance TMZ therapy. Patients receive up to 11 vaccinations over 24 weeks. Preliminary results show that the vaccine is safe and that the vast majority of patients showed CD8+ vaccine-induced immune response [54].

These results suggest that the combination of chemo(radio)therapy and therapeutic vaccination is safe and feasible including the induction of relevant immune responses. Although most of the clinical data was not generated in randomized trials and a positive outcome compared to historical control has to be viewed with caution, the totality of clinical and immune data clearly supports a potential role of specific immunotherapy in the treatment of glioblastoma.

1.1.7 The IDH1 mutation as an immunotherapeutic target

In the past years we have studied whether a particular mutant IDH1 protein – IDH1R132H – is recognized by the human immune system in a mutation-specific manner [55]. Using peptide libraries encompassing the mutated region of IDH1 we have demonstrated that mutated IDH1R132H peptides were presented on human class II major histocompatibility complexes to stimulate proinflammatory mutation-specific CD4+ T helper 1 responses. Patients with IDH1R132H-mutated gliomas harbor IDH1R132H-specific CD4+ T helper 1 cells and IgG1 antibodies. Furthermore, proximity ligation assays (PLA) have demonstrated that the IDH1R132H epitope colocalizes with MHC class II in IDH1R132H-mutated glioma tissue [56].

In preclinical studies on HLA-humanized A2.DR1 transgenic mice immunization with the IDH1 peptide vaccine, which targets the IDH1R132H mutation, induced a mutation-specific T cell response, which was associated with the generation of mutation-specific anti-IDH1 antibodies.

To test therapeutic efficacy of the IDH1 peptide vaccine in a human MHC context we have chemically induced sarcomas in humanized A2.DR1 transgenic mice. These mice are devoid of mouse MHC class I and II and transgenic for the human MHC I allele A2 and the human MHC II allele DR1. A2.DR1 sarcomas virally transfected with human IDH1R132H and implanted into syngeneic A2.DR1 mice remained static upon vaccination with the IDH1 peptide vaccine while

IDH1 wild-type sarcomas did not respond. This was observed for both pre-established tumors and preventive vaccination. Of note, IDH1R132H+ tumors that escaped the mutation-specific vaccine had lost IDH1R132H expression [55,57].

The IDH1 peptide vaccine is a vaccine designed to elicit an immune response against a unique amino acid sequence present in the IDH1R132H protein. It consists of a peptide of 20 amino acids (p123-142), which spans the mutated region at position 132 of the protein sequence. The IDH1 peptide vaccine includes the adjuvant Montanide® and is designed to be used in combination with immunomodulators to treat malignancies bearing the IDH1R132H mutant form of IDH1.

In preclinical studies, repeat-dose vaccination with the IDH1 peptide vaccine did not induce toxicity beyond skin reactions. It was not effective against tumors expressing wild-type IDH1 and it did not affect enzymatic function of wild-type IDH1/2 in brain and liver indicating that there is neither relevant morphological nor functional cross-reactivity with wild-type IDH1 [55].

To date, the mechanism of action of the IDH1 peptide vaccine is not entirely resolved. In the preclinical studies there was no induction of a mutation-specific CD8+ T cell response. At the same time depletion of CD4+ T cells abrogated the therapeutic effect of the vaccine [55]. These data indicate that the efficacy of the vaccine relies on mutation-specific CD4+ T cells. Possible mechanisms of action include direct cytotoxicity. Tumor-specific CD4+ T cells may exert cytotoxicity sufficient to eradicate tumors in preclinical models [58] and in patients [59]. Alternatively, these cells may release proinflammatory cytokines upon encounter of the antigen in the tumor microenvironment presented on tumor cells and/or tumor-infiltrating antigen-presenting cells and eradicate MHC class II-negative tumor cells via tumor-infiltrating macrophages [60]. Importantly, gliomas differ substantially from other tumor types as they do not grow as solid but rather diffusely infiltrating tumors and as they reside in an immune privileged site. Here, antigen-specific CD4+ T cells rather than CD8+ T cells control tumor formation in preclinical models [61].

Detailed information regarding preclinical studies is described in the latest version of the Investigator´s Brochure.

1.2 Trial Rationale

The patient population of the present trial is molecularly defined and includes IDH1R132H mutant grade III and IV gliomas with absence of co-deletion of 1p/19q and with loss of ATRX expression. These IDH1R132H mutant malignant gliomas represent a separate tumor entity exhibiting a better prognosis than glioblastoma, but are nevertheless incurable. Absence of 1p/19q co-deletion and ATRX loss define a subpopulation of IDH1R132H-mutated gliomas suffering from a poor prognosis (CIMP-A phenotype, astrocytomas) [62]. Furthermore, to avoid potential adverse effects of the vaccine on tumor growth (tolerance induction), only patients with grade III and IV gliomas will be enrolled in this first-in-man trial of the IDH1 peptide vaccine.

Virtually all glioma patients relapse after resection and standard radio- and/or chemotherapy. Based on previous clinical trials (RTOG 9402, EORTC26951, NOA-04), the median progression-free survival in the trial population is estimated to be 24 months [5,10,63].

Of note, there are no approved maintenance therapies capable of preventing or delaying tumor recurrence. Upon tumor recurrence therapeutic options are limited and include reresection, reirradiation and exposure to alkylating chemotherapy. After completion of radio- and/or chemotherapy patients are followed with regular clinical examinations and MRI without any tumor-specific therapy. There is no approved targeted therapy currently available in this patient population. Importantly, patients with recurrent disease often suffer from disease-associated neurological deficits. Hence, there is an urgent need for an effective, safe and durable maintenance therapy in this patient population. Upon recurrence, malignant gliomas have often

acquired a more malignant phenotype, resulting in aggressive growth and resistance to radio- and chemotherapy.

The destruction of residual tumor cells by immune therapy may result in disease stabilization or protection from recurrence and eventually may lead to prolonged survival and direct clinical benefit.

Based on its expression in almost all glioma cells, its tumor-specificity, and its ability to induce a mutation-specific immune response, the IDH1R132H mutation represents an attractive target for immune therapy. In mice, the IDH1 peptide vaccine targeting IDH1R132H results in an anti-tumor immune response specifically directed against IDH1R132H and effective in controlling IDH1R132H-expressing tumors without causing toxicity. In addition, clinical trials with different peptide vaccines on patients with gliomas or other tumors suggest that peptide vaccines are effective and safe in the clinical setting.

The aim of the present phase I trial is to evaluate whether the IDH1 peptide vaccine is safe and immunogenic in patients with IDH1R132H-mutated grade III-IV gliomas without 1p/19q co-deletion and with loss of ATRX. An open label phase I trial design is justified to answer this question.

Upon tumor recurrence the immunological function is often compromised due to the need for steroid therapy and previous exposure to chemotherapy. These factors result in poor patient outcome after recurrence limiting the efficacy of any standard or experimental therapy particularly active immunotherapy, where meaningful antitumor immunity is often induced only after several vaccinations. ***Hence, it is desirable to implement active immunotherapy <u>before</u> disease recurrence with the aim at preventing recurrence***. This concept is currently followed on glioblastoma patients in the phase I GAPVAC trial (clinicaltrials.gov: NCT02149225), two phase I/II trials on the peptide vaccine IMA950 (clinicaltrials.gov: NCT01920191 ,NCT01222221) as well as in two phase III clinical trials – Rindopepimut® (ACT IV) and DCVax® (clinicaltrials.gov: NCT01480479, NCT00045968) – where active immunotherapy is integrated into primary radiochemotherapy after phase II clinical trials demonstrated safety of this approach [49,51,64,65]. Of note, these trials resulted in a ***favourable safety profile and an effective immune response without any indication that the efficacy of primary radiochemotherapy was compromised by active immunotherapy.***

The trial population will comprise three treatment groups based on the standard treatment the patient has received / is receiving: radiotherapy alone (treatment group 1), chemotherapy with TMZ alone (treatment group 2) or combined radiochemotherapy with TMZ (treatment group 3). Based on data from clinical trials and following the current treatment guidelines patients with IDH1-mutated gliomas enrolled in this trial may be treated with any of these treatments.

Superiority of radiochemotherapy over radiotherapy alone has only been demonstrated for 1p/19q-codeleted gliomas, which are excluded from this trial population. ***The decision concerning standard treatment is not influenced by participating in the trial.***

Two lines of observations justify these three primary therapeutic regimens in conjunction with active immunotherapy: *(i)* Anaplastic gliomas may be non-contrast-enhancing reflecting the undisturbed blood-brain barrier (BBB) [66]. Radiotherapy results in the induction of an inflammatory milieu in the tumor tissue and breakdown of the BBB, both of which are desirable effects to enhance the efficacy of antitumor immune responses [67]. *(ii)* Chemotherapy with TMZ often results in lymphopenia, which may preferentially target regulatory T cells [68]. Depletion of regulatory T cells from the peripheral circulation has been demonstrated to enhance antitumor immunity induced by active immunotherapy [69]. ***It is thus justified to combine experimental immunotherapy with standard radiotherapy, chemotherapy or both.***

Based on preclinical and clinical experience, four priming vaccinations at 2-weekly intervals and four maintenance vaccinations at 4-weekly intervals were chosen for this clinical trial.

In previous clinical trials (ACT I-IV, GAPVAC, ICT-107), three to four priming doses of vaccine in 14 day intervals were administered following chemoradiotherapy to allow for the generation of a strong prime immune response against the peptide sequence [49,51,65,70] (www.clinicaltrials.gov: NCT01480479). For GAPVAC very short vaccination intervals are used for priming, i.e. day 1, 2, 4, 8, and 15. However, this is due to the short lived nature of the vaccine in aqueous solution, so that frequent vaccinations are intended to maintain a high antigen level during the first days. For vaccines in Montanide® this is not required due to the depot effect of Montanide®.

In the preclinical experiments three vaccines were given in 14 day intervals. Immunogenicity studies performed in these mice mimicking this intended clinical trial design demonstrated that a strong anti-IDH1R132H immune response could be induced following two priming doses of vaccine and that this immune response was sufficient to control the growth of IDH1R132H-mutated tumors. Importantly, however, this immune response was not sufficient to destroy tumors expressing the mutated antigen arguing for additional booster vaccines to sustain an efficient immune response. In addition, in previous clinical vaccine trials (ACTI-IV, GAPVAC, ICT-107) maintenance vaccination in 4-weekly intervals were given to sustain the immune response.

Vaccination will be performed on days 10 ±3 and 24 ±3 during priming, and on day 24 ±3 during maintenance.

TMZ-based standard therapy in glioma is known to induce lymphopenia; especially during the initial chemoradiotherapy lymphocyte counts drop. Thus, concerns about the compatibility with therapeutic vaccinations can be raised. However, published data by Sampson *et al*. indicate that during maintenance TMZ cycles immunotherapy is possible with high immune responses achieved [49]. Even synergistic effects are discussed: Maintenance TMZ periodically induces a drop in total lymphocytes that creates space for T-cell expansion. Vaccinations at the nadir of T cell counts (around day 22 of each TMZ cycle) may specifically activate vaccine-specific T cells that thus, have an advantage in filling the empty T-cell space. Preliminary immune response data with the multi-peptide vaccine IMA950 in glioblastoma from the ongoing study CR0902-11 applied concurrently to TMZ support this hypothesis as high immune response rates are observed. The concept of vaccinations at the nadir of T cell counts has also been followed in the Rindopepimut® trials, in which vaccination was performed approximately at the 21st day of each TMZ cycle [49,51,65].

1.3 Benefit/ Risk Assessment

Vaccines targeting a strictly tumor-restricted mutation require specific considerations: By selecting mutations that can constitute true neo-antigens that are not subject to strong tolerance mechanism the vaccine may lead to a strong immune response. In preclinical mouse studies using humanized mice, the IDH1 peptide vaccine suppressed the growth of IDH1R132H-mutated sarcomas in a preventive (subsequent tumor inoculation) and therapeutic (pre-establishes tumors) manner.

One major concern could be that T cells directed against tumor-derived mutations may be ***cross-reactive to the respective wild-type sequence***. This concern is toned down by the fact that high affinity T-cell receptors against self-peptides are effectively eliminated through thymic selection. In addition, the preclinical studies involving MHC-humanized mice indicate that the IDH1 peptide vaccine induces a T cell response specifically directed against IDH1R132H but does not show relevant cross-reactivity to wild-type IDH1 and/or organ-related autoimmunity.

Furthermore, IDH1R132H-specific immune response may occur naturally in patients bearing IDH1R132H-mutated gliomas: In 4 of 25 and 5 of 25 patients with IDH1R132H-mutated gliomas

IDH1R132H-specific T cells and IDH1R132H-specific antibodies were detected, respectively [55]. These were not reactive against wild-type IDH1. This indicates that the immune system is – in principle – capable to discriminate between mutant and wild-type IDH1. In the preclinical studies, wild-type IDH1 enzymatic function was unperturbed in brain and liver.

Preclinical in vivo studies on melanoma even suggest that the spontaneous immune response against melanomas can be dominated by mutated antigens, and that T cells reactive to mutated peptides can reach *ex vivo* detectable frequencies of considerable size without showing harmful effects to normal skin/melanocytes while occasionally resulting in objective tumor responses [71,72].

Data from many clinical trials suggest that vaccines targeting mutated antigens, especially if peptide-based, are generally safe. T cell responses against mutated oncoproteins have been observed in melanoma patients and were not reported to result in significant autoimmunity so far [73]. Similarly, a dominant CD8+ T-cell response from a melanoma patient receiving the anti-CTLA4 antibody ipilimumab have been shown to be specific for a mutated, tumor-specific neoantigen [74]. In this patient a marked regression of the tumor, but no signs of autoimmunity have been observed. Importantly, spontaneous CD4+ T cells specific for mutated antigens in melanoma have also been detected in melanoma patients [75].

Even vaccines targeting tumor-associated antigens expressed also in toxicity-relevant healthy tissues (e.g. CEA, PSA, PSAP) or known to be ubiquitously expressed (e.g. Her2neu, p53, k-ras) were applied to hundreds of patients and did not lead to unacceptable toxicity including severe autoimmunity up to date. The first vaccination trials in the USA targeting individual p53, K-ras or VHL mutations showed no evidence of vaccine-induced autoimmunity [76-78]. Similarly, extensive clinical tests have shown that autologous therapies based on dendritic cells loaded with tumor cells do not lead to autoimmunity, despite the presence of multiple mutated antigens in the vaccines.

Clinical experience from other vaccines is considered even more helpful for the risk assessment: Autoimmunity has also not been observed in clinical trials with the off-the-shelf vaccines IMA901, IMA910, and IMA950 after treatment of > 500 glioblastoma patients in total (Immatics, personal communication).

In case of the IDH1 vaccine, which targets a mutated tumor antigen, this risk is only theoretical as the target is only expressed in tumor cells but not in normal tissue. In similar situations such as the EGFRvIII vaccine, which also targets a true tumor-specific neoantigen, no autoimmunity has been reported thus far during the phase I-III development [49,51,65].

Thus, it is not expected that the vaccine causes relevant systemic toxicity.

Toxicity analyses in preclinical mouse models included Balb/c mice and mice with human MHC molecules capable of inducing an IDH1R132H-specific T cell response in a human MHC context [55]. Upon repeat-dose vaccination with the IDH1 peptide vaccine administered with Montanide®, GM-CSF and topical imiquimod (Aldara®) – adjuvants (Montanide® and Aldara®) that will be used in the current clinical trial as well – no toxicity was observed beyond skin reactions, as evidenced by haematological and histopathological studies. There was no specific inflammatory reaction specifically associated with the IDH1 peptide vaccine in liver, lung, heart, intestine or brain in two mouse strains (Balb/c and A2.DR1). No weight loss, splenomegaly or alteration in the hematopoietic system in the peripheral circulation or in bone marrow was observed [55] [partially unpublished work].

Wild-type IDH1 is ubiquitously expressed in many organs including brain, heart, lung, liver, intestine and reproductive organs both in mouse and human. As the amino acid sequence of the IDH1 peptide vaccine is identical in mouse and human the safety data in the preclinical mouse models are expected to predict lack of relevant toxicity in humans. Importantly, the peptide sequence of the IDH1 peptide vaccine are identical to mouse and human mutant IDH1 further indicating that the preclinical toxicity studies are relevant with respect to predicting

toxicity in humans. Finally, the IDH1 peptide is included in the warehouse of the Glioma actively personalized vaccine consortium (GAPVAC, study GAPVAC-101; clinicaltrials.gov: NCT02149225).

A further concern might be the ***development of tolerance to IDH1-mutated tumor cells*** induced by the vaccine, which in turn promotes tumor growth. In preclinical studies there was no indication for vaccine-induced tolerance. There was no induction of IDH1R132H-specific regulatory T cells. Instead, IDH1R132H-specific CD4+ T cells were Th1-polarized [55]. The potential concern of clinically relevant tolerance will be met by *(i)* restricting the trial population to patients with WHO grade III and IV gliomas and by *(ii)* conducting interim analyses of the first 10 (and, if applicable, 20 and 30) patients enrolled in the trial based on the progression-free survival (PFS) to detect unexpected shortening of PFS.

The IDH1 peptide vaccine has not been tested ***in human subjects*** yet. However, clinical studies on glioblastoma patients showed that Rindopepimut® (CDX-110) – a similar peptide vaccine targeting the tumor-specific antigen EGFRvIII – induces strong sustained immune responses to EGFRvIII, which translate into clinical benefit without causing relevant systemic vaccine-related toxicity when administered with TMZ-based radiochemotherapy (for details refer to section 1.1.6). Moreover, clinical trials on other tumor types demonstrated the principal safety of peptide vaccines administered with the same adjuvants/ immunomodulators as planned for the present trial (Montanide® and Imiquimod) (for details refer to section 6.3.3 and 6.3.4).

With respect to ***possible local side effects***, the adjuvants/ immunomodulators (Montantanide® and imiquimod) used in the present trial will cause local skin reactions, which may cause uncomfortness but generally do not account for toxicities > CTCAE grade 2 (details in section 6.3.3 and 6.3.4). With respect to ***possible side effects in the brain***, a vaccine-induced inflammatory reaction in the tumor tissue is a desired consequence and prerequisite for a therapeutic effect. In clinical trials with the EGFRvIII vaccine (Rindopepimut®) no SAEs related to a brain inflammation were reported, even in a setting where the EGFRvIII vaccine efficiently eliminated EGFRvIII expressing cells [49,51,65].

In the present trial repeat vaccinations with the IDH1 peptide vaccine are required to induce a potent antigen-specific T cell response. Based on the preclinical data of the IDH1 peptide vaccine and clinical peptide vaccine trials on patients with gliomas or other tumors described above it is not expected that ***repeat vaccinations*** will increase the risk of relevant AEs.

Thus, the IDH1 peptide vaccine is expected to be safe and might represent an effective immune therapy to prevent or retard tumor relapse in the clinical setting.

In the present trial, vaccination will be implemented in standard radio- and/or chemotherapy. Previous phase II trials on Rindopepimut® [49,51,65,79] and DCVax® [64] did not indicate any ***compromising effect of active immunotherapy on primary radiochemotherapy***. In the present trial, the vaccination schedule for patients receiving combined radiochemotherapy slightly differs from the one used in a phase II trial on Rindopepimut®. In that trial, 3 vaccinations every 2 weeks were implemented before starting TMZ maintenance therapy following concomitant radiochemotherapy and thus, standard treatment was retarded [49]. In the present trial, the standard treatment schedule will be followed, and vaccination will be applied every 2 or 4 weeks during the TMZ-free intervals (in contrast to the Rindopepimut® trial, where it was applied at the same day as TMZ for the dose-intense TMZ regime). Since this strategy should mitigate risk of the vaccine, no further toxicities compared to the Rindopepimut® trials are expected.

However, to minimize the risk of compromising standard treatment in the current trial, vaccination will start after radiotherapy (treatment group 1), 3 cycles of TMZ (treatment group 2) or concomitant radiochemotherapy (treatment group 3) will have been completed. For patients

receiving TMZ, this strategy will help identify (and, if advisable, withdraw) patients with TMZ-related toxicities.

There is no differential toxicity of the vaccine anticipated depending on the primary genotoxic therapy chosen. Regular follow-up visits and routine MRI assessment to monitor efficacy of the primary treatment will ensure that the primary standard treatment will not be compromised.

The toxicity associated with the repeated vaccination will be continuously monitored and early trial termination will be taken into consideration, if the risks or toxicity to which participants are exposed to under study medication are unjustifiable. A monitoring of tumor progression (PFS) will allow for assessment of unexpected shortening of PFS, which is expected to be 24 months in the trial population, and consequently, will allow for early trial termination. Potential autoimmune reactions and activation of the immune system will be continuously monitored. Mitigation strategies and management of potential risks (including anaphylactic reactions, autoimmunity, brain edema, and local reactions) are addressed in this protocol and the IB. Furthermore, the first three patients will be enrolled sequentially to allow for the evaluation of acute toxicity and risk mitigation for the following patients.

Taken together, based on current preclinical and clinical data it is reasonable to assume that the IDH1 peptide vaccine will exhibit low toxicity in the clinical setting. If (S)AEs occur, they will be managed by adequate clinical care, and stopping criteria are implemented in case an unacceptable rate of Regime-Limiting Toxicities (RLT) or tolerance induction (shortening of PFS) will be observed. The IDH1 peptide vaccine is expected to induce immunogenicity in a large fraction of patients that might translate into clinical response. The results will be the basis for subsequent phase II trials.

For the individual patient recurrence is inevitable as the disease is only transiently stabilized by radio- and or chemotherapy. There are no approved or experimental interventions to date capable of preventing disease progression/recurrence. Once recurred tumors are usually rapidly progressive resulting in a dismal prognosis with rapid death. Except for re-operation and/or re-irradiation and/or alkylating chemotherapy there are no approved therapies for recurrent disease. If at all these therapeutic measures result in a very transient stabilization of the disease [80].

Thus, the potential benefits outweigh the potential risks of the study for the individual patient and hence, conduct of the trial is justifiable.

1.4 Reference Committees

In order to monitor specific aspects of the current trial a Data Monitoring Committee (DMC) will be established. The work of Reference Committees will be based on the Guideline on Data Monitoring Committees EMEA/CHMP/EWP/5872/03.

1.4.1 Data Monitoring Committee

The DMC will be composed of independent experts in the fields of neuro-oncology and tumor immunotherapy assessing the trial progress and safety data. The mission of the DMC will be to ensure the ethical conduct of the trial and to protect the safety interests of patients in this trial.

The DMC will meet on a regular basis. Based on its review the DMC will provide the sponsor with recommendations regarding trial modification, continuation or termination. The working procedures of the DMC are described in the DMC charter of this trial.

2 Trial Objectives

The purpose of the study is to assess, whether repeated fixed dose vaccination with the IDH1 peptide vaccine in patients with IDH1R132H-mutated gliomas of grade III and IV is safe and tolerable and results in a measurable IDH1R132H-specific immune response.

2.1 Primary Objectives

The primary objective is to assess the safety, tolerability and immunogenicity of repeated fixed dose vaccinations with the IDH1 peptide vaccine administered with topical imiquimod (Aldara®) in patients with IDH1R132H-mutated gliomas of grade III and IV.

For ***safety assessment***, patients will be medically reviewed at each visit including recording of concomitant medications and AEs. All AEs will be graded according to National Cancer Institute Common Terminology Criteria for Adverse Events (CTCAE) version 4.0.

Primary safety endpoint is the Regime-Limiting Toxicity (RLT). RLT is defined as

- any injection site reaction of CTCAE grade 4
- any injection site reaction of CTCAE grade 3 that persists after two weeks
- any other hypersensitivity, anaphylaxis or local allergic reaction ≥ CTCAE grade 3
- brain edema (CTCAE grade 4)
- autoimmunity ≥ CTCAE grade 3
- ≥ CTCAE grade 3 toxicity to organs other than the bone marrow, but excluding
  - grade 3 nausea
  - grade 3 or 4 vomiting in patients who have not received optimal treatment with anti-emetics
  - grade 3 or 4 diarrhea in patients who have not received optimal treatment with anti-diarrheas
  - grade 3 fatigue
- death

that is definitely/certainly, probably, or possibly related to the administration of the IMP. Patients who experience RLT will be removed from trial treatment. Dose de-escalation of the trial agents is not allowed, but vaccination may be skipped because of AEs (for details see section 6.4.2.1).

Transient fever (> 38 °C) and acute infections are not defined as RLTs, but vaccinations should be skipped in such cases until the patient fully recovered (refer to section 6.4.2.1).

Immunogenicity (Immune response Yes/No) will be assessed for all evaluable patients on blood samples collected at visits 3, 5, 7, 10, 12, and 13. The primary immunogenicity endpoint is the presence of an IDH1R132H-specific T-cell and/or antibody response at any time point during the trial (for details see section 10.2.3). IDH1R132H-specific T-cell and antibody responses are measured on Peripheral Blood Mononuclear Cells (PBMC) using IFN-γ ELISpot and on serum using peptide-coated ELISA, respectively (for details see section 7.4.1).

2.2 Secondary Objectives

The secondary objectives are:

- to seek evidence of immunogenicity by assessing the IDH1R132H-specific T-cell and antibody response measured by IFN-γ ELISpot and ELISA, respectively, at visits 3, 5, 7, 10, 12, and 13,

- to evaluate clinical outcome by assessing the progression-free survival (PFS) and overall response rate (ORR) according to the response evaluation criteria as defined in section 10.3. , and
- to analyze the association between immunogenicity and the clinical outcome parameters.

2.3 Translational Research

In addition to the primary and secondary objectives, a translational research program will

- determine magnet resonance spectroscopy (MRS) parameters including R-2-hydroxyglutarate (2-HG) for detection of intratumoral IDH1R132H enzyme activity (only if the patient has measurable residual disease, if local neuroradiology has implemented the method, and if baseline MRS data is available for visit 2),
- characterize the IDH1R132H-reactive T cell and antibody subtypes,
- relate immunogenicity to the HLA type,
- relate immunogenicity and clinical outcome to the presence of IDH1R132H DNA in the peripheral circulation,
- analyze IDH1R132H immunoreactivity in recurrent tumors, if reoperation or biopsy is clinically indicated and tissue is available, and if local laboratory has implemented a protocol for sample processing, and
- assess IDH1R132H mutation status in recurrent tumors, if reoperation or biopsy is clinically indicated and tissue is available.

Patients can participate in this trial only, if they consent to participate in the translational research as well.

3 Trial Design

This is a non-controlled, open-label, single arm, multicenter first-in-man phase I trial to analyze safety, tolerability and immunogenicity of repeated doses of the IDH1 peptide vaccine in patients with IDH1R132H-positive, non-1p/19q co-deleted, ATRX-negative grade III and IV gliomas.

The trial population will comprise 3 treatment groups based on the standard treatment the patients have received prior to enrollment: radiotherapy alone (treatment group 1), 3 cycles of chemotherapy with TMZ alone (treatment group 2) or combined radiochemotherapy with TMZ (treatment group 3). In treatment group 1 vaccination treatment will be done alone starting 4-6 weeks post radiotherapy. In treatment groups 2 and 3 vaccination treatment will be done in parallel with TMZ chemotherapy starting at day 10 of the 4th cycle of the TMZ monotherapy (treatment group 2) or at day 10 of the 1st TMZ cycle post concomitant radiotherapy (treatment group 3; for details see section 7.1 and the flowchart on p. 18).

Treatment will consist of eight s.c. vaccinations with the IDH1 peptide vaccine in weeks 1, 3, 5, 7, 11, 15, 19 and 23 (visits 3-10). The vaccine will contain the IDH1R132H peptide emulsified in Montanide® and will be administered in combination with topical imiquimod (5%, Aldara®).

For safety assessment, patients will be medically reviewed at each visit. To assess immunogenicity, the occurrence of vaccine-specific T-cell and antibody responses in the peripheral blood will be determined.

4 Trial Duration and Schedule

As described in detail in section 10.5, max. 39 patients will be enrolled in the trial and max. 87 patients will be screened. Expecting a number of 20 eligible patients per year with a consent rate of > 90%, approximately 2 years will be required to recruit the intended number of patients.

Moreover, it is known from experience with other clinical trials that further patients will be referred by hospitals/ clinicians not participating in the trial. Thus, the actual recruiting time is expected to be shorter.

The overall duration of the trial is expected to be approximately 51 months including a clinical period of 39 months (incl. 24 months of recruitment) and a period of 12 months for completing the trial report. Recruitment of patients is expected to start in May 2015. The actual overall duration or recruitment may vary. The end of the clinical trial is defined as the last visit of the last patient (LPLV).

Total trial duration:	51 months
Duration of the clinical phase:	39 months
First patient first visit (FPFV):	May 2015
Last patient first visit (LPFV):	May 2017
Last patient last visit (LPLV):	August 2018
Trial Report Completed:	August 2019

The duration of the trial for each patient is expected to be maximum 62 weeks including molecular and clinical screening, vaccination period as well as end-of-treatment (EOT), Safety follow-up and end-of-study (EOS) visits.

5 Selection of Patients

5.1 Number of Patients

As described in section 10.5, ***30 evaluable patients*** should be enrolled in the clinical trial.

A patient is defined as evaluable if:

- he/she has completed the study up to and including study visit 7, has received at least 4 vaccinations through visit 7 and has all intended blood samples collected for immune monitoring through visit 7, *OR*
- he/she has received at least 6 of 8 vaccinations, and baseline plus two further blood samples collected for immune monitoring through visit 12.

Patient number will be adjusted for non-evaluable patients, except for drop-outs due to RLT. The dropout rate is expected to be 20% (e.g., due to disease progression or reasons listed in section 5.4.1). Thus, ***39 patients will have to be recruited*** for this study.

Due to a high rate (~55 %) of screening failures caused by presence of co-deletion of chromosomes 1p/19q or retaining ATRX expression the expected ***number of patients to be molecularly screened is 87.*** Recruitment and treatment of patients will be presumably performed in 8 trial sites, which are part of the DKTK and/or the NOA. There are no restrictions regarding the number of patients recruited per site.

For safety reasons, the first three patients – irrespective of the treatment group – will be enrolled sequentially: Each patient will receive his first vaccination at the earliest 14 days after the previous patient has received his first vaccination. Before a patient will receive his first vaccination, the Coordinating Investigator will clarify if any unacceptable toxicity has been observed for the previously enrolled patient(s). The data management of the NCT Trial Center

will setup a procedure to ensure that the next patient can only be enrolled if the above mentioned time period has passed and no severe toxicities have been observed.

With the recruitment of the 4th patient, parallel recruitment of patients will be allowed irrespective of the treatment group.

The data management of the NCT Trial Center will setup a ***procedure to ensure that not more than 39 patients will be enrolled.*** To be kept up to date, the NCT Trial Center will be informed about all molecular and clinical screening activities and their results as well as the status of all active patients during the study.

If a patient, who would be included as the 30th evaluable patient, turns out to be eligible in the molecular screening, the following will apply: All further screening activities will be stopped in all centers. Screening will be started again if a patient turns out to be not eligible during clinical screening or to be not evaluable due to drop-out.

Should there be any other patient in molecular screening at the same time, who is considered for inclusion, and who is eligible based on molecular screening, this patient can also be enrolled if the total number of 39 patients is not exceeded.

Moreover, all centers will be informed about the recruitment status on a regular basis.

5.2 Inclusion Criteria

Patients meeting all of the following criteria will be considered for admission to the trial:

- Patients present with histologically confirmed diagnosis of an IDH1R132H-mutated glioma (with or without measurable residual tumor after primary tumor resection or biopsy)
- Histology may be astrocytoma, oligodendroglioma, or oligoastrocytoma WHO grade III or IV
- Absence of chromosomal 1p/19q co-deletion in the tumor tissue
- Loss of ATRX expression in the tumor tissue
- Availability of primary tumor tissue for molecular screening (FFPE bulk tissue or biopsy)
- Patients have received radiotherapy (54 - 60 Gy) alone, 3 cycles of chemotherapy with TMZ (150-200 mg/m^2, 5/28 days) or standard combined radiochemotherapy with TMZ prior to enrollment.
- Patients should be immunocompetent (i.e. no concomitant treatment with dexamethasone (or equivalent), or receive stable/decreasing steroid levels not exceeding 2 mg/day dexamethasone (or equivalent) during the last 3 days prior to clinical screening; no severe lymphopenia)
- $\geq$18 years old, smoking or non-smoking, of any ethnic origin and gender
- Karnofsky Performance Status $\geq$ 70
- Ability of patient to understand character and individual consequences of the clinical trial
- Evidence of two informed consent documents personally signed and dated by the patient (or a witness in case the patient is unable to write) covering the molecular screening procedure (*short IC*) and the remaining trial-related procedures (*extended IC*) and indicating that the patient has been informed of all pertinent aspects of the study and that the patient consents to participate in the trial.
- Women of child-bearing potential (WOCBP; i.e., those who have not undergone a hysterectomy, bilateral salpingectomy and bilateral oophorectomy or who have not been post-menopausal for at least 24 consecutive months) must have a negative serum pregnancy test (minimum sensitivity 25 IU/L or equivalent units of HCG) within 72 hours prior to the start of the IMP.

- WOCBP must be using an effective method of birth control to avoid pregnancy throughout the study and for 24 weeks after the last dose of the IMP. This includes two different forms of effective contraception (e.g., hormonal contraceptive and condom, IUD/IUS and condom) or sterilization, resulting in a failure rate less than 1% per year.
- Men must be willing and able to use an effective method of birth control throughout the study for up to 24 weeks after the last dose of the IMP, if their sexual partners are WOCBP (acceptable methods see above).
- Patients who are willing and able to comply with scheduled visits, treatment plan, laboratory tests, and other study procedures.

5.3 Exclusion Criteria

Patients presenting with any of the following criteria will not be included in the trial:

- Progressive (incl. pseudoprogression) or recurrent disease after radiation therapy, chemotherapy or radiochemotherapy based on local MRI assessment
- Previous or concurrent experimental treatment for the tumor. This includes local therapies such as interstitial radiotherapy or local chemotherapy (i.e. BCNU wafers), loco-regional hyperthermia, and antiangiogenic therapy (such as bevacizumab)
- Antitumor treatment other than standard radiotherapy and/or standard TMZ chemotherapy. Daily metronomic TMZ or intensified dosing scheduled as a substitute for maintenance TMZ cycles are not allowed. (Dose reductions of standard TMZ chemotherapy are allowed.)
- Abnormal (≥ Grade 2 CTCAE v4.0) laboratory values for hematology, liver and renal function (serum creatinine). In detail the following values apply as exclusion criteria:
    a) Hemoglobin < 10 g/dL (6.2 mmol/L)
    b) White blood cell count (WBC) decrease (<3.0 x 10^9/L) or increase (>10.0 x 10^9/L)
    c) Absolute neutrophil count (ANC) decrease (< 1.5 x 10^9/L)
    d) Platelet count decrease (< 75 x 10^9/L)
    e) Bilirubin > 1.5 x ULN (upper limit of normal according to the performing lab's reference range)
    f) ALT > 3 x ULN
    g) AST > 3 x ULN
    h) GGT > 2.5 x ULN
    i) Serum creatinine increase (> 1.5 x ULN)
- Pregnancy and lactation
- Patients with history or presence of HIV and/or HBV/HCV
- Patients with history or known presence of tuberculosis
- Patients with severe infection(s) or signs/symptoms of infection within 2 weeks prior to the first administration of the study drug
- Patients who have received a live, attenuated vaccine within 4 weeks prior to the first administration of the study drug
- Patients with a prior solid organ transplantation or haematopoietic stem cell transplantation
- History of hypersensitivity to the IMP or to any drug with similar chemical structure or to any excipient present in the pharmaceutical form of the IMP
- Participation in other clinical trials or their observation period during the last 30 days before the first administration of the IMP.

No patient will be allowed to enroll in this trial more than once.

5.4 Criteria for Withdrawal

5.4.1 Withdrawal of Patients

A patient must be discontinued from trial treatment for the following reasons:

- At any time at his/her own request (patient's decision to withdraw informed consent for any reason)
- For women, if it becomes known that the patient is pregnant (refer to section 5.4.3)

  All WOCBP should be instructed to contact the investigator immediately if they suspect they might be pregnant (e.g., missed or late menstrual period) at any time during study participation. Pregnancy tests are performed on a regular basis during the trial (refer to trial schedule p 16).
- If the patient suffers a RLT (for definition refer to section 2.1)
- Unequivocal disease recurrence or progression (compared to the baseline MRI prior to initiation of genotoxic therapy based on central disease assessment), if the investigator does not consider continuation of vaccinations and/or further participation in the study to be in the best interest of the patient. In this case, the patient has to agree actively to stay on study and to continue vaccinations. This must be discussed with the Coordinating Investigator.
- Occurrence of exclusion criteria (except for laboratory values)

A patient may be withdrawn from trial treatment for the following reasons:

- If, in the investigator's opinion, continuation of the trial would be detrimental to the patient's well-being
- Occurrence of serious adverse event (SAE) caused by the IMP
- If, in the investigator's opinion, protocol violations caused by the patient would lead to invalid data (e.g. non-compliance with planned study procedures)

The Investigator should decide about withdrawal of patients from trial treatment in case of occurrence of criteria mentioned above after consulting the Coordinating investigator.

A patient must be discontinued from all TRPs (including EOT, Safety follow-up, and EOS visits) for the following reason:

- At any time at his/her own request (patient's decision to withdraw informed consent for any reason)

A patient may be withdrawn from all TRPs (including EOT, Safety follow-up, and EOS visits) for the following reasons:

- Non-adherence to the trial-related requirements, which may (have) influence(d) the validity of the trial data
- If, in light of the circumstances (in particular, non-compliance with TRP) the investigator considers that a continuation of TRP is unfeasible or unacceptable

The Investigator should decide about withdrawal of patients from TRPs in case of occurrence of criteria mentioned above after consulting the Coordinating investigator.

If the patient withdraws from the trial, and also withdraws consent for disclosure of future information, no further evaluations should be performed, and no additional data should be collected. The sponsor may retain and continue to use any data collected before such withdrawal of consent.

5.4.2 Handling of Withdrawals

In all cases, the reason for withdrawal must be recorded in the CRF and in the patient's medical records. In case of withdrawal of a patient at his/her own request, the reason should be asked for as extensively as possible and documented. All efforts will be made to follow up the patient, and all examinations scheduled for the EOT, Safety follow-up, and EOS visits will be performed as indicated in the trial schedule. In case of withdrawal due to recurrence of disease, recurrent tumor tissue should be collected and analyzed if reoperation or biopsy is clinically indicated. For these last examinations and assessments the consent of the patient is necessary and will be requested.

All ongoing (S)AEs of withdrawn patients have to be followed up until no more signs and symptoms are verifiable or the patient is on stable condition.

5.4.3 Handling of Pregnancy

In case of incident pregnancy during the course of the trial, vaccination with the IDH1 peptide vaccine will be discontinued.

Pregnancies, including pregnancies that occur in the female partner of a male study subject, should be treated similar to a serious adverse event and notified to the sponsor within the same timelines (refer to section 9.3) using the pregnancy reporting form. The course of pregnancy should be followed up until delivery or end of any cause, and adverse events associated with pregnancy should be reported (e.g. event in foetus/mother, congenital anomaly/birth defect in the child).

5.4.4 Replacement of Patients

To ensure a sufficient number of patients for appropriate immunogenicity analyses (for details refer to sections 10.5), the patient number will be adjusted for non-evaluable patients (for definition refer to section 5.1; except for drop-outs due to RLT).

5.4.5 Premature Closure of the Clinical Trial or a Single Center

The trial can be prematurely closed or suspended by the Coordinating Investigator / sponsor if new risks for patients become known. Early trial termination will be taken into consideration if continuous monitoring of toxicity or interim analyses of PFS show unjustifiable risks or toxicity of the study medication (for details refer to section 10.12).

The Ethics Committee (EC) and the competent regulatory authorities must then be informed. Furthermore, the Ethics Committee(s) and competent regulatory authorities themselves may decide to stop or suspend the trial.

Should the trial be closed prematurely, all trial material (completed, partially completed, and blank CRFs, IMP, etc.) must be returned to the Coordinating Investigator (CI), the NCT Trial Center Heidelberg or another responsible person designated by the CI.

All involved investigators have to be informed immediately about a cessation/ suspension of the trial. The decision is binding to all trial sites and investigators.

The Coordinating Investigator / sponsor has the right to close a center, at any time, in case of:

- non-compliance with the protocol
- slow recruitment
- poor data quality

If the trial is closed prematurely, the patients shall undergo the EOT, Safety follow-up, and EOS visit for safety reasons. For these last examinations the consent of the patient is necessary and will be requested.

5.5 Prior and Concomitant Illnesses

Relevant additional illnesses present at the time of extended informed consent are regarded as concomitant illnesses and will be documented on the appropriate pages of the CRF. Included are conditions that are seasonal, cyclic, or intermittent (e.g. seasonal allergies; intermittent headache).

Abnormalities which appear for the first time or worsen (intensity, frequency) during the trial are adverse events (AEs) and must be documented on the appropriate pages of the CRF (for detailed information regarding AEs refer to section 9).

5.6 Prior and Concomitant Medication

Relevant additional treatments administered to the patients four weeks prior to clinical screening, on entry to the trial or at any time during the trial are regarded as concomitant treatments and must be documented on the appropriate pages of the CRF.

Any medication which is considered necessary for the well-being of the patient and which is not expected to interfere with the evaluation of the IMP may be given at the discretion of the investigator. All such concomitant medications must be reported in the CRF.

An ***anti-emetic treatment***, for example with 5-HT3 antagonists is common during TMZ therapy and allowed in this study. Lymphopenia is a common side effect of TMZ therapy and standard therapy in glioblastoma has been reported to be associated with cases of pneumocystis. Thus, prophylactic treatment with antibiotics is common and allowed in this study.

Use of ***prophylactic antihistamines*** prior to vaccination with IDH1 peptide vaccine is allowed if judged necessary by the investigator. Investigators may consider such a pretreatment especially at late vaccinations.

Antihistaminic or immunosuppressive treatment as well as ***topical steroids*** to manage side effects of the vaccine are allowed in this study.

As ***corticosteroids*** are known to have immunosuppressive effects, doses should be kept at a minimum during the vaccination phase. At screening for this trial, patients must be steroid-free or on stable or decreasing steroid levels not exceeding 2 mg/day dexamethasone (or equivalent) during the last 3 days prior to clinical screening.

Patients must not receive ***live, attenuated vaccines*** within 4 weeks prior to the first vaccination.

The following ***concomitant medications*** are ***prohibited*** during study participation until the formal last study visit of a patient:

- systemic antitumor treatment other than surgery, radiotherapy and/or TMZ according to the standard treatment
- daily metronomic TMZ as a substitute for maintenance TMZ cycles
- Bevacizumab
- live, attenuated vaccines (e.g. Measels, Polio, Tuberculosis, Smallpox, Varicella Zoster)
- other investigational drugs

If there is any doubt whether a patient should receive any concomitant medication, the investigator should consult the Coordinating Investigator and a mutual decision will be taken on whether the patient can remain in the study, based on potential influences of the concomitant therapy on the patient’s immune response to a vaccination.

If a patient receives a prohibited medication the investigator should contact the Coordinating Investigator to determine whether the patients should be taken off study treatment due to safety considerations. If safety of the vaccine is not deemed to be affected by the prohibited treatment the patient may remain on study granted the prohibited treatment is discontinued. If the patient

is taken off study treatment due to safety concerns, regular safety follow-up visits will be performed.

6 Investigational Medicinal Product

6.1 General Information about the Investigational Medicinal Product

6.1.1 IMP: IDH1 peptide vaccine

The IDH1 peptide vaccine consists of a 20-mer peptide encompassing the R132H mutation of IDH1R132H emulsified in the oil-in water emulsion known as Incomplete Freund's Adjuvant (IFA, Montanide®).

The peptide is delivered to the GMP Core Facility of the University Hospital Heidelberg as a lyophilisate. This lyophilisate is then re-solubilized in dimethylsufoxide (DMSO) and phosphate-buffered saline (PBS) which is subsequently emulsified with Montanide®, which constitutes *per se* an adjuvant.

For a single vaccination 300 µg of the peptide will be emulsified in a total volume auf 1.4 ml Montanide®.

The IMP is injected subcutaneously (s.c.) and administered in combination with topical imiquimod (5%, Aldara®) as a further immunomodulator.

IDH1 peptide vaccine (drug product)

Drug Code:	NA (in-house production)
International Nonproprietary Name (INN):	GWVKPIIIGHHAYGDQYRAT<br>(20mer peptide, single-code amino acid residue sequence) re-solubilized in DMSO/PBS and emulsified in Montanide®
Pharmaceutical formulation:	emulsion for subcutaneous injection
Route of administration:	subcutaneous (s.c.)
Storage conditions:	+2°C to +10°C
Manufacturer:	GMP Core Facility, University Hospital Heidelberg

6.1.2 IMP Ingredients

IDH1R132H peptide (drug substance)

International Nonproprietary Name (INN):	GWVKPIIIGHHAYGDQYRAT<br>(20mer peptide, single-code amino acid residue sequence)
Pharmaceutical formulation:	lyophilisate
Storage conditions:	-20°C+/-5°C
Manufacturer:	"Wirkstoffpeptidlabor", Dept. Immunology, University of Tuebingen, Auf der Morgenstelle 15, 72076 Tuebingen, Germany
Licence Number (Article number):	11831

Montanide ISA 51 VG Sterile, 36362Z

Drug Code:	NA
International Nonproprietary Name (INN):	NA
ATC code, if officially registered:	NA

Pharmaceutical formulation:	Oil-in-water emulsion
Storage conditions:	+20°C+/-5°C
Manufacturer:	SEPPIC (Puteaux Cedex, France)
Licence number /Product code	36362Z
Batch number	2289806 / T14430

6.1.3 Imiquimod

Imiquimod acts as an agonist of the toll-like receptors TLR7 and TLR8 [81] and is used as an immunomodulator in this trial (regarding EMA´s Explanatory Note on Immunomodulators [82], EMEA/CHMP/VWP/244894/2006). Imiquimod (5%) is only supplied by Meda AB and will be purchased from this company for the present trial:

Aldara®

Drug Code (NDC):	29336-610-12
International Nonproprietary Name (INN):	Imiquimod
ATC code, if officially registered:	D06BB10
Pharmaceutical formulation:	cream (ready-to-use sachets containing 12.5 mg of imiquimod in 250 mg cream, 5%)
Route of administration:	Topical, at the site of injection of the vaccine
Storage conditions:	should not be stored above +25°C; sachets should not be reused once opened
Manufacturer:	Meda AB (Solna, Sweden)
Licence Number	EU/1/98/080/001-002

One sachet of Aldara® cream (250 mg) will be applied to an area of 5 x 5 cm around the injection site of the IDH1 peptide vaccine 15 min after vaccination and left on the skin for approximately 8 hours according to the instructions in the SmPC. 24 hours after the vaccination a second sachet of Aldara® will be applied by the patient as instructed above and left on the skin for approximately 8 hours.

6.1.4 Background Treatment

The IDH1 peptide vaccine is administered to patients receiving standard treatment. This standard treatment (TMZ chemotherapy and/or radiotherapy) is considered as background treatment and thus, as Non-IMP (refer to Trial Flowchart, p. 18).

6.2 Therapeutic Effects

6.2.1 IDH1 peptide vaccine

This clinical phase I trial will be the first-in-man study with the IMP. IDH1R132H has been shown to be presented in a human MHC class II context eliciting a mutation-specific Th1 and antibody response, capable of controlling pre-established IDH1R132H-mutant tumors in a syngeneic MHC-humanized tumor model [55].

Further information regarding preclinical studies is described in section 1.1.7 of this protocol and in the latest version of the Investigator´s Brochure.

6.2.2 Vaccine formulation with DMSO/PBS/Montanide®

Numerous trials with T cell epitope peptides derived from cancer antigens have been performed in cancer patients suffering from melanoma, prostate cancer, renal cell carcinoma and leukemia. For an overview see Parmiani *et al*. for solid tumors [83] and Greiner/Schmitt for leukemia [84]. Up to now, a total of more than 2,000 patients world-wide have received peptide

vaccines. In most of the trials, and particularly in the peptide vaccination trials run by Schmitt and Greiner [85,86] the peptide was formulated in oil-in-water emulsion (Montanide®) [87] after resolubilization in DMSO and PBS. In these clinical peptide vaccination trials about 70% antigen-peptide specific T cell responses and 50% clinical responses were observed.

6.3 Known Side Effects

6.3.1 IDH1 peptide vaccine

The present clinical phase I trial will be the first-in-man study with the IMP. Preclinical studies on HLA-humanized A2.DR1 transgenic mice demonstrated that repeat-dose vaccination with IDH1 peptide vaccine administered with imiquimod/GM-CSF induces no toxicities beside skin reactions. Furthermore, repeat-dose vaccination with the IDH1 peptide vaccine did not affect enzymatic function of wild-type IDH1/2 in brain and liver indicating that there is neither relevant morphological nor functional cross reactivity [55].

Detailed information regarding preclinical safety is described in the latest version of the Investigator´s Brochure.

6.3.2 Peptide vaccines in glioma patients

Peptide vaccinations are currently under investigation in clinical trials in patients with brain tumors. These include the EGFRvIII-specific vaccine Rindopepimut® (CDX-110), which is in phase III clinical development (clinicaltrials.gov: NCT01498328, NCT01480479) and the multi-peptide vaccine IMA-950, which is in phase I/II clinical development (clinicaltrials.gov: NCT01920191, NCT01222221). Of note, these and previous trials on the mentioned vaccines use peptides resolubilized in water-for-injection with or without DMSO. No emulsion was used, i.e. peptides were not adjuvanted with Montanide®. Therefore they were administered intradermally (i.d.) instead of subcutaneously (s.c.).

Peptide vaccines in these trials have been generally well tolerated. For Rindopepimut® administered with the standard TMZ dose (n = 95 patients) intended to be used in the present trial, treatment-related AEs have been generally mild to moderate (for details refer to the current version of the IB) [51,65]. Four SARs have been observed: 2 allergic reactions (grade 2 – 3) and 2 skin toxicities (toxic epidermal necrolysis, urticiria; grade 3 – 4) [49,51].

For IMA-950, treatment-related AEs have been generally mild to moderate (CTC of the skin grade 1 to 2; anaphylactic reaction of grade 1 to 3) and have included injection site reactions, fatigue, rash, nausea, pruritus and headache. Injection site reactions are the most observed AEs. Hypersensitivity reactions are also a potential risk, but are typically mild to moderate in severity and transient [88].

Furthermore, the tumor-antigen-loaded dendritic cell vaccine ICT-107 (autologous PBMC-derived dendritic cells pulsed with 6 synthetic peptide CTL epitopes) targeting the tumor and tumor stem cell-associated antigens was generally safe and well tolerated when administered with TMZ in glioblastoma patients [70].

6.3.3 Vaccine formulation with DMSO/PBS/Montanide®

When peptide vaccines formulated with DMSO/PBS/Montanide® were administered to patients with leukemia or solid tumors, CTCAE toxicities were restricted to grade 1 to 2 skin toxicity manifesting as rash and induration of the site of injection. Local granulomas developed and remained for several weeks, but eventually were all resolved. Severe anaphylactic reactions, toxicity of internal organs, development of hypersensitivity as well as autoimmunity have never been reported in Montanide®-based vaccines [86,89-92].

6.3.4 Aldara®

The most common known side effects are described in the current SmPC version of Aldara®.

Imiquimod (Aldara®) has been found to be a ligand for TLR7 and TLR8 thus stimulating T cell responses to given epitope peptides [81]. It has been used as immunomodulator in several clinical peptide vaccination trials: side effects are classically restricted to CTC grade 1 to 2 toxicities of the skin with development of erythema as well as burning and itching at the site of injection of the vaccine/ the application of Aldara® [93-99].

Feyerabend *et al.* demonstrated in an elegant study on peptide vaccination in patients with prostate carcinoma that the combination of peptide plus Aldara® (imiquimod) was non-inferior to the administration of granulocyte-macrophage colony stimulating factor (GM-CSF) [96], which has been used as immunomodulator in combination with peptides emulsified in IFA in many peptide vaccination trials [83,84].

GM-CSF is not any longer available in the European Community (EC) but needs to be imported into the EC. Moreover, GM-CSF was administered on days -2 till +2 around each injection of peptides. Since it needs to be stored at +4°C to +8°C, it imposed logistical problems as patients had to come in several times to the (out-patient) clinic or GM-CSF needed to be shipped to the patients´ home. Eventually, the price for the immunomodulator GM-CSF is 50fold higher than for Aldara®. Thus, imiquimod (Aldara®) is more feasible to be used as an adjuvant/ immunomodulator.

Peptide vaccines emulsified in Montanide® and administered with imiquimod induced no severe (garde 3-4) toxicities in patients with (metastatic) prostate, renal and non-small cell lung cancer [95,96,98]. AEs were mainly restricted to local reactions of grade 1-2 (including erythema, redness, swelling, granulomata formation and itching), moderate fever (<39°C) and grade 1 anaphylactic reactions. No signs of autoimmune disease were observed.

6.4 Dosage Schedule

6.4.1 Dosage

A single vaccination consists of s.c. injection of 300 µg IDH1R132H peptide emulsified in 1.4 ml Montanide®. 15 min later topical imiquimod (5%, Aldara®; 1 sachet) will be applied to an area of 5 x 5 cm around the site of injection of the vaccine. Patients will be instructed to leave Aldara® on the skin for approximately 8 hours and to wash the area where Aldara® was applied with mild soap and water afterwards. 24 hours after vaccination patients will apply another sachet of Aldara® and wash the area approximately 8 hours afterwards as described above.

Vaccinations will be administered into the abdominal skin or thigh. The place for the subsequent injections should be as close as possible to the previous injection site for all vaccinations. Ideally, the same draining lymph node should be targeted for all the vaccinations. Only in case of unacceptable local site reactions to the vaccination or imiquimod the injection site may be changed but should be as close as possible to the original injection site. In such a case, subsequent vaccinations should be applied to this newly chosen vaccination site.

Importantly, the patients have to be observed closely after each administration of vaccine: Every patient has to be observed by qualified personnel for at least 6 hours after the first and the second vaccination for adequate treatment in case of acute reactions. In case the vaccine is well tolerated during these first vaccinations, the observation period can be reduced to 2 hours for the following vaccinations.

Facilities and equipment for resuscitation as well as personnel trained in the management of anaphylaxis have to be in place when performing IDH1 peptide vaccinations to shorten reaction times in case of life-threatening anaphylactic reactions and to treat systemic reactions under the direct supervision of a physician.

Vaccine will be administered four times at a bi-weekly and thereafter at a four-weekly interval, i.e. in weeks 1, 3, 5, 7, 11, 15, 19, and 23.

Maximum duration of treatment: 23 weeks

Dose per injection: 300 µg +/- 20 % peptide in 1.4 ml oil-in-water emulsion

The IMP is provided as a ready-to-use vaccine for s.c. application. It has to be ordered from the GMP Core Facility Heidelberg prior to each vaccination and have to be administered within 24 h after preparation (for details refer to section 6.9).

The rationale for dose selection is based on administered doses of peptides in other peptide vaccine trials, which had been as low as 100 µg or as high as 9 mg peptide (mix) per shot. 100 µg – 3 mg per peptide were used both in single and multi peptide vaccines [92,95,96,98,100].

Several of these clinical trials escalated the dose from 100 µg to 1 mg without a dose-related toxicity or dose-related clinical activity. Low doses risk to be insufficient while higher doses exceeding 1 mg will be less efficient. This effect is most likely due to exhaustion of T cells by overstimulation with too much peptide at one time. We could show that we did not augment the number of clinical and immunological responses in cancer patients by giving 1,000 µg instead of 300 µg peptide per shot [85,86].

In clinical trials performed by Michael Schmitt *et al*. constantly 300 µg peptide per shot has been used [85,86,101,102]. This comprised vaccination with the RHAMM-R3 peptide in patients with hematological malignancies (AML, CLL, MDS, Myeloma), which expressed the tumor antigen RHAMM. Moreover, patients with hematological malignancies received a CMVpp65-peptide vaccine after CMV reactivation post allogeneic stem cell transplantation. Good clinical and immunological responses were observed in more than 70% of the patients. The same applies to trials with several peptides at a dose of 300 µg per peptide in multi-valent peptide vaccines used by colleagues in trials for prostate cancer patients at the Dept. of Urology at the University of Tuebingen [96], where the "Wirkstoffpeptidlabor Universität Tübingen" is also majorly involved.

Moreover, in peptide vaccine trials on glioma/glioblastoma patients 300 – 500 µg peptide were used per shot: the EGFRvIII peptide vaccine trials (ACT-I to ACT-IV) utilized 500 µg peptide [51] (www.clinicaltrials.gov: NCT01480479), and 400 µg per peptide are used in the oligo-peptide vaccine trial GAPVAC (www.clinical trials.gov: NCT02149225; personal communication N. Hilf, Immatics).

In the light of all these peptide vaccination trial, either completed or ongoing, the amount of 300 µg of IDH1 peptide per shot was chosen to be used in the present trial.

6.4.2 Dosage Adjustment

6.4.2.1 IDH1 peptide vaccine

Dose adjustments of the IMP are not allowed.

The IMP should be skipped for the following AEs:

- any ≥ CTCAE grade 2 non-skin related AE including autoimmune disorder
- any ≥ CTCAE grade 3 skin-related AE
- potential new or intensified brain edema not attributable to steroid tapering, radiation therapy or tumor progression
- transient fever (> 38 °C) or acute infections

Importantly, in case the AE is classified as RLT (refer to section 2.1), the patient must be withdrawn from trial treatment.

Restart vaccination at the next scheduled time point per protocol

- if/when the AE(s) resolve(s) to ≤ grade 2 (skin) or ≤ grade 1 (non-skin) severity or return(s) to baseline within 4 weeks of AE onset
- if/when autoimmune disorder has fully resolves, is well controlled or is acceptable as judged by the Investigator (taking observed clinical improvements of the disease into account) within 4 weeks of onset
- if/when brain edema has resolved or reduced to baseline (with or without corticosteroid therapy) within 4 weeks of onset
-

If the AE has not resolved and other criteria for restarting vaccination are not met within 4 weeks of AE onset, vaccination will be discontinued.

6.4.2.2 TMZ chemotherapy

It may be necessary to reduce, discontinue or delay TMZ cycles as follows:

TMZ dose escalation and dose reduction during maintenance TMZ cycles may be indicated as described in the SmPC/prescribing information for TMZ at the discretion of the investigator and after consulting the Coordinating Investigator. Such dose changes of TMZ have no impact on the NOA-16 vaccination schedule.

Discontinuation of TMZ cycles may be indicated in case of

- drops in absolute neutrophil count (< 1.0 x 10^9 /mL)
- absolute platelet count decrease (< 50 x 10^9 /mL)
- other CTCAE grade 3 non-hematological toxicities at the lowest TMZ dose level (100 mg/m²)
- other CTCAE grade 4 non-hematological toxicities at any dose level of TMZ

Planned TMZ discontinuation may occur after 6 TMZ cycles. In case of TMZ discontinuation vaccinations will continue unchanged as if 4-weekly TMZ cycles are continued.

Delayed start of TMZ cycles may be indicated e.g. if absolute neutrophil counts and platelets counts have not recovered to sufficient levels from previous cycles. In case of delayed start of TMZ vaccinations will start/continued as if TMZ cycles have been started. In case of permanent discontinuation of TMZ due to toxicity vaccinations will be continued as scheduled.

6.5 Special precautions with IDH1 vaccinations

- ***Facilities and equipment for resuscitation*** as well as ***personnel trained in the management of anaphylaxis*** have to be in place when performing IDH1 peptide vaccinations to shorten reaction times in case of life-threatening anaphylactic reactions and to treat systemic reactions under the direct supervision of a physician.
- As a precautionary measure patients must be kept under ***medical supervision*** for at least 6 h after each vaccination with IDH1 peptide vaccine (if well tolerated during the first two vaccinations, observation time could be reduced to 2 h) to allow for an adequate observation and adequate treatment in case of potential allergic reactions.
- Use of ***prophylactic antihistamines*** prior to vaccination with IDH1 peptide vaccine is allowed if judged necessary by the investigator. Investigators may consider such a pretreatment especially at late vaccinations.
- In case of ***severe allergic (anaphylactic) reactions***, standardized medical treatment (antihistaminic medication) should be applied (refer to the current version of the IB for instructions of acute management of anaphylactic reactions).
- In patients experiencing ***systemic allergic reactions*** (anaphylaxis, ≥CTCAE grade 3, refer to section 2.1) to the study drug(s), no further vaccinations will be given.

- Special emphasis should be put on any clinical sign of the development of ***autoimmune disease***. Any unclear inflammation (e.g. of the bowel or joints) should be documented meticulously and be followed up closely until resolving.
- In the management of toxicities that are suspected signs of ***autoimmunity*** (≥ CTCAE grade 2) pausing or stopping of vaccinations and immunosuppressive treatment should be considered. Vaccinations may be continued if side effects do not meet RLT criteria (refer to section 2.1), are acceptable as judged by the Investigator (taking observed clinical improvements of the disease into account), well controlled or fully resolved.
- ***Monitoring of autoimmunity and activation of the immune system*** will be performed during the trial as described in section 7.3.6. In case of clinically suspected organ autoimmunity at any time point during the trial patients should be screened for organ-specific autoantibodies. If any of the immune monitoring markers described in section 7.3.6 is increased > 3 x ULN and increase is not attributable to other reasons (e.g. concomitant medication or infection),
  - o detailed analysis of organ-specific autoantibodies and subsequently, appropriate imaging studies (ultrasound, CT, MRI) as well as – if deemed necessary – biopsy will be performed according to the local routine.
  - o immunosuppressive therapy (i.e. corticosteroids) should be initiated, and
  - o IMP may be skipped or discontinued (refer to section 6.4.2.1 for details). If autoimmunity ≥ CTCAE grade 3 is observed, patient will be removed from trial treatment (RLT, refer to section 2.1).
- Special attention should be paid to potential signs of ***autoimmune encephalitis***: Encephalitic patients can be confused, somnolent, anorectic, nervous and may have headaches, fever, and fatigue. More severe symptoms include seizures, convulsions, tremors, paralysis, unconsciousness, hallucinations, visual disturbances, still neck, and memory problems.
- IDH1 peptide vaccinations are likely to cause ***local injection site reactions***. Typically erythematosus skin reactions are expected with pain or tenderness at the injection site, erythema and induration, which are usually transient and mild to moderate and occur in the first week after injection. If necessary, topical steroids like mometasonfuroat (Ecural® cream) can be used to alleviate local inflammatory reactions of the cutis.
- There is early evidence that strong immune responses may induce or intensify ***peritumoral edema*** [103]. Investigators shall give special care to neurological examinations and assessment of KPS and shall consider corticosteroids for rapid reduction of edema. Vaccination may be skipped or discontinued (refer to sections 2.1 and 6.4.2.1).
- A specific antidote for the study drug is not known at present. A tabular summary of risks along with mitigation and management strategies is provided with the IB.

6.6 Treatment Assignment

The trial medication will be administered only to patients included in this trial. All patients will receive the trial medication at the same dose (300 µg IDH1R132H peptide).

Patients withdrawn from the trial retain their identification codes. New patients must always be allotted a new identification code.

To unambiguously identify a patient for all TRP, a unique screening ID and – if the patient fulfills the enrollment criteria – a unique patient ID will be assigned (refer to section 10.6).

6.7 Randomization and Blinding

Not applicable. This is a non-controlled, open-label study; no randomization or blinding will be performed.

6.8 Packaging and Labeling

The trial medication will be packed by the GMP Core Facility of the University Hospital Heidelberg and labeled according to § 5 of the GCP-V. The label will contain the following information (in German):

- for use in clinical trial only
- name, address and contact details of the trial sponsor
- name, amount and route of administration of the IMP
- date of manufacturing and expiry
- EudraCT-Number and Code of Trial Protocol
- Patient's Identification Code
- Instructions on storage conditions

6.9 Supplies and Accountability

The investigator will keep an account of the trial medication and acknowledge the receipt of all shipments of the trial medication. All trial medication must be kept in a locked area with access restricted to designated trial staff. The storage temperature of all trial medication has to be documented on a temperature LOG.

The ***IMP*** must be ordered prior to each vaccination from the GMP Core Facility Heidelberg. It has to be administered within 24 h after preparation. For logistic reasons, the GMP Core Facility Heidelberg should be informed about each scheduled visit at which vaccination is intended. Vaccination can only be performed on days following a working day. If possible, visits should be scheduled for the late morning/ early afternoon (about 10 am to 2 pm) to avoid expiring of the vaccine.

By the latest 48 h prior to the scheduled visit the investigator (or other authorized site personnel) has to fill out the IMP Request Form and fax the filled form to the GMP Core Facility Heidelberg for definite IMP ordering. IMP will be delivered by overnight express in the morning of the vaccination visit. Until administration, IMP must be stored at +2°C to +10°C and temperature must be documented. Immediately prior to vaccination, temperature must be read-out to ensure that the vaccine has been within the allowed temperature range during transport and storage.

If the temperature of the IMP was outside the intended range at any time during transport or storage, it must not be administered to the patient. In such a case, the GMP Core Facility Heidelberg should be contacted to clarify if the IMP is still intact and can be administered to the patient.

Aldara® will be ordered directly by each investigator from the local pharmacy. It must be stored in accordance with manufacturer's instructions (for details refer to the current version of the SmPC) at room temperature but not above +25°C and dry.

The investigator will also keep accurate records of the quantities of trial medication dispensed and used for each patient. The documentation has to include date of vaccination, patient identification, batch/ serial numbers or other identification of trial medication. The site monitor will periodically check the supplies of trial medication held by the investigator to ensure the correct accountability of all trial medication used.

All medication containers will be completely destroyed by the investigator (or other authorized site personnel). All remaining or unused trial medication will be returned to the GMP Core Facility Heidelberg or destroyed by the investigator (or other authorized site personnel). The IMP can be disposed to the chemotherapy waste according to local standards. Destruction and return will be documented. It will be assured that a final report of the drug accountability is prepared and maintained by the investigator.

6.10 Compliance

The IDH1 peptide vaccine will be applied to the patient by the investigator or another authorized study physician and will not be dispensed to the patients. Each vaccination will be documented on the Patient Drug Accountability Form to demonstrate compliance.

One sachet of the immunomodulator Aldara® will be dispensed to the patients after each vaccination (along with handling instructions) to be applied 24 h after the vaccination. The patient has to document each application of Aldara® and its removal by washing on an application plan handed out to the patient.

7 Trial Methods

7.1 Description of Study Visits

Treatment as well as routine and study specific examinations will be conducted according to the trial schedule (p. 16). Study visits are scheduled as in-person visits at the study site.

Two informed consent forms will be used in this study: a short one covering the molecular screening procedure (*short IC*) and, in case the patient is eligible based on the molecular screening results, an extended informed consent covering all remaining TRPs starting from the clinical screening to the EOS visit (*extended IC*).

Treatment will consist of 8 s.c. vaccinations with the IDH1 peptide vaccine at visits 3 to 10 (weeks 1, 3, 5, 7, 11, 15, 19, and 23). Each patient will receive the same dose. Dosing is not a major issue in peptide vaccination studies (refer to section 6.4.1) and the proposed dose is within the range suggested as effective by several comparable trials and used in current trials[88,92-94] (www.clinicaltrials.gov: NCT01498328, NCT01480479, NCT00458601).

The IMP will be ordered prior to each vaccination from the GMP Core Facility Heidelberg and has to be administered within 24 h after preparation (for details refer to section 6.9). Vaccination can only be performed on days following a working day.

The vaccination will start at particular time points after (treatment group 1) or during (treatment groups 2 and 3) the standard therapy:

**Standard treatment**	**Start of vaccination**
Radiotherapy (treatment group 1)	5 ±1 weeks post radiotherapy
Chemotherapy with TMZ (treatment group 2)	day 10 ±3 of the 4th TMZ cycle
Radiochemotherapy with TMZ (treatment group 3)	day 10 ±3 of the 1st adjuvant TMZ cycle post concomitant radiochemotherapy

For safety assessment, patients will be medically reviewed at each visit. Blood samples for immune monitoring will be taken at visits 3, 5, 7, 10, 12, and 13.

If the patient suffers from tumor recurrence at any time during the trial, and reoperation or biopsy of the recurrent tumor is clinically indicated, tissue of the recurrent tumor will be collected (for details see section 7.2.2.6) to isolate tumor-infiltrating lymphocytes (if available at clinical

site) and to evaluate IDH1 mutation status. However, due to progressive disease the patient has to be withdrawn from trial treatment.

The duration of the entire study for a patient will be max. 62 weeks consisting of 13 study-specific in-person visits.

The investigative team at each study site will be responsible for performing all evaluations and recording information in the medical record as well as completing the CRF.

7.1.1 Study visit 1 (days -105 to -23): Molecular Screening

Only patients with IDH1R132H-mutated glioma without 1p/19q co-deletion and with loss of ATRX expression will be included into the trial. IDH1R132H mutation status will be assessed by local routine diagnostics. Only patients carrying the IDH1R132H mutation based on local pathology will be considered to be molecularly screened for the trial.

Prior to the molecular screening, all patients will provide written informed consent to participate in the molecular screening (*short IC*). A screening ID (trial site ID plus consecutive number) will be assigned to all patients signing this informed consent for molecular screening (for details refer to section 10.6).

In- and exclusion criteria will be checked as far as possible and documented including:

- demographics (gender, year of birth, country of birth)
- disease characteristics; extent of resection/ residual tumor
- previous and current treatment of the tumor
- standard treatment (TMZ, radiotherapy)
- history of HIV, HBV/HCV or tuberculosis
- history of solid organ or haematopoietic stem cell transplantation

Molecular screening of 1p/19q co-deletion status and ATRX expression will be performed by the local neuropathology or the Central Neuropathology Laboratory. Centers must decide prior to the site initiation whether they will include patients based on local or central molecular analysis. For central molecular assessments, FFPE tissue samples of the primary tumor will be sent to the Central Neuropathology Laboratory (for details refer to section 7.2.2.1).

Irrespective of the laboratory performing the molecular screening, the NCT Trial Center will be informed about the molecular screening activities by fax. Results of molecular screening should be available within a maximum of 3 weeks after request (local assessment) / sample shipment (central assessment). Results will be reported to the clinical site (in case of central assessment) and to the NCT Trial Center (both in case of local and central assessment).

7.1.2 Study visit 2 (day -16 to day -2): Clinical Screening

Prior to any further trial-related procedures (TRP), all patients will provide written informed consent to participate in the trial (*extended IC*).

Clinical screening will be performed when the results of the molecular screening of all 3 markers are available (for details refer to section 7.1.1) and **includes:**

- perform serum pregnancy test for WOCBP (β-HCG)
- perform tests for HIV, HBV/HCV, and Tbc (refer to section 7.3.5)
- document prior and concomitant medication as well as standard treatment (TMZ)
- document medical history incl. concomitant diseases
- evaluate performance status (KPS, refer to section 7.3.1)

- perform MMSE test (refer to section 7.3.3)
- perform physical examination including vital signs, height and weight (refer to section 7.3.2)
- perform 12-lead ECG
- perform clinical chemistry, hematology and urinalysis (refer to section 7.3.4)
- test for autoimmunity and activation of the immune system (refer to section 7.3.6)
- perform radiological tumor assessment (MRI) and send data together with MRI data prior to any genotoxic therapy to the Central Neuroradiology and the NCT Trial Center / CI for central disease assessment (for details refer to section 7.5.1);

  including translational MRS data (e.g. 2-HG-MRS) if the method is available at the site and if patient has measurable residual disease (refer to section 7.5.1)

  According to clinical routine, MRI assessment in treatment groups 1 and 3 *should not* be performed *earlier* than 4 weeks after the end of radiotherapy, unless clinically indicated (i.e. due to new or more severe neurological symptoms.)
- check inclusion and exclusion criteria
- send Inclusion Fax to the NCT Trial Center

  If all eligibility criteria are met, the NCT Trial Center will assign a unique patient ID, which will be communicated to the clinical site (for details refer to section 10.6)

Importantly, the first three patients – irrespective of the treatment group – will be enrolled sequentially with an interval of at least 14 days between their first vaccinations (refer to section 5.1).

7.1.3 Study visit 3 (day 1): Baseline and 1st vaccination

At study visit 3, the following procedures are performed:

Baseline examinations prior to vaccination

- document concomitant medication and standard treatment (TMZ)

(If screening was performed within the last 72 hours prior to visit 3, the following examinations do not need to be repeated. If not repeated, the values obtained during clinical screening will serve as baseline values.)

- evaluate performance status (KPS, refer to section 7.3.1)
- perform MMSE test (refer to section 7.3.3)
- perform physical examination including vital signs and weight (refer to section 7.3.2)
- perform 12-lead ECG
- perform clinical chemistry, hematology and urinalysis (refer to section 7.3.4)
- test for autoimmunity and activation of the immune system (refer to section 7.3.6)
- perform serum pregnancy test for WOCBP (β-HCG)

Collection of blood samples

- ***prior to vaccination, baseline:*** for immune monitoring and translational research obtain 65 ml heparin blood for PBMC isolation, 15 ml whole blood for serum preparation and 8 ml EDTA blood for plasma preparation (refer to sections 7.2.2.2, 7.2.2.3, and 7.2.2.4)
- ***for HLA typing:*** obtain 7 ml EDTA blood (refer to section 7.2.2.5; if not possible at this visit, blood for HLA typing can also be collected at any other visit during the trial)

Vaccination
For details refer to section 6.4.

Document AEs
For details refer to section 9.

7.1.4 Study visit 4 (day 15 ±3): 2nd vaccination

At study visit 4, the following procedures are performed:

Examinations (prior to vaccination)

- perform physical examination including vital signs and weight (refer to section 7.3.2)
- document concomitant medication and standard treatment (TMZ)

Vaccination
For details refer to section 6.4.

Document AEs
For details refer to section 9.

7.1.5 Study visit 5 (day 29 ±3): 3rd vaccination

At study visit 5, the following procedures are performed:

Examinations (prior to vaccination)

- perform physical examination including vital signs and weight (refer to section 7.3.2)
- document concomitant medication and standard treatment (TMZ)
- perform clinical chemistry, hematology and urinalysis (refer to section 7.3.4)
- test for autoimmunity and activation of the immune system (refer to section 7.3.6)
- perform urine or serum pregnancy test for WOCBP (β-HCG)

Collection of blood sample (prior to vaccination)

- for immune monitoring and translational research obtain 65 ml heparin blood for PBMC isolation, 15 ml whole blood for serum preparation and 8 ml EDTA blood for plasma preparation (refer to sections 7.2.2.2, 7.2.2.3, and 7.2.2.4)

Vaccination
For details refer to section 6.4.

Document AEs
For details refer to section 9.

7.1.6 Study visit 6 (day 43 ±3): 4th vaccination

At study visit 6, the same procedures as described for study visit 4 (refer to section 7.1.4) as well as urine or serum pregnancy test for WOCBP (β-HCG) are performed.

7.1.7 Study visit 7 (day 71 ±3): 5th vaccination

At study visit 7, the following procedures are performed:

Examinations prior to vaccination

- evaluate performance status (KPS, refer to section 7.3.1)
- perform MMSE test (refer to section 7.3.3)
- perform physical examination including vital signs and weight (refer to section 7.3.2)
- perform 12-lead ECG
- perform clinical chemistry, hematology and urinalysis (refer to section 7.3.4)
- test for autoimmunity and activation of the immune system (refer to section 7.3.6)
- perform urine or serum pregnancy test for WOCBP (β-HCG)
- document concomitant medication and standard treatment (TMZ)

Further examinations

- perform radiological tumor assessment (MRI) and send data to the Central Neuroradiology and the NCT Trial Center / CI for central disease assessment (for details refer to section 7.5.1);
  including translational MRS data (e.g. 2-HG-MRS) if method is available at site, if patient had measurable residual disease at visit 2 and if baseline MRS data of the patient is available for visit 2 (refer to section 7.5.1)

Collection of blood sample (prior to vaccination)

- for immune monitoring and translational research obtain 130 ml heparin blood for PBMC isolation, 15 ml whole blood for serum preparation and 8 ml EDTA blood for plasma preparation (refer to sections 7.2.2.2, 7.2.2.3, and 7.2.2.4)

Vaccination
For details refer to section 6.4.

Document AEs
For details refer to section 9.

The status fax for visit 7 will be sent to the data management of the NCT Trial Center immediately after visit 7 will have been performed.

7.1.8 Study visit 8 (day 99 ±3): 6th vaccination

At study visit 8, the same procedures as described for study visit 4 (section 7.1.4) as well as urine or serum pregnancy test for WOCBP (β-HCG) are performed.

7.1.9 Study visit 9 (day 127 ±3): 7th vaccination

At study visit 9, the following procedures are performed:

Examinations (prior to vaccination)

- perform physical examination including vital signs and weight (refer to section 7.3.2)
- document concomitant medication and standard treatment (TMZ)
- perform clinical chemistry, hematology and urinalysis (refer to section 7.3.4)
- test for autoimmunity and activation of the immune system (refer to section 7.3.6)
- perform urine or serum pregnancy test for WOCBP (β-HCG)

Vaccination
For details refer to section 6.4.

Document AEs
For details refer to section 9.

7.1.10 Study visit 10 (day 155 ±3): 8th vaccination

At study visit 10, the following procedures are performed:

Examinations prior to vaccination

- evaluate performance status (KPS, refer to section 7.3.1)
- perform MMSE test (refer to section 7.3.3)
- perform physical examination including vital signs and weight (refer to section 7.3.2)
- perform 12-lead ECG
- document concomitant medication and standard treatment (TMZ)
- perform urine or serum pregnancy test for WOCBP (β-HCG)

Further examinations

- perform radiological tumor assessment (MRI) and send data to the Central Neuroradiology and the NCT Trial Center / CI for central disease assessment (for details refer to section 7.5.1);
  including translational MRS data (e.g. 2-HG-MRS) if method is available at site, if patient had measurable residual disease at visit 2 and if baseline MRS data of the patient is available for visit 2 (refer to section 7.5.1)

Collection of blood sample (prior to vaccination)

- for immune monitoring and translational research obtain 65 ml heparin blood for PBMC isolation, 15 ml whole blood for serum preparation and 8 ml EDTA blood for plasma preparation (refer to sections 7.2.2.2, 7.2.2.3, and 7.2.2.4)

Vaccination
For details refer to section 6.4.

Document AEs
For details refer to section 9.

7.1.11 Study visit 11 (day 183 ±3, 4 weeks ±3 days after last vaccination): End of Treatment (EOT) visit

The EOT visit will take place 4 weeks after the last vaccination (due to regular end of study treatment) or at early withdrawal / termination. The following procedures are performed:

Examinations

- perform physical examination including vital signs and weight (refer to section 7.3.2)
- perform clinical chemistry, hematology and urinalysis (refer to section 7.3.4)
- test for autoimmunity and activation of the immune system (refer to section 7.3.6)
- document concomitant medication and standard treatment (TMZ; if patient still receives standard treatment in case of early withdrawal)

- perform urine or serum pregnancy test for WOCBP (β-HCG)

Document AEs
For details refer to section 9.

The EOT fax and, in case of EOT due to RLT, the RLT fax will be sent to the data management of the NCT Trial Center immediately after EOT or RLT have occurred.

7.1.12 Study visit 12 (day 239 ±3, 12 weeks ±3 days after last vaccination): Safety follow-up visit

A Safety follow-up visit will be performed 12 weeks after the last vaccination (due to regular end of study treatment or early withdrawal / termination). At this visit the following procedures will be performed:

Examinations

- evaluate performance status (KPS, refer to section 7.3.1)
- perform MMSE test (refer to section 7.3.3)
- perform physical examination including vital signs and weight (refer to section 7.3.2)
- perform 12-lead ECG
- perform clinical chemistry, hematology and urinalysis (refer to section 7.3.4)
- test for autoimmunity and activation of the immune system (refer to section 7.3.6)
- perform urine or serum pregnancy test (β-HCG)
- document concomitant medication and standard treatment (TMZ; if patient still receives standard treatment in case of early withdrawal)
- perform radiological tumor assessment (MRI) and send data to the Central Neuroradiology and the NCT Trial Center / CI for central disease assessment (for details refer to section 7.5.1);
  including translational MRS data (e.g. 2-HG-MRS) if method is available at site, if patient had measurable residual disease at visit 2 and if baseline MRS data of the patient is available for visit 2 (refer to section 7.5.1)

Collection of blood sample

- for immune monitoring and translational research obtain 65 ml heparin blood for PBMC isolation, 15 ml whole blood for serum preparation and 8 ml EDTA blood for plasma preparation (refer to sections 7.2.2.2, 7.2.2.3, and 7.2.2.4)

Document AEs
For details refer to section 9.

7.1.13 Study visit 13 (day 323 ±3, 24 weeks ±3 days after last vaccination): End of Study (EOS) visit

The EOS visit will be performed 24 weeks after the last vaccination (due to regular end of study treatment or early withdrawal / termination). At EOS visit, the same procedures are performed as described for study visit 12 (refer to section 7.1.12).

If – for any reason – the EOS visit will be performed < 7d after the Safety follow-up visit, procedures do not have to be repeated. In this case data collected at Safety follow-up visit will serve as EOS data.

The EOS fax will be sent to the data management of the NCT Trial Center immediately after EOS will have occurred.

Further follow-up to assess PFS will not be part of this trial, but will be done during routine follow-up visits usually performed every 12 weeks.

7.2 Methods of Data and Sample Collection

7.2.1 Data Collection and Handling

All findings including clinical and laboratory data will be documented by the investigator or an authorized member of the study team in the patient's medical record and in the case report form (CRF). The investigator at the clinical site is responsible for ensuring that all sections of the CRF are completed correctly and that entries can be verified against source data. The CRF has to be filled out according to the specified CRF Completion Guidelines. The correctness of entries in the CRF will be confirmed by dated signature of the responsible investigator.

For the following parameters the CRF will serve as the source document: KPS.

The original CRF will be timely transferred to the data management of the NCT Trial Center by the responsible monitor. One copy of the CRF will remain at the trial site.

After receipt of the CRF pages at the data management of the NCT Trial Center, all data will be entered in a database as recorded in the CRF, and the completeness, validity and plausibility of data are examined (for details refer to section 11.2). All missing data or inconsistencies will be reported back to the sites and clarified by the responsible investigator (or other authorized site personnel).

Results of central disease assessment performed by the Central Neuroradiology and the Coordinating Investigator as well as results of laboratory analyses performed by the Central Immune Laboratory will be reported electronically to the NCT Trial Center.

7.2.2 Sample Collection and Handling

7.2.2.1 Primary tumor tissue for molecular screening

Molecular screening for IDH1R132H mutation, 1p/19q co-deletion and ATRX expression will be performed by the local neuropathology or the Central Neuropathology Laboratory according to the Standard Operating Procedures (SOPs) of the corresponding laboratory. In case of central assessment, FFPE tissue blocks (from bulk tumor or biopsy) or sections will be sent to the Central Neuropathology Laboratory at room temperature.

Results of the molecular screening analyses will be documented on the Molecular Screening Form and – in case of central assessment – sent to the clinical site at the latest 3 weeks after sample shipment. Irrespective of the laboratory performing the molecular screening, the NCT Trial Center will receive a copy of the Molecular Screening Form including screening results. In case of central assessment, FFPE blocks will be sent back to the clinical site after the analyses will have been performed.

7.2.2.2 Collection of Heparin blood samples and PBMC isolation for immunogenicity analyses

PBMC blood samples will be taken at 6 time points spanning the vaccination schedule (visits 3, 5, 7, 10, 12, and 13 (if visit 13 takes place ≥ 7 days after visit 12)). 65 ml of whole blood will be collected for each PBMC sample. At study visit 7, additional 65 ml of whole blood will be taken from all patients for T cell subset analysis. The whole blood is then transported at room temperature to a local laboratory situated within or in close distance from the clinical site. PBMC

isolation and freezing will be performed according to established procedures by the assigned local laboratories and will be started within 6 h after venipuncture. Detailed handling instructions will be provided with the Investigator Site File (ISF).

All critical consumables will be provided by the Coordinating Investigator or the Central Immune Laboratory. Blood collection, isolation and cryoconservation will be documented in the PBMC Form that will be sent to the Central Immune Laboratory. PBMC samples will be stored in a liquid nitrogen cryostorage or in a -80°C freezer at the clinical sites / local laboratories. They must be transferred to a liquid nitrogen cryostorage within 7 days, and will subsequently be sent to the Central Immune Laboratory on dry ice. If liquid nitrogen cryostorage is not available at the clinical site / local laboratory, PBMC samples must be sent to the Central Immune Laboratory on dry ice within 7 days after preparation.

After arrival at the Central Immune Laboratory, frozen PBMC samples will be transferred into a liquid nitrogen cryostorage system for storage before assays will be performed according to the SOPs of the Central Immune Laboratory.

7.2.2.3 Collection of whole blood as well as serum preparation for immunogenicity analyses

Serum samples will be taken at 6 time points spanning the vaccination schedule (visits 3, 5, 7, 10, 12, and 13 (if visit 13 takes place ≥ 7 days after visit 12)). 15 mL of whole blood will be collected. Detailed handling instructions for serum preparation will be provided with the ISF.

All critical consumables will be provided by the Coordinating Investigator or the Central Immune Laboratory. Blood collection, centrifugation and storage will be documented in the Serum Form that will be sent to the Central Immune Laboratory. Serum samples will be stored at the clinical sites / local laboratories and subsequently be sent to the Central Immune Laboratory on dry ice. After arrival at the Central Immune Laboratory, frozen serum samples will be stored at -80°C until assays will be performed.

7.2.2.4 Collection of EDTA blood and plasma preparation for detection of IDH1R132H DNA in the peripheral circulation

EDTA blood samples (8 ml) will be collected at 6 time points spanning the vaccination schedule (visits 3, 5, 7, 10, 12, and 13 (if visit 13 takes place ≥ 7 days after visit 12)). Detailed handling instructions for plasma preparation will be provided with the ISF.

All critical consumables will be provided by the Coordinating Investigator or the Central Immune Laboratory. Blood collection, centrifugation and storage will be documented in the Plasma Form that will be sent to the Central Immune Laboratory. Plasma samples will be stored at the clinical sites / local laboratories and subsequently be sent to the Central Immune Laboratory on dry ice. After arrival at the Central Immune Laboratory, frozen plasma samples will be stored at -80°C until assays will be performed.

7.2.2.5 Collection of EDTA blood for HLA Typing

HLA Typing is performed once during the study (preferentially at study visit 3).

EDTA blood samples (7 ml) are stored at the clinical site or the local laboratory and will be shipped to the Central Immune Laboratory on dry ice. Detailed handling instructions will be provided with the ISF.

All critical consumables will be provided by the Coordinating Investigator or the Central Immune Laboratory. Blood collection and storage will be documented in the HLA Typing Form that will be sent to the Central Immune Laboratory. After arrival at the Central Immune Laboratory, EDTA blood samples will be stored at -80°C for a maximum of 4 months after venipuncture until assays will be performed.

7.2.2.6 Collection and preparation of recurrent tumor tissue for translational research

If the patient suffers from tumor recurrence at any time during the study, tissue of the recurrent tumor will be collected, if surgery or biopsy of the tumor is clinically indicated.

For ***analysis of tumor-infiltrating lymphocytes (TIL)*** in recurrent tumor tissue, a sample of the fresh tumor tissue will be gently dissociated by the local laboratory within 2 h after explantation and frozen as a single cell suspension. Detailed protocols will be provided with the ISF. TIL isolation will only be done at clinical sites whose local laboratories are able to perform the corresponding protocols.

All critical consumables will be provided by the Coordinating Investigator or the Central Immune Laboratory. Explantation, dissociation and cryoconservation will be documented in the TIL Form that will be sent to the Central Immune Laboratory. TIL samples will be stored preferentially in a liquid nitrogen cryostorage or at -80 °C at the clinical sites / local laboratories. They must be transferred to a liquid nitrogen cryostorage within 7 days, and will subsequently be sent to the Central Immune Laboratory on dry ice. If liquid nitrogen cryostorage is not available at the clinical site / local laboratory, TIL samples must be sent to the Central Immune Laboratory on dry ice within 7 days after preparation.

After arrival at the Central Immune Laboratory, frozen TIL samples will be transferred into a liquid nitrogen cryostorage system for storage until assays will be performed according to the SOPs of the Central Immune Laboratory.

Analysis of the IDH1 mutation status will be performed by the local neuropathology or the Central Neuropathology Laboratory. For central assessment, FFPE tissue blocks of the recurrent tumor (from bulk tumor or biopsy) or tissue sections will be sent to the Central Neuropathology Laboratory at room temperature. FFPE blocks will be sent back after the mutation analysis will have been performed.

7.3 Measurement of Safety Parameters

7.3.1 Performance Status

Performance status will be evaluated at visits 2, 3 (if visit 2 takes place > 72 h before visit 3), 7, 10, 12, and 13 (if visit 13 takes place ≥ 7 days after visit 12) regarding the Karnofsky Performane Score (KPS) [104]. For KPS assessment see appendix I, p. 88.

7.3.2 Physical examination and vital signs

Complete physical examination including vital signs, weight and height (only at visit 2) will be performed at visits 2, 3 (if visit 2 takes place > 72h before visit 3), 4 to 11, 12, and 13 (if visit 13 takes place ≥ 7 days after visit 12).

Complete physical examination include assessment of general appearance, head and neck, eyes, ears, nose, throat, chest, lungs, heart, abdomen, extremities, lymph nodes and skin as well as neurological examination.

Vital sign measurements include resting blood pressure (systolic and diastolic, sitting at least five minutes), resting pulse (sitting at least five minutes) as well as oral or ear body temperature (same method of assessment to be used throughout the study).

Physical examinations and testing of vital signs should be performed at any other time during the trial if clinically indicated.

If abnormal results of physical examinations, assessment of vital signs or weight (e.g. weight loss) are considered as clinically relevant – and thus, as AEs – by the investigator, they should be documented as AEs in the CRF (for detailed information regarding AEs refer to section 9).

7.3.3 Mini Mental Status Examination (MMSE)

MMSE [105] will be performed at visits 2, 3 (if visit 2 takes place > 72h before visit 3), 7, 10, 12, and 13 (if visit 13 takes place ≥ 7 days after visit 12). For details refer to appendix I p. 88.

7.3.4 Clinical chemistry, hematology and urinalysis

Clinical chemistry, hematology and urinalysis will be performed at visits 2, 3 (if visit 2 takes place > 72h before visit 3), 5, 7, 9, 11, 12, and 13 (if visit 13 takes place ≥ 7 days after visit 12), and will include:

**Clinical chemistry**		
• Urea<br>• CRP<br>• Glucose, fasting<br>• Sodium<br>• Potassium<br>• Chloride<br>• Uric acid<br>• Calcium<br>• Total protein	• Phosphate<br>• Magnesium<br>• LDH<br>***Liver function***<br>• Albumin<br>• Total and direct Bilirubin<br>• ALT<br>• AST<br>• GGT	***Renal function***<br>• Creatinine
**Hematology**		
• RBC Count<br>• Platelets<br>• WBC count (absolute)<br>• Hemoglobin<br>• Hematocrit	• RBC Indices:<br>MCV, MCH, MCHC<br>(at visit 2 and if hemoglobin decreases ≥ 2 g/dL compared to baseline/visit 2)<br>• Automated WBC differential (absolute):<br>neutrophils, lymphocytes, monocytes, eosinophils, basophils	***Coagulation***<br>• PT<br>• PTT<br>• INR
**Urinalysis**		
• Color<br>• Appearance<br>• pH<br>• specific gravity	• glucose<br>• protein<br>• ketones	• blood<br>• leukocyte esterase<br>• urobilinogen

If abnormal laboratory values are considered as clinically relevant – and thus, as AEs – by the investigator, they should be documented as AEs in the CRF (for information regarding AEs refer to section 9).

7.3.5 Testing for HIV, HBV/HCV and tuberculis

HIV, HBV and HCV serology will be performed at clinical screening according to local standards.

Testing for tuberculosis will be performed at clinical screening by QuantiFERON®-TB Gold test or tuberculin skin test. Patients with an indeterminate result of the QuantiFERON®-TB Gold

test are not eligible unless additional testing demonstrates a negative result (tuberculin skin test or repeated QuantiFERON®-TB Gold test).

If a tuberculin skin test is performed, an induration of > 6 mm is 'positive' for a patient with history of BCG vaccine, while an induration of > 10 mm is 'positive' for a patient without history of BCG vaccine. If necessary a QuantiFERON®-TB Gold test might be complemented by additional specific diagnostic tests as per standard procedures.

7.3.6 Monitoring of autoimmunity and activation of the immune system

To screen for potential autoimmunity and activation of the immune system, all patients

- are tested for serum
  - antithyroglobulin antibodies
  - rheumatoid factor
  - antinuclear antibodies
  - thyroid-stimulating hormone
  - free T3 (fT3)
  - free T4 (fT4)
  - erythrocyte sedimentation rate

  at visits 2, 3 (if visit 2 has taken place > 72h before visit 3), 5, 7, 9, 11, 12, and 13 (if visit 13 takes place ≥ 7 days after visit 12).

7.4 Measurement of Efficacy Parameters

7.4.1 Immunogenicity

Immunogenicity will be assessed by the Central Immune Laboratory based on PBMC and serum samples collected within the study following the SOPs of the Central Immune Laboratory.

PBMCs will be analyzed for the occurrence of IDH1R132H-specific T-cell responses using IFN-γ Elispot. Serum samples will be analysed for IDH1R132H-specific antibodies using ELISA.

7.5 Measurement of Further Parameters

7.5.1 Radiological tumor assessment and Intratumoral IDH1R132H enzyme activity

Disease assessment will be performed by routine 3-monthly MRI according to the RANO criteria [106] by central review as explained below. If it is unclear whether the patient is stable or has developed progressive disease (PD), MRI should be repeated after 4 weeks as part of the clinical routine procedure. In this case, vaccinations will continue until MRI confirms PD. In case repeated MRI confirms PD, the patient will be withdrawn from trial treatment (for details refer to sections 5.4.1 and 5.4.2); otherwise vaccination will be continued according to the trial schedule.

All MRIs are part of the routine follow-up of the patients and do not represent TRP.

As a TRP and if available at study site, translational MRS parameters will be measured including R-2-hydroxyglutarate (2-HG) to assess IDH1R132H enzyme activity as explained below.

7.5.1.1 Survival and Response Rate Analyses

Local response evaluation at the clinical site is used for standard monitoring of the patients and is done based on the routine procedures of the local (neuro-) radiology. Furthermore, for central disease assessment according to the RANO Criteria [106] (refer to Appendix II, p. 90), radiological images are sent to the Central Neuroradiology and required information for clinical integration is sent to the NCT Trial Center / Coordinating Investigator.

Results of the central disease assessment will be used for all PFS monitoring activities and analyses within this trail, including withdrawal of patients due to PD, interim analysis of Progression-free survival (PFS) as well as statistical analyses regarding PFS and Overall Response Rate (ORR).

Results of central radiology assessment by the Central Neuroradiology and of clinical integration by the Coordinating Investigator regarding safety monitoring will be reported back within 3 weeks to the clinical site as well as to the NCT Trial Center. The decision to continue treatment can be based on the local neuroradiological assessment. The decision to discontinue treatment will be based on central disease assessment. In case of discrepancies between local and central assessment, the central assessment is the leading one; where required, the local investigator may contact the Coordinating Investigator to discuss discrepancies and further procedure.

Results of central radiology assessment by the Central Neuroradiology and of clinical integration by the Coordinating Investigator regarding efficacy will be reported to the NCT Trial Center.

7.5.1.2 Intratumoral IDH1R132H enzyme activity

Noninvasive detection of translational MRS parameters will be performed including 2-HG to evaluate IDH1R132H enzyme activity as described by Choi *et al*. [107].

The method will be established in several clinical sites during the trial. Because of its technical requirements, MRS will not be performed in all clinical sites. As soon as established at a site, the method will be applied and corresponding data will be collected for all patients (with measurable residual disease at visit 2) enrolled by this center. Only patients with baseline MRS data available at visit 2 will be subject for MRS at visits 7, 10, 12, and 13.

The software to conduct the translational MRS sequences will be provided to the clinical sites by the Coordinating Investigator and the Central Neuroradiology. MRS data will be sent to the Central Neuroradiology and the NCT Trial Center / Coordinating Investigator for central assessment.

7.5.2 IDH1R132H-reactive T cell and antibody subtypes

IDH1R132H-specific IgG subtypes will be assessed by the Central Immune Laboratory if IDH1R132H-specific IgG can be detected at any given time point during the study. Measurements will be performed by ELISA based on serum samples collected within the study using SOPs of the Central Immune Laboratory.

IDH1R132H-specific T cell subtypes will be assessed by the Central Immune Laboratory if IDH1R132H-specific T cell responses can be detected at any given time point during the study by ELISpot. Measurements will be performed by intracellular cytokine flow cytometry based on PBMC samples collected at visit 7 using SOPs of the Central Immune Laboratory.

7.5.3 IDH1R132H immunoreactivity in recurrent tumors

IDH1R132H immunoreactivity is analyzed in TIL isolated from recurrent tumors. It will be performed by the Central Immune Laboratory. A detailed description of the method is contained in the SOPs of the Central Immune Laboratory.

7.5.4 HLA Typing

HLA Typing is performed on EDTA blood samples collected within the study. It will be performed by the Central Immune Laboratory. A detailed description of the method is contained in the SOPs of the Central Immune Laboratory.

7.5.5 IDH1R132H DNA in the peripheral circulation

The presence of IDH1R132H DNA in the peripheral circulation is detected on plasma samples collected within the study by the Central Immune Laboratory according to its SOPs.

8 Plan for Treatment or Care after the Trial

After EOS patients will be routinely followed-up usually including visits every 12 weeks and treated regarding standard care according to the discretion of the treating physician.

9 Assessment of Safety

9.1 Specification of Safety Parameters

9.1.1 Adverse Events

According to GCP, an adverse event (AE) is defined as follows: Any untoward medical occurrence in a patient administered a pharmaceutical product and which does not necessarily have a causal relationship with this treatment. An AE can therefore be any unfavourable and unintended sign (including an abnormal laboratory finding), symptom, or disease temporally associated with the use of a medicinal (investigational) product, whether or not related to the medicinal (investigational) product.

An AE may be:

- New symptoms / medical conditions
- New diagnosis
- Changes of laboratory parameters
- Intercurrent diseases and accidents
- Worsening of medical conditions/ diseases existing before clinical trial start
- Recurrence of disease
- Increase of frequency or intensity of episodical diseases.

The following events will not be documented as AEs:

- any hospitalization planned before inclusion in the study (absence of an untoward medical event)
- admission for elective treatments (absence of an untoward medical event)
- admission as a part of the normal planned treatment or monitoring of the studied indication and not associated with any deterioration in condition (absence of an untoward medical event)

A pre-existing disease or symptom will not be considered an AE unless there will be an untoward change in its intensity, frequency or quality. This change will be documented by the investigator.

Surgical procedures themselves are not AEs; they are therapeutic measures for conditions that require surgery. The condition for which the surgery is required may be an AE. Planned surgical measures permitted by the clinical trial protocol and the condition(s) leading to these measures are not AEs, if the condition leading to the measure was present prior to inclusion into the trial.

AEs are classified as ‘non-serious’ or ‘serious’.

9.1.2 Serious Adverse Event

A serious adverse event (SAE) is one that at any dose, regardless of causality or expectedness:

- results in death
- is life-threatening (the term life-threatening refers to an event in which the patient was at risk of death at the time of event and not to an event which hypothetically might have cause death if it was more severe)
- requires patient hospitalization or prolongation of existing hospitalization except for disease-related hospitalization
- results in persistent or significant disability/ incapacity
- results in a congenital anomaly / birth defect or
- is otherwise medically relevant (see below).

The following events will not be graded as ‘serious’ but though will be documented as AEs:

- disease progression and events which are unequivocally related to disease progression regardless of their outcome and regardless whether they otherwise would fulfill seriousness criteria (expected in all subjects and thus not requiring expedited processing).

AEs judged as medically important by the investigator should be treated as SAEs (as per criterion ‘otherwise medically relevant’). Medical and scientific judgment should be exercised in deciding whether expedited reporting is appropriate in other situations, such as important medical events that may not be immediately life-threatening or result in death or hospitalization but may jeopardize the patient’s health and may require intervention to prevent one of the other outcomes listed in the definition above. These AEs should also usually be considered serious.

9.1.3 Expectedness

An ‘unexpected’ adverse event is one the nature or severity of which is not consistent with the applicable product information for the IMPs, e.g. Investigator’s Brochure (IB), Summary of Product Characteristics (SmPC). Furthermore, reports which add significant information on specificity or severity of a known adverse reaction constitute ‘unexpected’ events.

Specific examples would be (a) acute renal failure as an expected adverse reaction with a subsequent new occurrence of interstitial nephritis and (b) hepatitis with a first occurrence of fulminant hepatitis.

9.1.4 Suspected Unexpected Serious Adverse Reaction (SUSAR)

SAEs that are both suspected, i.e. at least possibly related to the IMP and ‘unexpected’ for this IMP, i.e. the nature and/ or severity of which is not consistent with the applicable product information are to be classified as Suspected Unexpected Serious Adverse Reactions (SUSARs).

In case, either the investigator who primary reported the SAE or the second assessor classify the SAE as ‘suspected’ (i.e. not as ‘definitely not related to IMP’) and the SAE is unexpected for the respective IMP it will be categorized as a SUSAR.

All SUSARs are subject to an expedited reporting to the responsible ethics committee(s), the competent higher federal authority (in this study: PEI) and to all participating investigators.

9.2 Period of Observation and Documentation

All AEs reported by the patient or detected by the investigator will be collected during the trial and must be documented on the appropriate pages of the CRF. AEs must also be documented in the patient’s medical records.

In this trial, all AEs that occur after the clinical screening visit (visit 2) will be documented on the pages provided in the CRF. The period of observation ends with the last study visit (EOS). All patients who have AEs, whether considered associated with the use of the trial medication or not, must be monitored to determine the outcome. The clinical course of the AE will be followed up until resolution or normalization of changed laboratory parameters or until it has changed to a stable condition.

9.2.1 Grading of AEs

The grading of AEs in this trial will be carried out on the basis of the 5-grade scale defined in the CTCAE v4.0:

Grade 1: Mild; asymptomatic or mild symptoms; clinical or diagnostic observations only; intervention not indicated.

Grade 2: Moderate; minimal, local or noninvasive intervention indicated; limiting age-appropriate instrumental activity of daily living (ADL)

Grade 3: Severe or medically significant but not immediately life-threatening; hospitalization or prolongation of hospitalization indicated; disabling; limiting self-care ADL.

Grade 4: Life-threatening consequences; urgent intervention indicated.

Grade 5: Death related to AE

The grading of all AEs listed in the CTCAE v4.0 will be based on the information contained therein. The grading of all other AEs, i.e. those which are not listed in the CTCAE v4.0, will be performed by the responsible investigator, based on the following definitions:

mild: temporary event which is tolerated well by the patient.

moderate: event which results in discomfort for the patient and impairs his/ her normal activity.

severe: event which results in substantial impairment of normal activities of patient.

9.2.2 Coherence between AEs and the IMP and Non-IMPs

The investigator will evaluate each AE that occurred after administration of the IMP regarding the coherency with the administration of the IMP (IDH1 peptide vaccine), Non-IMP (TMZ) or immunomodulator (imiquimod) used. The decisive factor in the documentation is the temporal coincidence and relationship between the AE and the (study) drug according to the WHO causality assessment criteria:

certain: A clinical event with a plausible time relationship to the administration of study drug(s) and which cannot be explained by concurrent disease or other drugs or chemicals. To be classified as certain an AE should in addition reasonably comply to the following criteria: 1. response to withdrawal of drug plausible (pharmacologically, pathologically), 2. AE is an objective and specific medical disorder or a recognized pharmacologic phenomenon, 3. recurrence of event on re-exposure to drug (if necessary).

probable: A clinical event with a plausible time relationship to the administration of study drug(s) and which is unlikely to be attributed to concurrent disease or other drugs or chemicals.

possible: A clinical event with a plausible time relationship to the administration of study drug(s), but which could also be explained by concurrent disease or

	other drugs or chemicals.
**unlikely:**	A clinical event whose time relationship to the administration of study drug(s) makes a causal connection improbable, but which could be plausibly explained by underlying disease or other drugs or chemicals.
**unrelated:**	A clinical event with an incompatible time relationship and which could be explained by underlying disease or other drugs or chemicals.
**unclassifiable:**	A clinical event with insufficient information to permit assessment and identification of the cause. However, every possible effort should be undertaken to clarify the suspected cause.

The definitions above are given for the IMP, but also hold true for assessing causal relationships between observed AEs and TMZ or imiquimod.
Events assessed to have possible, probable or certain relationship to the (study) drugs are considered as events with ‘reasonable causal relationship’ for reportability purposes.

9.2.3 Outcome of AEs

The outcome of an AE at the time of the last observation will be classified as:

**Recovered/ resolved**	all signs and symptoms of an AE disappeared without any sequels at the time of the last interrogation.
**Recovering/ resolving**	the intensity of signs and symptoms has been diminishing and/ or their clinical pattern has been changing up to the time of the last interrogation in a way typical for its resolution.
**Not recovered/ not resolved**	signs and symptoms of an AE are mostly unchanged at the time of the last interrogation.
**Recovered/ resolved with sequel**	actual signs and symptoms of an AE disappeared but there are sequels related to the AE.
**Fatal**	resulting in death. If there is more than one adverse event only the adverse event leading to death (possibly related) will be characterized as ‘fatal‘.
**Unknown**	the outcome is unknown or implausible and the information cannot be supplemented or verified.

9.2.4 Action taken with the IMP and the immunomodulator

The action taken with the IMP or the immunomodulator (imiquimod) will be assigned to one of the following categories. A reduction or increase of the IMP or the immunomodulator (imiquimod) dose is not intended in this trial.

**Dose not changed**	no change in the dose of the IMP or imiquimod, respectively
**Temporary discontinuation**	temporary discontinuation of the IMP or imiquimod, respectively
**Drug withdrawn**	discontinuation of the IMP or imiquimod, respectively
**Unknown**	the information is unknown or implausible and it cannot be supplemented or verified
**Not applicable**	the question is implausible (e.g. the patient is dead)

9.2.5 Action taken with the Non-IMP

The action taken with the Non-IMP (TMZ) will be assigned to one of the following categories.

**Dose not changed**	no change in the dose of TMZ
**Dose reduced**	reduction in the dose of TMZ
**Temporary discontinuation**	temporary discontinuation of TMZ
**Dose increased**	increase in the dose of TMZ
**Drug withdrawn**	discontinuation of TMZ
**Unknown**	the information is unknown or implausible and it cannot be supplemented or verified
**Not applicable**	the question is implausible (e.g. the patient is dead)

9.2.6 Countermeasures

The term ‘Countermeasures’ refers to the specific actions taken to treat or alleviate adverse events or to avoid their sequels. Following categories will be used to categorize the countermeasures to adverse events:

**None**	no action taken
**Drug treatment**	newly-prescribed medication or change in dose of a medication
**Others**	other countermeasures, e.g. an operative procedure

9.3 Reporting of Serious Adverse Events by Investigator

All SAEs must be reported by the investigator to the responsible Safety Officer at the KKS Heidelberg within 24 hours after the SAE becomes known using the ‘Serious Adverse Event’ form. The reporting will be performed by faxing of a completed SAE form to the following fax number: ***+49 (0)6221 / 56-33725.***

The initial report must be as complete as possible including details of the current illness and (serious) adverse event and an assessment of the causal relationship between the event and the trial medication (IMPs and Non-IMPs). The investigator must also inform the site monitor in all cases.

Disease progression is not considered a SAE in this trial (for details refer to section 9.1.2).

9.4 Expedited Reporting

SUSARs are to be reported to the ethics committee(s), competent higher regulatory authorities (in this study: PEI) and to all participating investigators within regulative defined timelines, i.e. they are subject to an expedited reporting.

Investigators participating in this trial will report all SAEs to a responsible Safety Officer at the KKS Heidelberg as soon as possible but not later than 24 hours after their notification. The reporting will be performed by faxing of a completed ‘SAE Form’ to the following fax-number: ***+49 (0)6221 / 56-33725***.

9.5 Second Assessment of Serious Adverse Events

All SAE will be subject to a second assessment by a designated person. The designated person for the present trial, referred to as the second assessor is the Coordinating Investigator:

Prof. Dr. Michael Platten

Phone: 06221 / 56-6804
Fax: 06221 / 56-7554
E-Mail: michael.platten@med.uni- heidelberg.de

The second assessor will fill out a 'Second Assessment Form' for each SAE and send it back per fax to the responsible person at the KKS Heidelberg within 48 hours, fax-number: ***+49 (0)6221 / 56-33725***.

The 'Second Assessment Form' will contain the following information:

I) assessment of relationship between SAE and IMPs/Non-IMPs

II) assessment of expectedness of SAE (derived from IB or SmPC)

III) statement if the benefit / risk assessment for the trial did change as a result of SAE.

In case, the Coordinating Investigator himself is the first reporter, he will only assess the items II) and III) during second assessment.

The expedited reporting will be carried out by a responsible person at the KKS Heidelberg.

9.6 Emergency Unblinding

Not applicable. This is an open-label trial.

9.7 Emergency Treatment

During and following a patient's participation in the trial, the investigator should ensure that adequate medical care is provided to a patient for any AE including clinically significant laboratory values. The investigator should inform a patient when medical care is needed for intercurrent illness(es) of which the investigator becomes aware.

Precautions with the vaccination and emergency treatment are described in section 6.5.

10 Statistical Considerations

10.1 Study Design

Study design is described in section 3.

10.2 Primary Objectives and Endpoints

The primary objective is to assess the safety, tolerability and immunogenicity of repeated fixed dose vaccinations with the IDH1 peptide vaccine administered with imiquimod (Aldara®) in patients with IDH1R132H-mutated gliomas of grade III and IV.

10.2.1 Regime Limiting Toxicity (RLT)

Primary safety endpoint is the Regime-Limiting Toxicity (RLT) as defined in section 2.1. Patients suffering a RLT will be removed from trial treatment. The incidence and severity of AEs associated with the treatment regime will be assessed, with an early stopping rule based on the frequency of RLT (refer to section 10.12.1).

10.2.2 Maximum Tolerated Dose (MTD)

Not applicable. Dose escalation of the trial agents is not allowed.

10.2.3 Evaluation of Immunogenicity

Determination of the immunogenicity endpoint will be based on the assessment of the patient's IDH1R132H-specific T-cell and antibody response. Immunogenic response is

evaluated as a dichotomous variable defined as an IDH1R132H-specific T-cell and/or antibody response at any time during the trial measured by IFN-γ ELISpot and peptide-coated ELISA, respectively.

Cutoffs for IDH1R132H-specific T-cell and antibody positivity were evaluated using sera of patients with IDH1R132H-mutated or wild-type glioma and healthy donors [55]: For IFN-γ ELISpot a cut-off of 50 IFN-γ spots after substraction of baseline (MOG) was defined by stimulating PBMC with IDH1 p123-142 R132H or wild-type. For peptide-coated ELISA analyzing IDH1R132H-specific IgG in serum, the cut-off for the optical density related to MOG control was defined to be 5.

Dichotomous immunogenic response is defined as follows: For patients without IDH1R132H-specific T-cell and/or antibody positivity at visit 3 prior to the first vaccination (i.e. without spontaneous immune response), response is defined as IDH1R132H-specific T-cell and/or antibody positivity at any of the visits 5, 7, 10, 12, and 13.

For patients with spontaneous immune response, i.e. IDH1R132H-specific T-cell and/or antibody positivity at visit 3 prior to the first vaccination, response is defined as an at least 3-fold increase of the IDH1R132H-specific T-cell and/or antibody value at any of the visits 5, 7, 10, 12, and 13 compared to the baseline value (visit 3).

10.3 Secondary Objectives and Endpoints

The secondary objectives of this study are to seek evidence of treatment response by assessing:

- the IDH1R132H-specific T-cell response as number of T-cells detected by IFN-γ ELIspot at visits 3, 5, 7, 10, 12, and 13,
- the IDH1R132H-specific antibody response as optical density detected by peptide-coated ELISA at visits 3, 5, 7, 10, 12, and 13,

to evaluate clinical outcome by assessing

- ORR, defined as the proportion of patients showing complete response (CR), partial response (PR) or stable disease (SD) at EOS compared to the baseline value (MRI at visit 2 for ORR under trial drug; MRI prior to any genotoxic therapy for ORR of the complete therapy regime). ORR analysis will be based on the central disease assessment according to the RANO criteria (refer to section 7.5.1),
- PFS, defined as time from the day of first diagnosis to the day of local tumor progression or the day of death of any cause (whichever occurs first), censored by the end of the observation. PFS analysis will be based on the central disease assessment (refer to section 7.5.1). Patients lacking an evaluation of tumor response (based on radiological or clinical assessment) will have their PFS time censored on the date of first diagnosis with duration of 1 day.

  The end of observation is defined as the date of study termination as indicated by the corresponding entry of the CRF. If this date is not documented, the end of observation is defined as the last documented date.

and to evaluate

- association between immunogenicity and the clinical outcome parameters (ORR, PFS).

10.4 Translational Research

In addition to the primary and secondary objectives, a translational research program will

- MRS parameters including R-2-hydroxyglutarate (2-HG) for detection of intratumoral IDH1R132H enzyme activity (only if the patient has measurable residual disease, if local

neuroradiology has implemented the method, and if baseline MRS data are available for visit 2)

- characterize the IDH1R132H-reactive T cell and antibody subtypes
- relate immunogenicity to the HLA type
- relate immunogenicity and clinical outcome to the presence of IDH1R132H DNA in the peripheral circulation
- analyze IDH1R132H immunoreactivity in recurrent tumors, if reoperation or biopsy is clinically indicated and tissue is available, and if local laboratory has implemented a protocol for sample processing
- assess IDH1R132H mutation status in recurrent tumors, if reoperation or biopsy is clinically indicated and tissue is available

10.5 Sample Size Estimation

For this early, non-controlled study it is difficult to define hard criteria for required patient numbers. Sample size estimation is primarily based on the accuracy requirements for the primary endpoint immune response (responder rate) to the IDH1 peptide vaccine administered with imiquimod. In a second step it has been verified that the intended patient number also fulfills the accuracy requirements for the assessment of the RLT.

10.5.1 Sample Size Estimation based on Immunogenicity

The minimum number of patients is primarily based on the ‘proof-of-principle’ endpoint ‘immune response’. For meaningful information about the pharmacodynamics of the IDH1 peptide vaccine, immunological analyses will have to be performed for blood (PBMC and serum) samples collected at various time points pre- and post-vaccination (visits 3, 5, 7, 10, 12, and 13). Thus, patients have to receive a minimum of vaccinations and have to have a minimum number of blood samples collected to be eligible for this analysis.

30 evaluable patients shall be enrolled into the trial. For the definition of ‘evaluable’ refer to section 5.1. Sample size will be adjusted for non-evaluable patients, except for patients leaving the study early due to RLT. All evaluable patients will be included in the analysis of the immune response.

Even though a patient shall be considered evaluable for immune response analysis if he/she has completed the study up to and including visit 7 with 4 vaccinations and all intended blood samples collected at this time point (refer to section 5.1), a more robust estimation of immunogenicity should be based on all later blood samples taken in this study as well (visits 10, 12, and 13). Later blood samples may become non-available or non-evaluable due to patient drop-out. Additionally, patients are reported to be lymphopenic after radiochemotherapy[49,108,109], thus immunomonitoring may not be possible for all time points in all patients. Based on published data [5] we conservatively estimate that approx. 21/30 patients (70%) will be completely evaluable for all time points. These numbers will allow for a first meaningful assessment of biological activity.

The dropout rate is expected to be 20%. Dropouts may arise from disease progression, which is estimated to be around 10% through visit 7, or from reasons described in section 5.4.1. Progression in this study will be routinely assessed by routine MRI scan at visit 7. Expecting a drop-out rate of 20%, ***39 patients will have to be recruited for this study.*** The patients likely to be on trial are generally well-informed and very motivated to comply with study procedures.

The rate of screening failures for inclusion into this study is expected to be high. Patients with newly diagnosed IDH1R132H-mutated glioma will be eligible for screening. Of these approx. 50% of patients cannot be included as they display co-deletion of chromosomes 1p/19q. Further 5% of patients cannot be included as they retain ATRX expression. These potential molecular

screening failures are addressed by early molecular analyses on primary tumor tissue (molecular screening, visit 1) before further TRPs are performed. Based on these data it is expected that ***87 patients will have to be molecularly screened***.

In order to obtain a very rough estimate about potential efficacy, the results of the EGFRvIII peptide vaccine trial in patients with EGFRvIII+ glioblastoma can be taken into account (for details refer to section 1.1.6). Here, a single peptide vaccine targeting a tumor neoantigen (Rindopepimut®, CDX-110) was used in patients with astrocytic brain tumors comparable to the approach used in this trial [65]. Eight of the 14 patients (57%) evaluable for immune response showed a humoral or cellular immune response to the vaccine, which was significantly related to a clinical benefit defined by OS (p=0.043 for humoral response and p=0.03 for cellular immune response).

The analyses demonstrated that 6 out of 25 patients with IDH1R132H-mutated gliomas (24%) had constitutive cellular or humoral immune responses to IDH1R132H [unpublished data] compared with 0 of 17 patients in the EGFRvIII phase II vaccine trial, suggesting a more profound immunogenicity of IDH1R132H. Based on these data a responder rate of 60% (comparable to EGFRvIII) is reasonable to be expected compared with an assumed 24% of baseline responders. This would allow the conclusion that the IDH1 peptide vaccine is immunogenic in this patient population.

Altogether, we regard the suggested patient number of 39 to be recruited and 21 to be completely evaluable as a reasonable minimum required for making decisions for the next stage of development.

10.5.2 Sample Size Estimation based on Safety

The sample size estimation also fulfills the accuracy requirements for the RLT rate: Based on previously published experience with peptide vaccines (refer to Sections 1.1.6 and 6.3), we expect that unacceptable rate of RLT will be reached, if ≥ 15% (95% CI 6% to 31%) of 39 patients suffer from RLT.

10.6 Patient Allocation

In each clinical site all patients who enter into the screening period for the trial will receive a unique screening ID before any TRP is performed. The screening ID will be assigned by the clinical site using the trial site ID (assigned at trial start by the NCT Trial Center) plus a consecutive number. This number will be used to identify the patient throughout the clinical trial and must be used on all trial documentation related to the patient. Clinical sites must complete the appropriate pages of the CRF for all patients screened and send the Molecular Screening Fax, the Clinical Screening Fax and the Inclusion Fax to the NCT Trial Center, even if the patient is not treated in this study. Patients who fulfill the enrollment criteria will be enrolled. For these patients the data management of the NCT Trial Center will assign a unique patient ID, which will be sent to the clinical site.

10.7 Patient Evaluability, Patient Replacement and Analysis Populations

Patients who were screened, but not included into the study will be listed only. This means their demographic data (Screening ID, gender, age) and the reason for not inclusion into the study will be listed, but will not be included in any statistical analysis.

All patients receiving any amount of the planned study treatment will be included in the safety analysis. In the analysis of immunogenicity all evaluable patients will be included (for definition of ‘evaluable’ refer to section 5.1). Non-evaluable patients will be replaced for assessment of immunogenicity, except for patients leaving the study early due to RLT.

10.8 Statistical Analysis

The statistical analysis will be carried out by the responsible biostatistician at the NCT Trial Center Heidelberg using the SAS statistical software [SAS Institute Inc., Cary, NC, USA]. The analysis will be done as soon as the database has been declared to be complete and accurate, and has been locked. Detailed description of the planned analysis and reporting will be defined in the statistical analysis plan, which has to be authorized by the biometrician and the CI before opening the database.

10.8.1 Primary Analyses

Summary tables will present the number of patients observed with immune responses, the corresponding percentages and exact 95% CIs according to Pearson-Clopper.
Summary tables will present the number of patients observed with RLTs, the corresponding percentages and exact 95% CIs according to Pearson-Clopper.

The analysis will not be stratified by center or adjusted for center effects. However, for descriptive purposes additional results will be presented for each center separately. The statistical analysis generally consists of summary statistics and interval estimation.

10.8.2 Analysis of the Secondary Endpoints

All secondary variables will be analyzed using explorative and mainly descriptive methods. Continuous variables will be summarized using standard summary statistics as appropriate. Summary statistics for categorical variables will include frequency counts and percentages. If appropriate, graphical presentations of data will be created. Appropriate confidence intervals of effect-estimates will be given to quantify the degree of uncertainty.

All statistical tests will be two-tailed at the significance level of 5%. Given the low number of patients and the multiplicity of the secondary analyses all statistical tests are of a strictly exploratory nature.

10.8.3 Demographic and other Baseline Characteristics

Categorical baseline characteristics, like gender, age, medical history, performance status and MMSE, concomitant diseases, tumor grading according to WHO criteria [1], and concomitant treatment maintained, will be summarized by frequency tables. Summary statistics will be provided for quantitative variables like age, weight, and temperature.

10.8.4 Safety Data Analysis

The assessment of safety will be mainly based on the frequency of adverse events (see Section 9) and on the number of laboratory values (see section 7.3.4, 7.3.5, and 7.3.6) that fall outside of pre-determined ranges and/or show prominent worsening from baseline.

Frequencies of patients experiencing at least one AE will be displayed. Detailed information collected for each AE will include: A description of the event, the duration, whether the AE was serious, the intensity, the relationship to the study drug, actions taken, and the clinical outcome. Summaries of incidence rates (frequencies and percentages) of AEs by MedDRA System Organ Class (SOC) and Preferred Term will be prepared. Such summaries will be displayed for all AEs, AEs by intensity, and AEs by the relationship to the study drug. Summary tables will present the number of patients observed with AEs and the corresponding percentages. Furthermore, the most common AEs (those occurring in at least 10% of the treatment group) will be determined.
Laboratory data will be summarized by presenting shift tables using normal ranges (baseline to most extreme post-baseline value) and by presenting summary statistics of raw data and change from baseline values (means, medians, standard deviations, ranges).
All proportions will be given along with exact Pearson-Clopper 95% confidence bounds.

10.8.5 Clinical Response Rate and Survival Analyses

ORR: Summary tables will present the number of patients observed ORR, the corresponding percentages and exact 95% CIs according to Pearson-Clopper.

The PFS endpoint will be calculated using the Kaplan-Meier method. The Kaplan-Meier estimate will be used to compute the proportion surviving with the 95% CI, calculated using Greenwood's formula.

Logistic regression models will be used to explore the relationship between the relative changes in the immunologic parameters and clinical response and, in particular, whether any confounder for the relationship between immune response and clinical response can be determined.

Time-independent Proportional Hazards models will be used to analyze the prognostic influence of relative changes in the immunologic parameters under IDH1R132H administration, for the PFS. In addition, models containing dichotomized changes in the parameters as a predictor variable will be built and multivariable modeling will be used to assess these predictors in a more comprehensive way.

10.8.6 Other Analyses

Patient disposition (the number of patients enrolled, treated, and in the analysis populations) will be tabulated. In addition, the number of patients who withdrew from the study and reasons for discontinuation will be summarized. Physical examinations, prior and concomitant medications, and vital signs will be recorded by visit. ECG will be summarized using descriptive statistics for each visit and for changes from baseline. Study drug administration and reasons for the deviations from the planned therapy will be tabulated. Summary tables will be prepared to examine the distribution of laboratory measures over time.

10.8.7 Translational Research Program

All variables of translational research will be analyzed using explorative and mainly descriptive methods. Continuous variables will be summarized using standard summary statistics as appropriate. Summary statistics for categorical variables will include frequency counts and percentages. If appropriate, graphical presentations of data will be created. Appropriate confidence intervals of effect-estimates will be given to quantify the degree of uncertainty.

For each immunological parameter the change under the vaccination [value measured after administration of IDH1R132H *vs*. baseline value (= last available value prior of IDH1R132H administration, visit 2)] and the relative changes [($variable_{post}$-$variable_{pre}$)/$variable_{pre}$] will be analyzed.

10.9 Handling of Missing Values

Missing values will not be replaced or imputed. For patients with an incomplete follow-up, the time to the last follow-up date will be used as the censoring time in the analysis of time-to-event data.

10.10 Interim Analysis

Interim analyses will be performed for safety reasons to detect unexpected shortening of PFS and thus, allows for early trial termination. As soon as data regarding 12-months PFS for the first 10 (and if applicable, 20 and 30) patients are available, interim analyses will be performed. This will be approximately 9 months after the 10th (20th, 30th) patient will be enrolled into the trial. For details regarding the interim analysis refer to section 10.12.2.

10.11 Multiple Comparisons/ Multiplicity

No adjustment for multiplicity will be done. All statistical tests are of an explorative nature.

10.12 Early Trial Termination, Continuous Monitoring of Toxicity and Stopping Rules

10.12.1 Early trial termination due to toxicity

An early trial termination will be taken into consideration if the risks or the toxicity under the study medication are unjustifiable.

A continuous monitoring will be performed to determine whether or not the enrollment must be stopped due to an unacceptable rate of RLT.
To calculate the posterior distribution of the RLT rate r, the binomial-beta model is used with a non-informative prior. Thus, if y cases of RLT have been observed among the first k patients the posterior distribution of r is beta(1+y,1+k-y). The formal stopping criterion is reached if the probability (Pr) that the true RLT rate r exceeds the unacceptable RLT of 15% is higher than 95%; formally:

(1) $\Pr(r>15\%) \geq 95\%$

This probability is based on the posterior distribution of r and is calculated continuously up to the kth patient. The critical boundaries c(k) for the minimum observed numbers of RLT among the first k patients can be calculated (refer to Table 2) such that inequality (1) is satisfied.

Table 2: Critical boundaries to stop the recruitment due to an inacceptable RLT [110]

Enrollment of patients	critical boundaries Patients with RLT	Probability of interruption of the trial pstop* (%)			
**m**	**c(k)**	**r=15%**	**r=23%**	**r=30%**	**R=35%**
1	-	-	-	-	-
2	2	2.16	5.18	8.97	12.18
3	2	5.96	13.23	21.41	28.03
4	2	10.83	22.48	34.57	43.45
5	2	16.31	32.20	46.88	57.05
6	3	16.31	32.20	46.88	57.05
7	3	17.22	34.38	50.20	60.90
8	3	18.87	38.00	55.26	66.42
9	3	21.17	42.59	61.07	72.26
10	4	21.17	42.59	61.07	72.26
11	4	21.59	43.85	62.82	74.14
12	4	22.44	45.93	65.68	77.07
13	4	23.68	48.81	69.12	80.35
14	5	23.68	48.81	69.12	80.35
15	5	23.91	49.56	70.21	81.55
16	5	24.41	50.97	72.10	83.37
17	5	25.20	52.92	74.46	85.45
18	6	25.20	52.92	74.46	85.45
19	6	25.35	53.48	75.29	86.22
20	6	25.72	54.52	76.66	87.39
21	6	26.22	55.96	78.37	88.80
22	6	26.90	57.85	80.33	90.32
23	7	26.90	57.85	80.33	90.32

24	7	27.03	58.39	80.96	90.84
25	7	27.29	59.23	81.99	91.67
26	7	27.72	60.48	83.31	92.66
27	7	28.29	62.13	84.82	93.68
28	8	28.29	62.13	84.82	93.68
29	8	28.41	62.56	85.29	94.03
30	8	28.61	63.30	86.11	94.55
31	8	28.96	64.41	87.11	95.16
32	8	29.43	65.68	88.22	95.80
33	9	29.43	65.68	88.22	95.80
34	9	29.51	66.02	88.55	96.01
35	9	29.70	66.72	89.14	96.34
36	9	29.99	67.62	89.86	96.75
37	9	30.37	68.73	90.71	97.16
38	10	30.37	68.73	90.71	97.16
39	10	30.46	69.92	90.98	97.31

***:p_{stop}:** probability of interruption of the trial due to RLT up to this patient

If at any point of the enrollment the posterior probability that the true proportion of RLT is unacceptable, the enrollment will be interrupted. The posterior probability will be evaluated continuously based on the observed toxicity up to the kth patient (k = 2,...,39). With the specification given above, the total probability to stop the recruitment can be calculated by means of computer simulations for different assumptions of the true RLT rate (refer to Table 2). The probability to interrupt the trial due to RLT is quite low if the true probability of toxicity is small but could rise high with increasing probability of the true toxicity.

Example:

4th patient: The probability to interrupt the trial due to RLT up to this patient is 10.83% (22.48% /34.57% /43.45) if the true RLT rate r equals 15% (23%, 30% or 35%, respectively).

In case of fulfilling the stopping criteria, the recruitment have to be stopped and the DMC is to be informed immediately. The DMC has to provide advice to the Coordinating Investigator concerning the continuation or discontinuation of recruitment and/or treatment for the whole trial or of one of the treatment groups.

10.12.2 Early trial termination due to shortening of PFS

While the focus of this study is on safety and tolerability, it was deemed important to ensure that the therapy does not negatively affect the course of the disease. To this aim, interim analyses with endpoint 12-months PFS (for PFS definition refer to section 10.3, MRI prior to any genotoxic therapy is used as baseline MRI for PFS monitoring) will be carried out.

These interim analyses are to provide the basis for notifying the DMC for a recommendation on whether or not to continue the study in light of the accumulated PFS data; they are not based on a formal statistical test but rather on an observed effect.

The effect leading to a decision over this issue will be an observed decrease of at least 10 percentage points (Trigger = 10, Tables 3, 4 and 5) in the estimated 12-months PFS rate compared to the anticipated value (derived from previous studies) of 70.7%, corresponding to a median PFS of 24 months if exponential distribution of the PFS is assumed.

In order to determine the probabilities of consulting the DMC (which may be viewed as upper bounds for the probabilities of stopping the trial because of poor PFS data) simulation programs

(R code) were written. PFS was assumed to follow an exponential distribution; patient accrual was modeled to be uniformly distributed in the interval [0, 24months] or – alternatively – to follow a homogeneous Poisson process with rate 39/24months. The true median PFS used for determining the exponential parameter of the simulated samples was assumed to range from 12 to 36 months. The 12-months PFS rate of the simulated studies was calculated using the Kaplan-Meier estimator.

Interim analyses were assumed to be performed only on the condition that not all 39 study patients have reached a follow-up of 12 months prior to the analysis (for otherwise any decision to stop the accrual or treatment of study patients would be futile). Also, the probabilities of notifying the DMC at the second interim analysis were calculated alternatively on the assumption that the decision of the DMC regarding continuation of the study if and when consulted at the first interim analysis is invariably negative (Table 4) and positive (Table 5), respectively, thus delimiting the extreme cases.

Table 3 shows the probabilities of consulting the DMC at the first interim analysis; and Tables 4 and 5 show the probabilities of consulting the DMC at the second interim analysis (100,000 simulation runs for each scenario, assuming uniform patient entry):

Table 3: Probability (%) of notifying the DMC at the first interim analysis, n.interim = 10*

Probabilities for the anticipated 12-months PFS rate of 70.7% and a decrease of the 12-months PFS of at least 10 percentage points compared to the anticipated value (Trigger = 10) are shown in bold.

* The interim analyses are to be carried out once n.interim patients have reached either 12 months of follow-up or an event, whichever comes first. To avoid overrun issues, data available from patients enrolled between the recruitment of the n.interim-th patient and the interim analysis will be included.

Median PFS (months)	12-months PFS rate (%)	Trigger				
		5	10	15	20	25
**12**	50.0	90.0	**80.9**	66.7	51.6	36.9
**15**	57.4	74.2	**58.9**	44.5	28.9	17.6
**18**	63.0	57.3	**42.2**	27.4	16.1	8.1
**21**	67.3	42.5	**26.9**	16.2	8.3	4.0
**24**	**70.7**	**32.0**	**18.1**	**9.4**	**4.7**	**1.9**
**27**	73.5	22.6	**12.5**	5.9	3.0	1.0
**30**	75.8	16.9	**8.4**	3.7	1.6	0.6
**33**	77.7	12.4	**6.0**	2.5	1.0	0.4
**36**	79.4	9.2	**4.1**	1.7	0.6	0.2

Table 4: Probability (%) of notifying the DMC at the second interim analysis, n.interim = 20* (assuming trial stop if the DMC is consulted at the first interim analysis)

Probabilities for the anticipated 12-months PFS rate of 70.7% and a decrease of the 12-months PFS of at least 10 percentage points compared to the anticipated value (Trigger = 10) are shown in bold.

* The interim analyses are to be carried out once n.interim patients have reached either 12 months of follow-up or an event, whichever comes first. To avoid overrun issues, data available from patients enrolled between the recruitment of the n.interim-th patient and the interim analysis will be included.

Median PFS (months)	12-months PFS rate (%)	Trigger				
		5	10	15	20	25
**12**	50.0	6.6	**9.8**	12.3	11.7	8.2

**15**	57.4	11.5	**12.8**	10.2	6.1	3.6
**18**	63.0	11.5	**9.6**	6.2	3.0	1.2
**21**	67.3	9.6	**6.7**	3.3	1.1	0.5
**24**	**70.7**	**6.9**	**3.9**	**1.6**	**0.5**	**0.1**
**27**	73.5	5.1	**2.1**	0.7	0.2	0.1
**30**	75.8	3.5	**1.1**	0.4	0.2	0.0
**33**	77.7	2.3	**0.7**	0.2	0.0	0.0
**36**	79.4	1.5	**0.4**	0.2	0.0	0.0

Table 5: Probability (%) of notifying the DMC at the second interim analysis, n.interim = 20* (assuming trial continuation if the DMC is consulted at the first interim analysis)

Probabilities for the anticipated 12-months PFS rate of 70.7% and a decrease of the 12-months PFS of at least 10 percentage points compared to the anticipated value (Trigger = 10) are shown in bold.

* The interim analyses are to be carried out once n.interim patients have reached either 12 months of follow-up or an event, whichever comes first. To avoid overrun issues, data available from patients enrolled between the recruitment of the n.interim-th patient and the interim analysis will be included.

Median PFS (months)	12-months PFS rate (%)	Trigger				
		5	10	15	20	25
**12**	50.0	94.6	**86.0**	71.8	52.2	33.9
**15**	57.4	80.0	**62.7**	42.7	24.4	11.3
**18**	63.0	59.9	**38.9**	21.6	9.7	3.6
**21**	67.3	42.4	**23.2**	10.4	3.8	0.9
**24**	**70.7**	**28.7**	**12.6**	**4.7**	**1.5**	**0.4**
**27**	73.5	17.6	**7.3**	2.4	0.6	0.1
**30**	75.8	11.3	**3.9**	1.1	0.3	0.0
**33**	77.7	7.6	**2.6**	0.4	0.1	0.0
**36**	79.4	4.8	**1.3**	0.3	0.0	0.0

In case shortening of PFS is detected in the interim analyses, the DMC will be immediately contacted to provide advice to the Coordinating Investigator concerning the continuation or discontinuation of the trial.

11 Data Management

11.1 Data Collection

As used in this protocol, the term Case Report Form (CRF) should be understood to refer a paper form. The method of data collection is described in section 7.2.1.

11.2 Data Handling

After receipt of the CRF-pages at the data management of the NCT Trial Center, all data will be entered in a database as recorded in the CRF. In order to ensure that the database reproduces the CRFs correctly, the NCT Trial Center accomplishes a double entry of data. The completeness, validity and plausibility of data are examined by validated programs, which thereby generate queries.

All missing data or inconsistencies will be reported back to the site(s) and clarified by the responsible investigator. If no further corrections are to be made in the database it will be declared closed and used for statistical analysis.

All data management activities will be done according to the current SOPs of the NCT Trial Center and as outlined in the trial-specific Data Management Plan and Monitoring Plan.

11.3 Storage and Archiving of Data

According to the §13 of the German GCP Regulation (GCP-V) all important trial documents (e.g. CRF) will be archived for at least 10 years after the trial termination.

The Coordinating Investigator will archive the Trial Master File (TMF) including protocol, CRFs, report etc. according to section 5.6 of the ICH Consolidated Guideline on GCP (E6) and to local law or regulations.

The investigator(s) will archive all trial data (source data and Investigator Site File (ISF) including patient identification list and relevant correspondence) according to section 4.9 of the ICH Consolidated Guideline on GCP (E6) and to local law or regulations. The Patient Identification List will be archived for at least 15 years after the trial termination.

If the investigator relocates, retires, or for any reason withdraws from the study, the NCT Trial Center and the Coordinating Investigator should be prospectively notified. The study records must be transferred to an acceptable designee, such as another investigator, another institution, or the NCT Trial Center. The investigator must obtain written permission of the NCT Trial Center and the Coordinating Investigator before disposing of any records, even if archiving requirements have been met.

12 Ethical and Legal Aspects

12.1 Good Clinical Practice

The procedures set out in this trial protocol, pertaining to the conduct, evaluation, and documentation of this trial, are designed to ensure that all persons involved in the trial abide by Good Clinical Practice (GCP) and the ethical principles described in the applicable version of the Declaration of Helsinki. The trial will be carried out in keeping with local legal and regulatory requirements.

12.2 Patient Information and Informed Consent

Before being admitted to the molecular screening or the remaining trial starting from the clinical screening to the EOS visit, the patient must consent to participate after the nature, scope, and possible consequences of the molecular screening or the remaining trial, respectively, have been explained in a form understandable to him or her.

Two informed consent forms will be used in this study: a short one covering the molecular screening (*short IC*) and an extended one covering the remaining trial (*extended IC*). The patient must give consent in writing on the short and the extended informed consent form for the molecular screening and the remaining trial, respectively. The personally signed and dated Informed Consent Forms must be kept on file by the investigator(s), and documented in the CRF.

If a patient is not able to understand the nature, scope, and possible consequences of the clinical trial, he/she will not be included in the trial.

If the patient is able to understand the nature, scope, and possible consequences of the clinical trial, but he/she is not able to write (e.g., if patient suffers from agraphia), a witness must be present when the trial is explained to the patient and when the patient gives his/her consent to

participate in the trial as required by § 40 (1) AMG. The oral consent of the patient has to be documented on the informed consent form(s), and has to be dated and signed by the witness. The witness must not be an employee of the clinical site and/or a member of the study team.

A copy of the signed informed consent document(s) must be given to the patient. The documents must be in a language easily understandable to the patient and must specify who informed the patient.

If new safety information results in significant changes in the risk/benefit assessment, the consent form should be reviewed and updated if necessary. All patients (including those already being treated) should be informed of the new information and must give their written informed consent to continue the study.

12.3 Confidentiality

The data obtained in the course of the trial will be treated pursuant to the Federal Data Protection Law (Bundesdatenschutz- bzw. Landesdatenschutzgesetz, BDSG, LDSG) as well as to § 40 (2a) AMG.

During the clinical trial, patients will be identified solely by individual identification codes (Screening ID, Patient ID). Trial findings stored on a computer will be stored in accordance with local data protection law and will be handled in strictest confidence. For protection of these data, organizational procedures are implemented to prevent distribution of data to unauthorized persons. The appropriate regulations of local data legislation will be fulfilled in its entirety.

The patient consents in writing (in case the patient is not able to write a witness must be involved; for details refer to section 12.2) to relieve the investigator from his/her professional discretion in so far as to allow inspection of original data for monitoring purposes by health authorities and authorized persons (inspectors, monitors, auditors). Authorized persons (clinical monitors, auditors, and inspectors) may inspect the patient-related data collected during the trial ensuring the data protection law.

The investigator will maintain a patient identification list (patient IDs with the corresponding patient names) to enable records to be identified.

Patients who did not consent to circulate their pseudonymized data will not be included into the trial.

12.4 Responsibilities of Investigator

The investigator(s) should ensure that all persons assisting with the trial are adequately informed about the protocol, any amendments to the protocol, the trial treatments, and their trial-related duties and functions.

The investigator(s) should maintain a list of study physicians and other appropriately qualified persons to whom he or she has delegated significant trial-related duties.

The investigator(s) should support monitoring, auditing and inspections as described in sections 13.1 and 13.2.

12.5 Approval of Trial Protocol and Amendments

Before the start of the trial, the trial protocol, informed consent document, and any other appropriate documents will be submitted to the independent Ethics Committee (EC) as well as to the competent federal authority (in this study: PEI). A written favourable vote of the EC and an (implicit) approval by the competent higher federal authority are a prerequisite for initiation of this clinical trial. The statement of EC should contain the title of the trial, the trial code, the trial site, and a list of reviewed documents. It must mention the date on which the decision was made and must be officially signed by a committee member.

Before the first patient is enrolled in the trial, all ethical and legal requirements must be met. All planned substantial changes (see §10, (1) of the German GCP-Regulation, GCP-V) will be submitted to EC and the competent higher federal authority in writing as protocol amendments. They have to be approved by the EC and the competent higher federal authority.

The Coordinating Investigator or the NCT Trial Center, and if applicable the investigator(s), will keep a record of all communication with the EC and the competent authorities.

12.6 Continuous Information to Independent Ethics Committee

Pursuant to the German Drug Law (AMG) and the GCP Regulation, the EC and the competent higher federal authority will be informed of all suspected serious unexpected adverse reactions (SUSARs) and all AEs resulting in death or being live-threatening occurring during the trial. Both institutions will be informed in case the risk-benefit assessment did change or any others new and significant hazards for patients' safety or welfare did occur. Furthermore, a report on all observed serious adverse events (SAEs) will be submitted once a year – Development Safety Update Report (DSUR).

The EC and the regulatory authorities must be informed of the end of the trial. They will be provided with a summary of trial results within one year after the end of clinical phase (LPLV).

12.7 Notification of Regulatory Authorities

The local regulatory authorities as responsible for each particular investigator and the competent higher federal authority will be informed before the beginning, during and at the end of the trial according to §67 AMG and §13 GCP-V. Each investigator is obliged to notify his / her local regulatory authority and the competent higher federal authority according §67 AMG and §12 (1, 2, 6) GCP-V.

12.8 Registration of the Trial

Prior to the beginning of the clinical phase (FPFV) the Coordinating Investigator will register the trial at a public accessible clinical trial register having the status of a primary register according to the International Clinical Trials Registry Platform (ICTRP) and correspondingly is listed at the International Clinical Trials Registry Platform of the World Health Organization (WHO, http://www.who.int/ictrp/en/). The requirements are fulfilled by the European Clinical Trials Register and submission of EMA Module 1 (Clinical Trial Application Form).

The registration is a prerequisite for a publication in many peer-reviewed journals (see Uniform Requirements for Manuscripts Submitted to Biomedical Journals by the International Committee of Medical Journal Editors; http://www.icmje.org/ publishing_10register.html).

12.9 Insurance

According to §40 AMG, the sponsor has to subscribe to an insurance policy covering, in its terms and provisions, its legal liability for injuries caused to participating persons and arising out of this research performed strictly in accordance with the scientific protocol as well as with applicable law and professional standards. The insurance was taken out at the HDI-Gerling Versicherung AG (insurance number: 57 010310 03018, maximum limit: € 500.000 per participating person).

Any impairment of health which might occur in consequence of trial participation must be notified to the insurance company. The patient is responsible for notification. The insured person will agree with all appropriate measures serving for clarification of the cause and the extent of damage as well as the reduction of damage.

During the conduct of the trial, the patient must not undergo other clinical treatment except for cases of emergency. The patient is bound to inform the investigator immediately about any adverse events and additionally drugs taken. The terms and conditions of the insurance should be delivered to the patient.

The insurance company has to be informed about all amendments that could affect patients' safety.

13 Quality Assurance

13.1 Monitoring

Monitoring will be done by personal visits from a clinical monitor according to SOPs of the KKS Heidelberg. The monitor will review the entries into the CRFs on the basis of source documents. The investigator must allow the monitor to verify all essential documents and must provide support at all times to the monitor.

By frequent communications (letters, telephone, fax), the site monitor will ensure that the trial is conducted according to the protocol and regulatory requirements.

A detailed description of the monitoring process will be provided in a separate clinical monitoring plan.

13.2 Inspections/ Audits

Regulatory authorities and an auditor authorized by the sponsor may request access to all source documents, CRF, and other trial documentation. Direct access to these documents must be guaranteed by the investigator who must provide support at all times for these activities.

14 Agreements

14.1 Financing of the Trial

This is a non-commercial trial. It will be financed by the Neurology Clinic of the University Hospital Heidelberg and the German Cancer Consortium (DKTK).

14.2 Financial Disclosure

Before the start of the trial, each investigator will disclose to the sponsor any proprietary or financial interests he or she might hold in the sponsors / a funding company, in the investigational product(s) or any commercial organisation being involved in the clinical trial. Each investigator also has to confirm that he / she has not entered into any financial arrangement whereby the value of compensation paid could affect the outcome of the clinical trial.

The investigator(s) agree(s) to update this information in case of significant changes.

14.3 Reports

The biostatistician will prepare the final trial report together with the Coordinating Investigator within 12 months after the end of the study (LPLV).

14.4 Publication

All information concerning the trial is confidential before publication.

15 Signatures

The present trial protocol was subject to critical review and has been approved in the present version by the persons undersigned. The information contained is consistent with:

- the current risk-benefit assessment of the investigational medicinal product
- the moral, ethical, and scientific principles governing clinical research as set out the principles of GCP and in the applicable version of the Declaration of Helsinki.

The investigator(s) will be supplied with details of any significant or new finding including relevant safety information relating to treatment with the investigational medicinal product.

Date: n.a.

Signature: Delegated to the Coordinating Investigator by internal IIT contract

Name (block letters): Ruprecht-Karls-University Heidelberg, Medical Faculty
- represented in law by Universitäts-klinikum Heidelberg and its Commercial Director Ms. Irmtraut Gürkan -

Function: **Sponsor**

Date: 31.3.15

Signature:

Name (block letters): Prof. Dr. Michael Platten

Function: **Coordinating Investigator**

Date: 31 MAR 2015

Signature:

Name (block letters): Prof. Dr. Wolfgang Wick

Function: **Co-Investigator**

Date: 31.03.15

Signature:

Name (block letters): Irini Karapanagiotou-Schenkel

Function: **Biostatistician**

16 Declaration of Investigator

I have read the above trial protocol and confirm that it contains all information to conduct the clinical trial. I pledge to conduct the clinical trial according to the protocol.

I will enroll the first patient only after all ethical and regulatory requirements are fulfilled. I pledge to obtain written consent for trial participation from all patients.

I know the requirements for accurate notification of serious adverse events and I pledge to document and notify such events as described in the protocol.

I pledge to retain all trial-related documents and source data as described.

Date:____________________ Signature: ______________________________

Name (block letters): ______________________________

Trial Site (address): ______________________________

____

____

17 References

1 Louis, D. N. *et al.* The 2007 WHO classification of tumours of the central nervous system. *Acta neuropathologica* **114**, 97-109, doi:10.1007/s00401-007-0243-4 (2007).

2 Cairncross, G. *et al.* Phase III trial of chemoradiotherapy for anaplastic oligodendroglioma: long-term results of RTOG 9402. *Journal of clinical oncology : official journal of the American Society of Clinical Oncology* **31**, 337-343, doi:10.1200/jco.2012.43.2674 (2013).

3 Kouwenhoven, M. C. *et al.* Molecular analysis of anaplastic oligodendroglial tumors in a prospective randomized study: A report from EORTC study 26951. *Neuro-oncology* **11**, 737-746, doi:10.1215/15228517-2009-011 (2009).

4 van den Bent, M. J. *et al.* MGMT promoter methylation is prognostic but not predictive for outcome to adjuvant PCV chemotherapy in anaplastic oligodendroglial tumors: a report from EORTC Brain Tumor Group Study 26951. *Journal of clinical oncology : official journal of the American Society of Clinical Oncology* **27**, 5881-5886, doi:10.1200/jco.2009.24.1034 (2009).

5 Wick, W. *et al.* NOA-04 randomized phase III trial of sequential radiochemotherapy of anaplastic glioma with procarbazine, lomustine, and vincristine or temozolomide. *Journal of clinical oncology : official journal of the American Society of Clinical Oncology* **27**, 5874-5880, doi:10.1200/jco.2009.23.6497 (2009).

6 Stummer, W. *et al.* Fluorescence-guided surgery with 5-aminolevulinic acid for resection of malignant glioma: a randomised controlled multicentre phase III trial. *The lancet oncology* **7**, 392-401, doi:10.1016/s1470-2045(06)70665-9 (2006).

7 Cairncross, J. G. *et al.* Specific genetic predictors of chemotherapeutic response and survival in patients with anaplastic oligodendrogliomas. *Journal of the National Cancer Institute* **90**, 1473-1479 (1998).

8 Stupp, R. *et al.* Promising survival for patients with newly diagnosed glioblastoma multiforme treated with concomitant radiation plus temozolomide followed by adjuvant temozolomide. *Journal of clinical oncology : official journal of the American Society of Clinical Oncology* **20**, 1375-1382 (2002).

9 Weller, M., Muller, B., Koch, R., Bamberg, M. & Krauseneck, P. Neuro-Oncology Working Group 01 trial of nimustine plus teniposide versus nimustine plus cytarabine chemotherapy in addition to involved-field radiotherapy in the first-line treatment of malignant glioma. *Journal of clinical oncology : official journal of the American Society of Clinical Oncology* **21**, 3276-3284, doi:10.1200/jco.2003.03.509 (2003).

10 van den Bent, M. J. *et al.* Adjuvant procarbazine, lomustine, and vincristine chemotherapy in newly diagnosed anaplastic oligodendroglioma: long-term follow-up of EORTC brain tumor group study 26951. *Journal of clinical oncology : official journal of the American Society of Clinical Oncology* **31**, 344-350, doi:10.1200/jco.2012.43.2229 (2013).

11 Hartmann, C. *et al.* Patients with IDH1 wild type anaplastic astrocytomas exhibit worse prognosis than IDH1-mutated glioblastomas, and IDH1 mutation status accounts for the unfavorable prognostic effect of higher age: implications for classification of gliomas. *Acta neuropathologica* **120**, 707-718, doi:10.1007/s00401-010-0781-z (2010).

12 Wick, W. *et al.* Prognostic or predictive value of MGMT promoter methylation in gliomas depends on IDH1 mutation. *Neurology* **81**, 1515-1522, doi:10.1212/WNL.0b013e3182a95680 (2013).

13 Laperriere, N., Zuraw, L. & Cairncross, G. Radiotherapy for newly diagnosed malignant glioma in adults: a systematic review. *Radiotherapy and oncology : journal of the European Society for Therapeutic Radiology and Oncology* **64**, 259-273 (2002).

14 Glioma_Meta-Analysis_Trialists_(GMT)_Group. Chemotherapy for high-grade glioma. *The Cochrane database of systematic reviews*, Cd003913, doi:10.1002/14651858.cd003913 (2002).

15 Yung, W. K. *et al.* Multicenter phase II trial of temozolomide in patients with anaplastic astrocytoma or anaplastic oligoastrocytoma at first relapse. Temodal Brain Tumor Group. *Journal of clinical oncology : official journal of the American Society of Clinical Oncology* **17**, 2762-2771 (1999).

16 Yung, W. K. *et al.* A phase II study of temozolomide vs. procarbazine in patients with glioblastoma multiforme at first relapse. *British journal of cancer* **83**, 588-593, doi:10.1054/bjoc.2000.1316 (2000).

17 Stupp, R. *et al.* Radiotherapy plus concomitant and adjuvant temozolomide for glioblastoma. *The New England journal of medicine* **352**, 987-996, doi:10.1056/NEJMoa043330 (2005).

18 Hegi, M. E. *et al.* MGMT gene silencing and benefit from temozolomide in glioblastoma. *The New England journal of medicine* **352**, 997-1003, doi:10.1056/NEJMoa043331 (2005).

19 Wick, W. *et al.* MGMT testing--the challenges for biomarker-based glioma treatment. *Nature reviews. Neurology* **10**, 372-385, doi:10.1038/nrneurol.2014.100 (2014).

20 Esteller, M. *et al.* Inactivation of the DNA-repair gene MGMT and the clinical response of gliomas to alkylating agents. *The New England journal of medicine* **343**, 1350-1354, doi:10.1056/nejm200011093431901 (2000).

21 Silber, J. R. *et al.* O6-methylguanine-DNA methyltransferase-deficient phenotype in human gliomas: frequency and time to tumor progression after alkylating agent-based chemotherapy. *Clinical cancer research : an official journal of the American Association for Cancer Research* **5**, 807-814 (1999).

22 Quinn, J. A. *et al.* Phase II trial of carmustine plus O(6)-benzylguanine for patients with nitrosourea-resistant recurrent or progressive malignant glioma. *Journal of clinical oncology : official journal of the American Society of Clinical Oncology* **20**, 2277-2283 (2002).

23 Balss, J. *et al.* Analysis of the IDH1 codon 132 mutation in brain tumors. *Acta neuropathologica* **116**, 597-602, doi:10.1007/s00401-008-0455-2 (2008).

24 Parsons, D. W. *et al.* An integrated genomic analysis of human glioblastoma multiforme. *Science (New York, N.Y.)* **321**, 1807-1812, doi:10.1126/science.1164382 (2008).

25 Yan, H. *et al.* IDH1 and IDH2 mutations in gliomas. *The New England journal of medicine* **360**, 765-773, doi:10.1056/NEJMoa0808710 (2009).

26 Marcucci, G. *et al.* IDH1 and IDH2 gene mutations identify novel molecular subsets within de novo cytogenetically normal acute myeloid leukemia: a Cancer and Leukemia Group B study. *Journal of clinical oncology : official journal of the American Society of Clinical Oncology* **28**, 2348-2355, doi:10.1200/jco.2009.27.3730 (2010).

27 Mardis, E. R. *et al.* Recurring mutations found by sequencing an acute myeloid leukemia genome. *The New England journal of medicine* **361**, 1058-1066, doi:10.1056/NEJMoa0903840 (2009).

28 Amary, M. F. *et al.* IDH1 and IDH2 mutations are frequent events in central chondrosarcoma and central and periosteal chondromas but not in other mesenchymal tumours. *The Journal of pathology* **224**, 334-343, doi:10.1002/path.2913 (2011).

29 Losman, J. A. & Kaelin, W. G., Jr. What a difference a hydroxyl makes: mutant IDH, (R)-2-hydroxyglutarate, and cancer. *Genes & development* **27**, 836-852, doi:10.1101/gad.217406.113 (2013).

30 Zhao, S. *et al.* Glioma-derived mutations in IDH1 dominantly inhibit IDH1 catalytic activity and induce HIF-1alpha. *Science (New York, N.Y.)* **324**, 261-265, doi:10.1126/science.1170944 (2009).

31 Dang, L. *et al.* Cancer-associated IDH1 mutations produce 2-hydroxyglutarate. *Nature* **462**, 739-744, doi:10.1038/nature08617 (2009).

32 Ward, P. S. *et al.* The common feature of leukemia-associated IDH1 and IDH2 mutations is a neomorphic enzyme activity converting alpha-ketoglutarate to 2-hydroxyglutarate. *Cancer cell* **17**, 225-234, doi:10.1016/j.ccr.2010.01.020 (2010).

33 Lu, C. *et al.* IDH mutation impairs histone demethylation and results in a block to cell differentiation. *Nature* **483**, 474-478, doi:10.1038/nature10860 (2012).

34 Sasaki, M. *et al.* IDH1(R132H) mutation increases murine haematopoietic progenitors and alters epigenetics. *Nature* **488**, 656-659, doi:10.1038/nature11323 (2012).

35 Figueroa, M. E. *et al.* Leukemic IDH1 and IDH2 mutations result in a hypermethylation phenotype, disrupt TET2 function, and impair hematopoietic differentiation. *Cancer cell* **18**, 553-567, doi:10.1016/j.ccr.2010.11.015 (2010).

36 Turcan, S. *et al.* IDH1 mutation is sufficient to establish the glioma hypermethylator phenotype. *Nature* **483**, 479-483, doi:10.1038/nature10866 (2012).

37 Losman, J. A. *et al.* (R)-2-hydroxyglutarate is sufficient to promote leukemogenesis and its effects are reversible. *Science (New York, N.Y.)* **339**, 1621-1625, doi:10.1126/science.1231677 (2013).

38 Capper, D. *et al.* Characterization of R132H mutation-specific IDH1 antibody binding in brain tumors. *Brain pathology (Zurich, Switzerland)* **20**, 245-254, doi:10.1111/j.1750-3639.2009.00352.x (2010).

39 Capper, D., Zentgraf, H., Balss, J., Hartmann, C. & von Deimling, A. Monoclonal antibody specific for IDH1 R132H mutation. *Acta neuropathologica* **118**, 599-601, doi:10.1007/s00401-009-0595-z (2009).

40 Watanabe, T., Nobusawa, S., Kleihues, P. & Ohgaki, H. IDH1 mutations are early events in the development of astrocytomas and oligodendrogliomas. *The American journal of pathology* **174**, 1149-1153, doi:10.2353/ajpath.2009.080958 (2009).

41 Weiler, M. & Wick, W. Molecular predictors of outcome in low-grade glioma. *Current opinion in neurology* **25**, 767-773, doi:10.1097/WCO.0b013e32835a0217 (2012).

42 Rohle, D. *et al.* An inhibitor of mutant IDH1 delays growth and promotes differentiation of glioma cells. *Science (New York, N.Y.)* **340**, 626-630, doi:10.1126/science.1236062 (2013).

43 Wang, F. *et al.* Targeted inhibition of mutant IDH2 in leukemia cells induces cellular differentiation. *Science (New York, N.Y.)* **340**, 622-626, doi:10.1126/science.1234769 (2013).

44 Khan-Farooqi, H. R., Prins, R. M. & Liau, L. M. Tumor immunology, immunomics and targeted immunotherapy for central nervous system malignancies. *Neurological research* **27**, 692-702, doi:10.1179/016164105x49490 (2005).

45 Dunn, G. P., Dunn, I. F. & Curry, W. T. Focus on TILs: Prognostic significance of tumor infiltrating lymphocytes in human glioma. *Cancer immunity* **7**, 12 (2007).

46 Ransohoff, R. M., Kivisakk, P. & Kidd, G. Three or more routes for leukocyte migration into the central nervous system. *Nature reviews. Immunology* **3**, 569-581, doi:10.1038/nri1130 (2003).

47 Klug, F. *et al.* Low-dose irradiation programs macrophage differentiation to an iNOS(+)/M1 phenotype that orchestrates effective T cell immunotherapy. *Cancer cell* **24**, 589-602, doi:10.1016/j.ccr.2013.09.014 (2013).

48 Liu, G. *et al.* Cytotoxic T cell targeting of TRP-2 sensitizes human malignant glioma to chemotherapy. *Oncogene* **24**, 5226-5234, doi:10.1038/sj.onc.1208519 (2005).

49 Sampson, J. H. *et al.* Greater chemotherapy-induced lymphopenia enhances tumor-specific immune responses that eliminate EGFRvIII-expressing tumor cells in patients with glioblastoma. *Neuro-oncology* **13**, 324-333, doi:10.1093/neuonc/noq157 (2011).

50 Heimberger, A. B. *et al.* Epidermal growth factor receptor variant III (EGFRvIII) vaccine (CDX-110) in GBM. *Journal of Clinical Oncology* **27**, 2021 (2009).

51 Schuster, J. *et al.* A phase II, multicenter trial of rindopepimut (CDX-110) in newly diagnosed glioblastoma: the ACT III study. *Neuro-oncology*, doi:10.1093/neuonc/nou348 (2015).

52 Wheeler, C. J. & Black, K. L. DCVax-Brain and DC vaccines in the treatment of GBM. *Expert opinion on investigational drugs* **18**, 509-519, doi:10.1517/13543780902841951 (2009).

53 Dutoit, V. *et al.* Exploiting the glioblastoma peptidome to discover novel tumour-associated antigens for immunotherapy. *Brain : a journal of neurology* **135**, 1042-1054, doi:10.1093/brain/aws042 (2012).

54 Xu, L. W., Chow, K. K. & Lim, M. Current vaccine trials in glioblastoma: a review. **2014**, 796856, doi:10.1155/2014/796856 (2014).

55 Schumacher, T. *et al.* A vaccine targeting mutant IDH1 induces antitumour immunity. *Nature*, doi:10.1038/nature13387 (2014).

56 Bunse, L. *et al.* Proximity ligation assay evaluates IDH1R132H presentation in gliomas. *The Journal of clinical investigation* **125**, 593-606, doi:10.1172/jci77780 (2015).

57 Platten, M., Schumacher, T. & Wick, W. Means and methods for treating or diagnosing idh1 r132h mutant-positive cancers. *Google Patents* (2013).

58 Quezada, S. A. *et al.* Tumor-reactive CD4(+) T cells develop cytotoxic activity and eradicate large established melanoma after transfer into lymphopenic hosts. *The Journal of experimental medicine* **207**, 637-650, doi:10.1084/jem.20091918 (2010).

59 Hunder, N. N. *et al.* Treatment of metastatic melanoma with autologous CD4+ T cells against NY-ESO-1. *The New England journal of medicine* **358**, 2698-2703, doi:10.1056/NEJMoa0800251 (2008).

60 Haabeth, O. A. *et al.* How Do CD4(+) T Cells Detect and Eliminate Tumor Cells That Either Lack or Express MHC Class II Molecules? *Frontiers in immunology* **5**, 174, doi:10.3389/fimmu.2014.00174 (2014).

61 Flies, D. B. *et al.* Coinhibitory receptor PD-1H preferentially suppresses CD4(+) T cell-mediated immunity. *Journal of immunology research* **124**, 1966-1975, doi:10.1155/2014/796856
10.1172/jci74589 (2014).

62 Wiestler, B. *et al.* Integrated DNA methylation and copy-number profiling identify three clinically and biologically relevant groups of anaplastic glioma. *Acta neuropathologica*, doi:10.1007/s00401-014-1315-x (2014).

63 Cairncross, J. G. *et al.* Benefit from procarbazine, lomustine, and vincristine in oligodendroglial tumors is associated with mutation of IDH. *Journal of clinical oncology : official journal of the American Society of Clinical Oncology* **32**, 783-790, doi:10.1200/JCO.2013.49.3726 (2014).

64 Polyzoidis, S. & Keyoumars, A. DCVax(R)-L-developed by Northwest Biotherapeutics. *Human vaccines & immunotherapeutics* **10** (2014).

65 Sampson, J. H. *et al.* Immunologic escape after prolonged progression-free survival with epidermal growth factor receptor variant III peptide vaccination in patients with newly diagnosed glioblastoma. *Journal of clinical oncology : official journal of the American Society of Clinical Oncology* **28**, 4722-4729, doi:10.1200/jco.2010.28.6963 (2010).

66 Reyes-Botero, G. *et al.* Contrast enhancement in 1p/19q-codeleted anaplastic oligodendrogliomas is associated with 9p loss, genomic instability, and angiogenic gene expression. *Neuro-oncology* **16**, 662-670, doi:10.1093/neuonc/not235 (2014).

67 Ajay, D., Sanchez-Perez, L., Choi, B. D., De Leon, G. & Sampson, J. H. Immunotherapy with tumor vaccines for the treatment of malignant gliomas. *Current drug discovery technologies* **9**, 237-255 (2012).

68 Fadul, C. E. *et al.* Immune modulation effects of concomitant temozolomide and radiation therapy on peripheral blood mononuclear cells in patients with glioblastoma multiforme. *Neuro-oncology* **13**, 393-400, doi:10.1093/neuonc/noq204 (2011).

69 Sampson, J. H. *et al.* A pilot study of IL-2Ralpha blockade during lymphopenia depletes regulatory T-cells and correlates with enhanced immunity in patients with glioblastoma. *PloS one* **7**, e31046, doi:10.1371/journal.pone.0031046 (2012).

70 Wen, P. Y. *et al.* A randomized, double-blind, placebo-controlled phase 2 trial of dendritic cell (DC) vaccination with ICT-107 in newly diagnosed glioblastoma (GBM) patients. *Journal of clinical oncology : official journal of the American Society of Clinical Oncology* **32:5s (suppl; abstr 2005)** (2014).

71 Lennerz, V. *et al.* The response of autologous T cells to a human melanoma is dominated by mutated neoantigens. *Proceedings of the National Academy of Sciences of the United States of America* **102**, 16013-16018, doi:10.1073/pnas.0500090102 (2005).

72 Robbins, P. F. *et al.* Mining exomic sequencing data to identify mutated antigens recognized by adoptively transferred tumor-reactive T cells. *Nature medicine* **19**, 747-752, doi:10.1038/nm.3161 (2013).

73 Wolfel, T. *et al.* A p16INK4a-insensitive CDK4 mutant targeted by cytolytic T lymphocytes in a human melanoma. *Science (New York, N.Y.)* **269**, 1281-1284 (1995).

74 van Rooij, N. *et al.* Tumor exome analysis reveals neoantigen-specific T-cell reactivity in an ipilimumab-responsive melanoma. *Journal of clinical oncology : official journal of the American Society of Clinical Oncology* **31**, e439-442, doi:10.1200/jco.2012.47.7521 (2013).

75 Linnemann, C. *et al.* High-throughput epitope discovery reveals frequent recognition of neo-antigens by CD4+ T cells in human melanoma. *Nature medicine* **21**, 81-85, doi:10.1038/nm.3773 (2015).

76 Carbone, D. P. *et al.* Immunization with mutant p53- and K-ras-derived peptides in cancer patients: immune response and clinical outcome. *Journal of clinical oncology : official journal of the American Society of Clinical Oncology* **23**, 5099-5107, doi:10.1200/jco.2005.03.158 (2005).

77 Khleif, S. N. *et al.* A phase I vaccine trial with peptides reflecting ras oncogene mutations of solid tumors. *Journal of immunotherapy (Hagerstown, Md. : 1997)* **22**, 155-165 (1999).

78 Rahma, O. E. *et al.* A pilot clinical trial testing mutant von Hippel-Lindau peptide as a novel immune therapy in metastatic renal cell carcinoma. *Journal of translational medicine* **8**, 8, doi:10.1186/1479-5876-8-8 (2010).

79 Heimberger, A. B. & Sampson, J. H. The PEPvIII-KLH (CDX-110) vaccine in glioblastoma multiforme patients. *Expert opinion on biological therapy* **9**, 1087-1098, doi:10.1517/14712590903124346 (2009).

80 Weller, M., Cloughesy, T., Perry, J. R. & Wick, W. Standards of care for treatment of recurrent glioblastoma--are we there yet? *Neuro-oncology* **15**, 4-27, doi:10.1093/neuonc/nos273 (2013).

81 Schön, M. P. & Schön, M. Imiquimod: mode of action. *The British journal of dermatology* **157 Suppl 2**, 8-13, doi:10.1111/j.1365-2133.2007.08265.x (2007).

82 European Medicines Agency. Explanatory note on immunomodulators for the guideline on adjuvants in vaccines for human use. *EMEA/CHMP/VWP/244894/2006* (2006).

83 Parmiani, G., Pilla, L., Castelli, C. & Rivoltini, L. Vaccination of patients with solid tumours. *Annals of oncology : official journal of the European Society for Medical Oncology / ESMO* **14**, 817-824 (2003).

84 Greiner, J., Dohner, H. & Schmitt, M. Cancer vaccines for patients with acute myeloid leukemia--definition of leukemia-associated antigens and current clinical protocols targeting these antigens. *Haematologica* **91**, 1653-1661 (2006).

85 Greiner, J. *et al.* High-dose RHAMM-R3 peptide vaccination for patients with acute myeloid leukemia, myelodysplastic syndrome and multiple myeloma. *Haematologica* **95**, 1191-1197, doi:10.3324/haematol.2009.014704 (2010).

86 Schmitt, M. *et al.* RHAMM-R3 peptide vaccination in patients with acute myeloid leukemia, myelodysplastic syndrome, and multiple myeloma elicits immunologic and clinical responses. *Blood* **111**, 1357-1365, doi:10.1182/blood-2007-07-099366 (2008).

87 Aucouturier, J., Dupuis, L., Deville, S., Ascarateil, S. & Ganne, V. Montanide ISA 720 and 51: a new generation of water in oil emulsions as adjuvants for human vaccines. *Expert review of vaccines* **1**, 111-118, doi:10.1586/14760584.1.1.111 (2002).

88 Sul, J. *et al.* A phase I trial of peptide-based glioma vaccine IMA950 in patients with glioblastoma: study design and preliminary results. *Neuro-oncology* **15 (suppl 3)**, iii68-iii74, doi:10.1093/neuonc/not178 (2013).

89 Casalegno-Garduno, R., Schmitt, A. & Schmitt, M. Clinical peptide vaccination trials for leukemia patients. *Expert review of vaccines* **10**, 785-799, doi:10.1586/erv.11.56 (2011).

90 Clancy-Thompson, E. *et al.* Peptide vaccination in Montanide adjuvant induces and GM-CSF increases CXCR3 and cutaneous lymphocyte antigen expression by tumor antigen-specific CD8 T cells. *Cancer immunology research* **1**, 332-339, doi:10.1158/2326-6066.cir-13-0084 (2013).

91 Tsuji, T. *et al.* Effect of Montanide and poly-ICLC adjuvant on human self/tumor antigen-specific CD4+ T cells in phase I overlapping long peptide vaccine trial. *Cancer immunology research* **1**, 340-350, doi:10.1158/2326-6066.cir-13-0089 (2013).

92 Wada, H. *et al.* Vaccination with NY-ESO-1 overlapping peptides mixed with Picibanil OK-432 and montanide ISA-51 in patients with cancers expressing the NY-ESO-1 antigen. *Journal of immunotherapy (Hagerstown, Md. : 1997)* **37**, 84-92, doi:10.1097/cji.0000000000000017 (2014).

93 Chuang, C. M., Monie, A., Hung, C. F. & Wu, T. C. Treatment with imiquimod enhances antitumor immunity induced by therapeutic HPV DNA vaccination. *Journal of biomedical science* **17**, 32, doi:10.1186/1423-0127-17-32 (2010).

94 Demaria, S., Vanpouille-Box, C., Formenti, S. C. & Adams, S. The TLR7 agonist imiquimod as an adjuvant for radiotherapy-elicited in situ vaccination against breast cancer. *Oncoimmunology* **2**, e25997, doi:10.4161/onci.25997 (2013).

95 Fenoglio, D. *et al.* A multi-peptide, dual-adjuvant telomerase vaccine (GX301) is highly immunogenic in patients with prostate and renal cancer. *Cancer immunology, immunotherapy : CII* **62**, 1041-1052, doi:10.1007/s00262-013-1415-9 (2013).

96 Feyerabend, S. *et al.* Novel multi-peptide vaccination in Hla-A2+ hormone sensitive patients with biochemical relapse of prostate cancer. *The Prostate* **69**, 917-927, doi:10.1002/pros.20941 (2009).

97 Goldinger, S. M. *et al.* Nano-particle vaccination combined with TLR-7 and -9 ligands triggers memory and effector CD8(+) T-cell responses in melanoma patients. *European journal of immunology* **42**, 3049-3061, doi:10.1002/eji.201142361 (2012).

98 Iversen, T. Z. *et al.* Long-lasting disease stabilization in the absence of toxicity in metastatic lung cancer patients vaccinated with an epitope derived from indoleamine 2,3 dioxygenase. *Clinical cancer research : an official journal of the American Association for Cancer Research* **20**, 221-232, doi:10.1158/1078-0432.ccr-13-1560 (2014).

99 Shackleton, M. *et al.* The impact of imiquimod, a Toll-like receptor-7 ligand (TLR7L), on the immunogenicity of melanoma peptide vaccination with adjuvant Flt3 ligand. *Cancer immunity* **4**, 9 (2004).

100 Miyagi, Y. *et al.* Induction of cellular immune responses to tumor cells and peptides in colorectal cancer patients by vaccination with SART3 peptides. *Clinical cancer research : an official journal of the American Association for Cancer Research* **7**, 3950-3962 (2001).

101 Giannopoulos, K. *et al.* Peptide vaccination elicits leukemia-associated antigen-specific cytotoxic CD8+ T-cell responses in patients with chronic lymphocytic leukemia. *Leukemia* **24**, 798-805, doi:10.1038/leu.2010.29 (2010).

102 Schmitt, M. *et al.* Cytomegalovirus peptide vaccination results in clearance of the virus load. *Bone Marrow Transplant* **49(Suppl. 1)**, O021 (2014).

103 Okada, H. *et al.* Induction of CD8+ T-cell responses against novel glioma-associated antigen peptides and clinical activity by vaccinations with {alpha}-type 1 polarized dendritic cells and polyinosinic-polycytidylic acid stabilized by lysine and carboxymethylcellulose in patients with recurrent malignant glioma. *Journal of clinical oncology : official journal of the American Society of Clinical Oncology* **29**, 330-336, doi:10.1200/jco.2010.30.7744 (2011).

104 Karnofsky, D. A. & Burchenal, J. H. *The Clinical Evaluation of Chemotherapeutic Agents in Cancer. In: Evaluation of Chemotherapeutic Agents*. (Columbia Univ Press, 1949).

105 Folstein, M. F., Folstein, S. E. & McHugh, P. R. "Mini-mental state". A practical method for grading the cognitive state of patients for the clinician. *Journal of psychiatric research* **12**, 189-198 (1975).

106 Wen, P. Y. *et al.* Updated response assessment criteria for high-grade gliomas: response assessment in neuro-oncology working group. *Journal of clinical oncology : official journal of the American Society of Clinical Oncology* **28**, 1963-1972, doi:10.1200/jco.2009.26.3541 (2010).

107 Choi, C. *et al.* 2-hydroxyglutarate detection by magnetic resonance spectroscopy in IDH-mutated patients with gliomas. *Nature medicine* **18**, 624-629, doi:10.1038/nm.2682 (2012).

108 Heimberger, A. B. *et al.* Incidence and prognostic impact of FoxP3+ regulatory T cells in human gliomas. *Clinical cancer research : an official journal of the American Association for Cancer Research* **14**, 5166-5172, doi:10.1158/1078-0432.ccr-08-0320 (2008).

109 Wick, W. & Weller, M. How lymphotoxic is dose-intensified temozolomide? The glioblastoma experience. *Journal of clinical oncology : official journal of the American Society of Clinical Oncology* **23**, 4235-4236; author reply 4236, doi:10.1200/jco.2004.00.8417 (2005).

110 Aamot, H., Gigic, B., Abel, U. & Karapanagiotou-Schenkel, I. Continuous monitoring of toxicity in clinical trials - simulating the risk of stopping prematurely. *International journal of clinical pharmacology and therapeutics* **48**, 476-477 (2010).

111 DIMDI. ICD-10-GM Version 2014: Mini Mental State Examination (MMSE). *http://www.dimdi.de/static/de/klassi/icd-10-gm/kodesuche/onlinefassungen/htmlgm2014/zusatz-10-mmse.htm#top (accessed 15.08.2014)*.

18 Appendices

Appendix I: Measurement of Safety Parameters

Performance Status

Performance status will be evaluated regarding the Karnofsky Performane Score (KPS) [104].

Karnofsky Performance Status (KPS)

Rating [%]	Criteria
100	Normal no complaints; no evidence of disease.
90	Able to carry on normal activity; minor signs or symptoms of disease.
80	Normal activity with effort; some signs or symptoms of disease.
70	Cares for self; unable to carry on normal activity or to do active work.
60	Requires occasional assistance, but is able to care for most of his personal needs.
50	Requires considerable assistance and frequent medical care.
40	Disabled; requires special care and assistance.
30	Severely disabled; hospital admission is indicated although death not imminent.
20	Very sick; hospital admission necessary; active supportive treatment necessary.
10	Moribund; fatal processes progressing rapidly.
0	Dead

Mini Mental Status Examination

MMSE will be performed in German regarding the following scheme [105,111]:

Funktionen		Punkte
**I. Orientierung**		
**1.**	Datum	**1 / 0**
**2.**	Jahreszeit	**1 / 0**
**3.**	Jahr	**1 / 0**
**4.**	Wochentag	**1 / 0**
**5.**	Monat<br>*Zuerst nach dem Datum fragen, dann gezielt nach den noch fehlenden Punkten (z. B. "Können Sie mir auch sagen, welche Jahreszeit jetzt ist?")*	**1 / 0**
**6.**	Bundesland	**1 / 0**
**7.**	Landeskreis/Stadt	**1 / 0**
**8.**	Stadt/Stadtteil	**1 / 0**
**9.**	Krankenhaus	**1 / 0**
**10.**	Station/Stockwerk<br>*Zuerst nach dem Namen der Klinik fragen, dann nach Station/Stockwerk, Stadt/Stadtteil usw. fragen. In Großstädten sollte nicht nach Stadt und Landkreis, sondern nach Stadt und Stadtteil gefragt werden. Gefragt wird in*	**1 / 0**

	jedem Fall nach dem aktuellen Aufenthaltsort und nicht nach dem Wohnort.	
**II. Merkfähigkeit**		
Der Untersuchte muss zuerst gefragt werden, ob er mit einem kleinen Gedächtnistest einverstanden ist. Er wird darauf hingewiesen, dass er sich 3 Begriffe merken soll.<br>*Die Begriffe langsam und deutlich - im Abstand von jeweils ca. 1 Sekunde - nennen. Direkt danach die 3 Begriffe wiederholen lassen, der erste Versuch bestimmt die Punktzahl. Ggf. wiederholen, bis der Untersuchte alle 3 Begriffe gelernt hat. Die Anzahl der notwendigen Versuche zählen und notieren (max. 6 Versuche zulässig). Wenn nicht alle 3 Begriffe gelernt wurden, kann der Gedächtnistest nicht durchgeführt werden.*		
**11.**	Apfel	**1 / 0**
**12.**	Pfennig	**1 / 0**
**13.**	Tisch	**1 / 0**
**III. Aufmerksamkeit und Rechenfertigkeit**		
Beginnend bei 100 muss fünfmal jeweils 7 subtrahiert werden. Jeden einzelnen Rechenschritt unabhängig vom vorangehenden beurteilen, damit ein Fehler nicht mehrfach gewertet wird.<br>*Alternativ (z. B. wenn der Untersuchte nicht rechnen kann oder will) kann in Ausnahmefällen das Wort "STUHL" rückwärts buchstabiert werden. Das Wort sollte zunächst vorwärts buchstabiert und wenn nötig korrigiert werden. Die Punktzahl ergibt sich dann aus der Anzahl der Buchstaben, die in der richtigen Reihenfolge genannt werden (z. B. "LHTUS" = 3 Punkte).*		
**14.**	< 93 >	**1 / 0**
**15.**	< 86 >	**1 / 0**
**16.**	< 79 >	**1 / 0**
**17.**	< 72 >	**1 / 0**
**18.**	< 65 >	**1 / 0**
**19.**	alternativ: "STUHL" rückwärts buchstabieren LHUTS	**5/4/3/2/1/0**
**IV. Erinnerungsfähigkeit**		
Der Untersuchte muss die 3 Begriffe nennen, die er sich merken sollte.		
**20.**	Apfel	**1 / 0**
**21.**	Pfennig	**1 / 0**
**22.**	Tisch	**1 / 0**
**V. Sprache**		
Eine Uhr und ein Stift werden gezeigt, der Untersuchte muss diese richtig benennen.		
**23.**	Armbanduhr benennen	**1 / 0**
**24.**	Bleistift benennen	**1 / 0**
**25.**	Nachsprechen des Satzes "kein wenn und oder aber" (max. 3 Wdh.)<br>*Der Satz muss unmittelbar nachgesprochen werden, nur 1 Versuch ist erlaubt. Es ist nicht zulässig, die Redewendung "Kein wenn und aber" zu benutzen.*	**1 / 0**
**26.**	Kommandos befolgen:	

	Der Untersuchte erhält ein Blatt Papier, der dreistufige Befehl wird nur einmal erteilt. 1 Punkt für jeden Teil, der korrekt befolgt wird.	
	- Nehmen Sie bitte das Papier in die Hand	**1 / 0**
	- Falten Sie es in der Mitte	**1 / 0**
	- Lassen Sie es auf den Boden fallen	**1 / 0**
**27.**	Schriftliche Anweisungen befolgen "AUGEN ZU":<br>*Die Buchstaben ("AUGEN ZU") müssen so groß sein, dass sie auch bei eingeschränktem Visus noch lesbar sind. 1 Punkt wird nur dann gegeben, wenn die Augen wirklich geschlossen sind.*	**1 / 0**
**28.**	Schreiben Sie bitte irgendeinen Satz:<br>*Es darf kein Satz diktiert werden, die Ausführung muss spontan erfolgen. Der Satz muss Subjekt und Prädikat enthalten und sinnvoll sein. Korrekte Grammatik und Interpunktion ist nicht gefordert. Das Schreiben von Namen und Anschrift ist nicht ausreichend.*	**1 / 0**
**29.**	Fünfecke nachzeichnen:<br>*Auf einem Blatt Papier sind 2 sich überschneidende Fünfecke dargestellt, der Untersuchte soll diese so exakt wie möglich abzeichnen. Alle 10 Ecken müssen wiedergegeben sein und 2 davon sich überschneiden, nur dann wird 1 Punkt gegeben.*<br>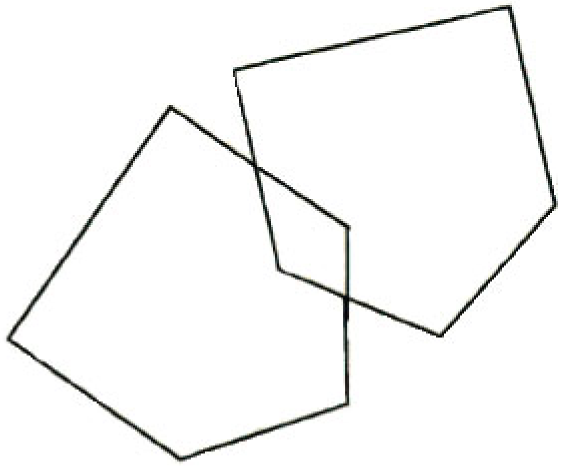	**1 / 0**
**Summe maximal**		**30**

© Copyright WHO, DIMDI 1994 – 2014 [111]

Appendix II: Disease Assessment by RANO Criteria

The relevant publication regarding disease assessment by RANO criteria is attached on the following pages [106]:

VOLUME 28 · NUMBER 11 · APRIL 10 2010

JOURNAL OF CLINICAL ONCOLOGY

SPECIAL ARTICLE

Updated Response Assessment Criteria for High-Grade Gliomas: Response Assessment in Neuro-Oncology Working Group

Patrick Y. Wen, David R. Macdonald, David A. Reardon, Timothy F. Cloughesy, A. Gregory Sorensen, Evanthia Galanis, John DeGroot, Wolfgang Wick, Mark R. Gilbert, Andrew B. Lassman, Christina Tsien, Tom Mikkelsen, Eric T. Wong, Marc C. Chamberlain, Roger Stupp, Kathleen R. Lamborn, Michael A. Vogelbaum, Martin J. van den Bent, and Susan M. Chang

From the Center for Neuro-Oncology, Dana-Farber/Brigham and Women's Cancer Center; Division of Neurology, Brigham and Women's Hospital; Department of Radiology, Massachusetts General Hospital; Brain Tumor Center, Department of Neurology, Beth Israel Deaconess Medical Center, Boston, MA; Preston Robert Tisch Brain Tumor Center, Duke University Medical Center, Durham, NC; Neuro-Oncology Program, David Geffen School of Medicine at University of California, Los Angeles, Los Angeles; Division of Neuro-Oncology, Department of Neurological Surgery, University of California, San Francisco, San Francisco, CA; Department of Medical Oncology, Mayo Clinic, Rochester, MN; Department of Neuro-Oncology, The University of Texas M.D. Anderson Cancer Center, Houston, TX; Department of Neurology and Brain Tumor Center, Memorial Sloan-Kettering Cancer Center, New York, NY; Department of Radiation Oncology, University of Michigan Medical Center, Ann Arbor; Department of Neuro-Oncology, Henry Ford Hospital, Detroit, MI; Fred Hutchinson Cancer Center, Seattle, WA; Brain Tumor and Neuro-Oncology Center, Department of Neurosurgery, Cleveland Clinic, Cleveland OH; Department of Medical Oncology, London Regional Cancer Program, University of Western Ontario, London, Ontario, Canada; Department of Neuro-Oncology, University of Heidelberg, Heidelberg, Germany; Centre Hospitalier Universitaire Vaudois; University of Lausanne, Lausanne, Switzerland; and Neuro-Oncology Unit, Daniel den Hoed Cancer Center/Erasmus University Hospital, Rotterdam, the Netherlands.

Submitted September 26, 2009; accepted December 14, 2009; published online ahead of print at www.jco.org on March 15, 2010.

P.Y.W., D.R.M., M.A.V., M.J.v.d.B. and S.M.C. contributed equally to this work.

Authors' disclosures of potential conflicts of interest and author contributions are found at the end of this article.

Corresponding author: Patrick Y. Wen, MD, Center for Neuro-Oncology, Dana Farber/Brigham and Women's Cancer Center, SW430D, 44 Binney St, Boston, MA 02115; e-mail: pwen@partners.org.

© 2010 by American Society of Clinical Oncology

0732-183X/10/2811-1963/$20.00

DOI: 10.1200/JCO.2009.26.3541

ABSTRACT

Currently, the most widely used criteria for assessing response to therapy in high-grade gliomas are based on two-dimensional tumor measurements on computed tomography (CT) or magnetic resonance imaging (MRI), in conjunction with clinical assessment and corticosteroid dose (the Macdonald Criteria). It is increasingly apparent that there are significant limitations to these criteria, which only address the contrast-enhancing component of the tumor. For example, chemoradiotherapy for newly diagnosed glioblastomas results in transient increase in tumor enhancement (pseudoprogression) in 20% to 30% of patients, which is difficult to differentiate from true tumor progression. Antiangiogenic agents produce high radiographic response rates, as defined by a rapid decrease in contrast enhancement on CT/MRI that occurs within days of initiation of treatment and that is partly a result of reduced vascular permeability to contrast agents rather than a true antitumor effect. In addition, a subset of patients treated with antiangiogenic agents develop tumor recurrence characterized by an increase in the nonenhancing component depicted on T2-weighted/fluid-attenuated inversion recovery sequences. The recognition that contrast enhancement is nonspecific and may not always be a true surrogate of tumor response and the need to account for the nonenhancing component of the tumor mandate that new criteria be developed and validated to permit accurate assessment of the efficacy of novel therapies. The Response Assessment in Neuro-Oncology Working Group is an international effort to develop new standardized response criteria for clinical trials in brain tumors. In this proposal, we present the recommendations for updated response criteria for high-grade gliomas.

J Clin Oncol 28:1963-1972. © 2010 by American Society of Clinical Oncology

INTRODUCTION

Gliomas are the most common form of malignant primary brain tumors in adults, with an annual incidence of approximately four to five per 100,000 people.[1,2] The evaluation of treatment in high-grade gliomas currently relies either on the duration of patient survival or, more commonly in patients with recurrent disease, the radiographic response rate or progression-free survival (PFS).[3,4] In 1990, Macdonald et al[5] published criteria for response assessment in high-grade gliomas (Table 1). These criteria provided an objective radiologic assessment of tumor response and were based primarily on contrast-enhanced computed tomography (CT) and the two-dimensional WHO oncology response criteria using enhancing tumor area (the product of the maximal cross-sectional enhancing diameters) as the primary tumor measure.[6,7] These criteria also considered the use of corticosteroids and changes in the neurologic status of the patient. The Macdonald Criteria enabled response rates to be compared between clinical trials and have been widely used in high-grade glioma studies since their introduction.

Although the Macdonald Criteria were developed primarily for CT scans, they have been extrapolated to magnetic resonance imaging (MRI), which is now the standard neuroimaging modality used to assess treatment response in high-grade gliomas. Like CT scans, areas of the tumor with abnormal vascular architecture and disrupted integrity of the blood-brain barrier are depicted as the contrast-enhancing component on MRI.[8]

In systemic cancers, one-dimensional tumor measurements have become the standard criteria to determine response. The Response Evaluation Criteria in Solid Tumors (RECIST) first introduced the use of one-dimensional measurements in 2000[9] and were recently revised (RECIST version 1.1).[10] Several studies have compared the RECIST criteria with

Information downloaded from jco.ascopubs.org and provided by at DFG on April 1, 2014 from 193.174.53.85
Copyright © 2010 American Society of Clinical Oncology. All rights reserved.

Table 1. Current Response Criteria for Malignant Gliomas (Macdonald Criteria)[5]

Response	Criteria
Complete response	Requires all of the following: complete disappearance of all enhancing measurable and nonmeasurable disease sustained for at least 4 weeks; no new lesions; no corticosteroids; and stable or improved clinically
Partial response	Requires all of the following: ≥ 50% decrease compared with baseline in the sum of products of perpendicular diameters of all measurable enhancing lesions sustained for at least 4 weeks; no new lesions; stable or reduced corticosteroid dose; and stable or improved clinically
Stable disease	Requires all of the following: does not qualify for complete response, partial response, or progression; and stable clinically
Progression	Defined by any of the following: ≥ 25% increase in sum of the products of perpendicular diameters of enhancing lesions; any new lesion; or clinical deterioration

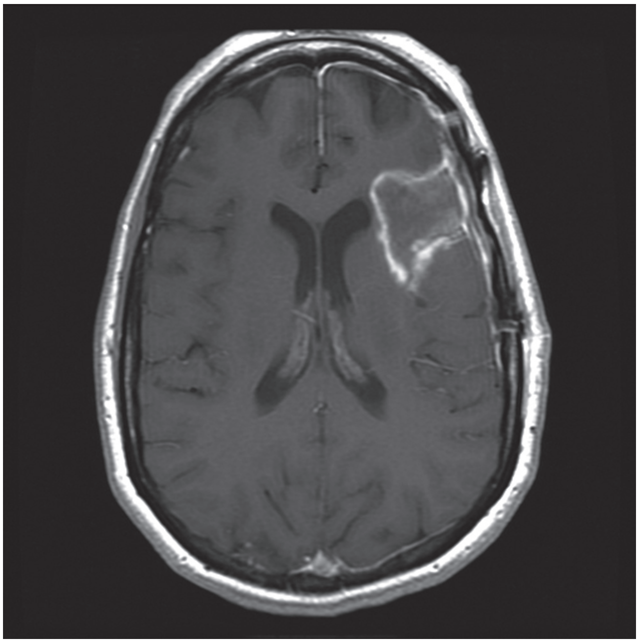

Fig 1. A 38-year-old patient with left frontal glioblastoma showing irregular enhancement in wall of the cavity that is difficult to measure. Although the entire cavity is often measured, it would be preferable if only the enhancing nodule in the posterior wall of the cavity were measured. If it is smaller than 10 mm in bidirectional diameters, the lesion would be considered nonmeasurable.

two-dimensional measurements, three-dimensional measurements, and volumetric measurements in high-grade gliomas.[11-13] These studies suggest that there is good concordance among the different methods in determining response in adult patients with both newly diagnosed and recurrent high-grade gliomas,[12,13] as well as in pediatric brain tumors.[11] However, an exception is seen with three-dimensional measurements, which seem to be inferior to one- and two-dimensional and volumetric measurements.[12,14] Nonetheless, studies prospectively validating the RECIST criteria in gliomas have not been performed. Currently, the Macdonald Criteria using two-dimensional measurement remain the most widely used method for evaluating tumor response in clinical trials of high-grade gliomas, partly because they enable the results of ongoing studies to be easily compared with historical data.

LIMITATIONS OF THE MACDONALD CRITERIA

From their inception, it was apparent that the Macdonald Criteria had a number of important limitations. These limitations, which have recently been reviewed in detail,[15-17] include the difficulty of measuring irregularly shaped tumors, interobserver variability, the lack of assessment of the nonenhancing component of the tumor, lack of guidance for the assessment of multifocal tumors, and the difficulty in measuring enhancing lesions in the wall of cystic or surgical cavities because the cyst/cavity itself may be included in the tumor measurement (Fig 1). In the Macdonald Criteria, a significant increase (at least 25%) in the contrast-enhancing lesion is used as a reliable surrogate marker for tumor progression, and its presence mandates a change in therapy. However, contrast enhancement is nonspecific and primarily reflects the passage of contrast material across a disrupted blood-tumor barrier. Enhancement can be influenced by changes in corticosteroid doses, antiangiogenic agents (discussed later), and changes in radiologic techniques.[18,19] Increased enhancement can also be induced by a variety of nontumoral processes such as treatment-related inflammation, seizure activity, postsurgical changes, ischemia, subacute radiation effects, and radiation necrosis.[20-23] As a result, there are significant limitations in equating changes in enhancing area with changes in tumor size or tumor growth. The limitations of the Macdonald Criteria have become even more apparent with the increased incidence of pseudoprogression in patients receiving radiotherapy with temozolomide and the recent introduction of antiangiogenic therapies that affect the permeability of tumor vasculature. This has led to the current effort to revise the response criteria for high-grade gliomas.[17] The major issues are discussed in the following sections.

Pseudoprogression and Radiation Effects

Standard therapy for glioblastoma involves maximal safe tumor resection followed by radiotherapy with concurrent and adjuvant temozolomide.[24,25] Twenty to 30% of patients undergoing their first postradiation MRI show increased contrast enhancement that eventually subsides without any change in therapy (Fig 2). This phenomenon, termed pseudoprogression, likely results from transiently increased permeability of the tumor vasculature from irradiation, which may be enhanced by temozolomide, and complicates the determination of tumor progression immediately after completion of radiotherapy.[26-30] Pseudoprogression may be accompanied by progressive clinical signs and symptoms and seems to be more frequent in patients with a methylated *MGMT* gene promoter.[30] This treatment-related effect has implications for patient management and may result in premature discontinuation of effective adjuvant therapy. This limits the validity of a PFS end point unless tissue-based confirmation of tumor progression is obtained. It also has significant implications for selecting appropriate patients for participation in clinical trials for recurrent gliomas. Failure to exclude patients with pseudoprogression from these studies will result in a falsely high response rate and PFS

 © 2010 by American Society of Clinical Oncology

Information downloaded from jco.ascopubs.org and provided by at DFG on April 1, 2014 from 193.174.53.85
Copyright © 2010 American Society of Clinical Oncology. All rights reserved.

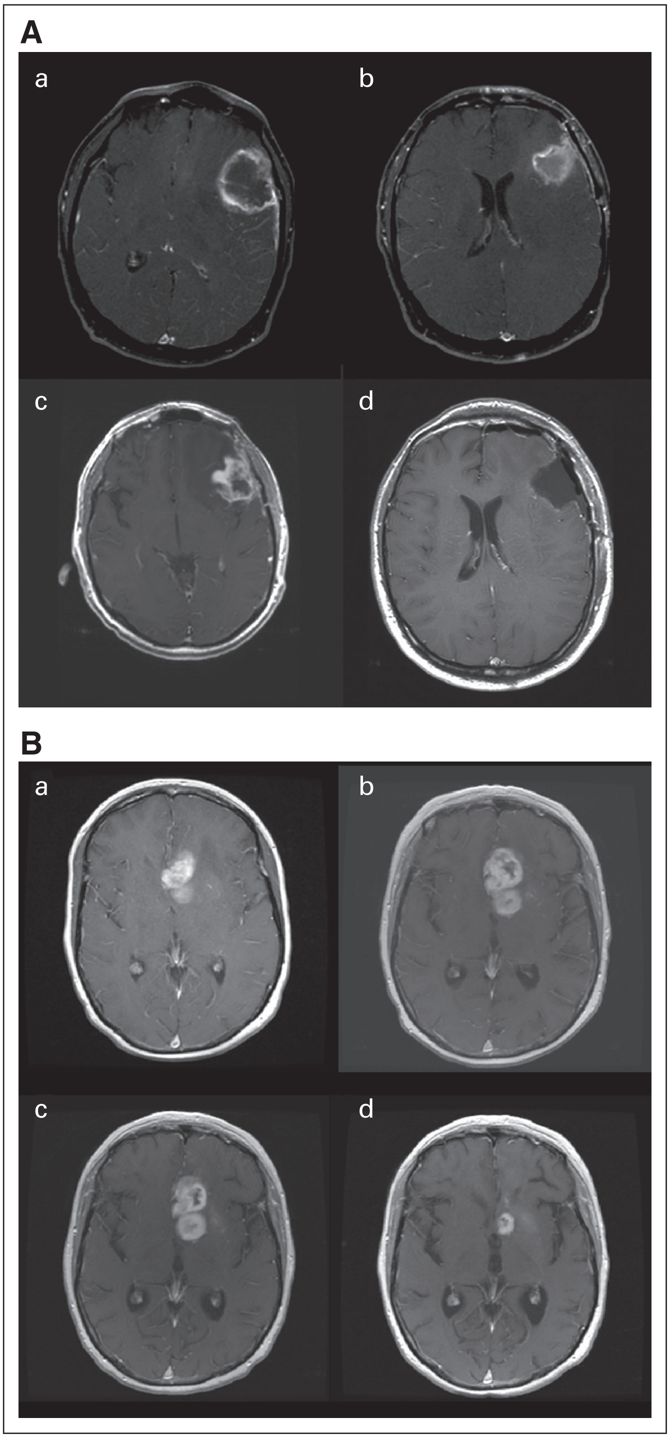

Fig 2. (A) Pseudoprogression after chemoradiotherapy: axial T1-contrast enhanced magnetic resonance imaging (MRI) a) before surgery; b) after surgery; c) after radiotherapy and concomitant temozolomide showing increased enhancement; d) re-operation showing only necrotic tissue and no tumor. (B) Pseudoprogression after chemoradiotherapy: axial T1-contrast enhanced MRI showing deep left frontal glioblastoma a) 2 days after stereotactic biopsy; b) 4 weeks after radiotherapy and concomitant temozolomide showing increased enhancement, raising the possibility of progression; c) after 4 additional weeks of treatment with adjuvant temozolomide showing stable disease; d) after 8 cycles of adjuvant temozolomide showing significant reduction in tumor size.

and the possibility that an agent will be incorrectly considered to be active. To address this issue, the proposed new response criteria suggest that within the first 12 weeks of completion of radiotherapy, when pseudoprogression is most prevalent, progression can only be determined if the majority of the new enhancement is outside of the radiation field (for example, beyond the high-dose region or 80% isodose line) or if there is pathologic confirmation of progressive disease (Table 2). It is recognized that the proposed histologic criteria have important limitations, but they provide guidance on the type of findings that are suggestive of progressive disease. For patients in whom pseudoprogression cannot be differentiated from true tumor progression, enrollment onto trials for recurrent gliomas should not be permitted. Patients who remain clinically stable and/or are suspected to have pseudoprogression based on metabolic or vascular imaging should continue with their current therapy.

Enhancement As a Result of Surgery and Other Therapies

Increased enhancement often develops in the wall of the surgical cavity 48 to 72 hours after surgery.[20,31-33] To avoid interpretation of

Table 2. Criteria for Determining First Progression Depending on Time From Initial Chemoradiotherapy

First Progression	Definition
Progressive disease < 12 weeks after completion of chemoradiotherapy	Progression can only be defined using diagnostic imaging if there is new enhancement outside of the radiation field (beyond the high-dose region or 80% isodose line) or if there is unequivocal evidence of viable tumor on histopathologic sampling (eg, solid tumor areas [ie, > 70% tumor cell nuclei in areas], high or progressive increase in MIB-1 proliferation index compared with prior biopsy, or evidence for histologic progression or increased anaplasia in tumor). Note: Given the difficulty of differentiating true progression from pseudoprogression, clinical decline alone, in the absence of radiographic or histologic confirmation of progression, will not be sufficient for definition of progressive disease in the first 12 weeks after completion of concurrent chemoradiotherapy.
Progressive disease ≥ 12 weeks after chemoradiotherapy completion	1. New contrast-enhancing lesion outside of radiation field on decreasing, stable, or increasing doses of corticosteroids.<br>2. Increase by ≥ 25% in the sum of the products of perpendicular diameters between the first postradiotherapy scan, or a subsequent scan with smaller tumor size, and the scan at 12 weeks or later on stable or increasing doses of corticosteroids.<br>3. Clinical deterioration not attributable to concurrent medication or comorbid conditions is sufficient to declare progression on current treatment but not for entry onto a clinical trial for recurrence.<br>4. For patients receiving antiangiogenic therapy, significant increase in T2/FLAIR nonenhancing lesion may also be considered progressive disease. The increased T2/FLAIR must have occurred with the patient on stable or increasing doses of corticosteroids compared with baseline scan or best response after initiation of therapy and not be a result of comorbid events (eg, effects of radiation therapy, demyelination, ischemic injury, infection, seizures, postoperative changes, or other treatment effects).

Abbreviation: FLAIR, fluid-attenuated inversion recovery.

© 2010 by American Society of Clinical Oncology

Information downloaded from jco.ascopubs.org and provided by at DFG on April 1, 2014 from 193.174.53.85
Copyright © 2010 American Society of Clinical Oncology. All rights reserved.

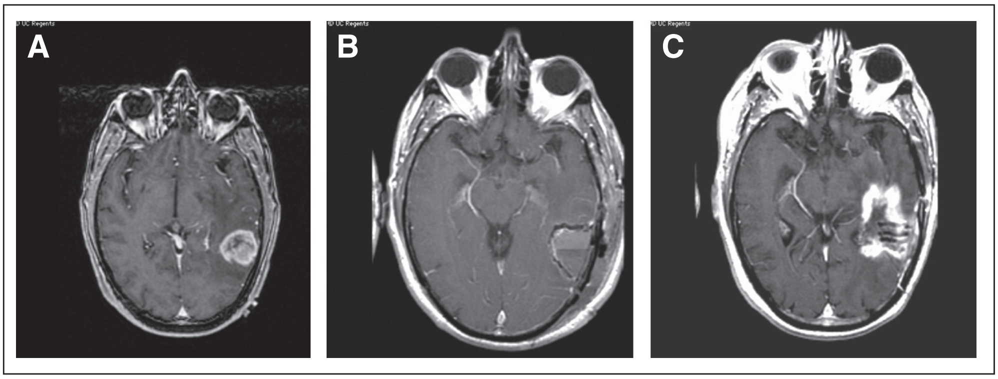

Fig 3. Pseudoprogression after brachytherapy. (A) Axial T1 contrast-enhanced magnetic resonance imaging (MRI) showing enhancing tumor before surgery. (B) Immediate postoperative magnetic resonance imaging (MRI) showing acute surgical changes and placement of iodine-125 brachytherapy seeds. (C) MRI performed 18 months later showing increased enhancement. Reoperation showed no tumor.

postoperative changes as residual enhancing disease, a baseline MRI scan should ideally be obtained within 24 to 48 hours after surgery and no later than 72 hours after surgery. The inclusion of diffusion-weighted imaging in the immediate postoperative MRI scan can be helpful in determining whether new enhancement developing in the subsequent weeks or months is caused by sequelae of ischemia or by tumor recurrence.[16,22] In addition, a transient increase in enhancement that can be difficult to distinguish from recurrent disease can also occur after locally administered therapies. These include chemotherapy wafers, immunotoxins delivered by convection-enhanced delivery, regionally administered gene and viral therapies, immunotherapies, and focal irradiation with brachytherapy and stereotactic radiosurgery (Fig 3).[17,34-38] Imaging modalities such as perfusion imaging, magnetic resonance spectroscopy, and positron emission tomography scans may sometimes be helpful in differentiating treatment effects from recurrent tumor.[39-42] However, no imaging modality currently has sufficient specificity to conclusively differentiate recurrent tumor from treatment effects, and surgical sampling may occasionally be needed to obtain a definitive diagnosis.

Pseudoresponses After Treatment With Antiangiogenic Therapies

Antiangiogenic agents, especially those targeting vascular endothelial growth factor (VEGF), such as bevacizumab, and the VEGF receptor, such as cediranib, can produce marked decrease in contrast enhancement as early as 1 to 2 days after initiation of therapy and commonly result in high radiologic response rates of 25% to 60%.[43-46] These apparent responses to antiangiogenic therapy may be partly a result of normalization of abnormally permeable tumor vessels and not always necessarily indicative of a true antiglioma effect (Fig 4). As a result, radiologic responses in studies with antiangiogenic agents should be interpreted with caution. There is a disappointing disparity between the unprecedented high response rates these agents produce in recurrent glioblastoma and the modest survival benefits, if any, that have been reported.[47] Although the duration of response or stability (PFS) or overall survival may be a more accurate indicator of a true anti-glioma effect, there is emerging data suggesting that the degree of initial response may also correlate with survival.[48] As with the Macdonald Criteria, the proposed criteria suggest that radiologic responses should persist for at least 4 weeks before they are considered as true responses.

Failure to Measure Nonenhancing Tumor

High-grade gliomas are infiltrative in nature, and their presence does not always result in disruption of the blood-brain barrier. In fact, determination of the extent of this nonenhancing component of the tumor, usually depicted on the MRI T2-weighted and fluid-attenuated inversion recovery (FLAIR) image sequences, can be difficult because peritumoral edema and delayed radiation white matter changes have similar radiographic appearances. Because the Macdonald Criteria do not account for the nonenhancing component of the tumor, this is especially problematic for low-grade gliomas (WHO grade 2) and anaplastic gliomas (WHO grade 3), where a significant portion of the tumor is typically nonenhancing.

As experience with antiangiogenic therapies has grown, especially with agents targeting VEGF and VEGF receptor, it has become apparent that a subset of patients who initially experience reduction in tumor contrast enhancement subsequently develop progressive increase in nonenhancing T2 or FLAIR signals suggestive of infiltrative tumor (Fig 5).[49-51] Increasing evidence suggests that anti-VEGF therapy may increase the tendency of tumor cells to co-opt existing blood vessels, resulting in an invasive nonenhancing phenotype.[52-54] Unlike the Macdonald Criteria, which do not take into account progressive nonenhancing disease, the new response assessment will consider enlarging areas of nonenhancing tumor as evidence of tumor progression (Tables 3 and 4). However, precise quantification of the increase in T2/FLAIR signal can be difficult and must be differentiated from other causes of increased T2 or FLAIR signal, such radiation effects, decreased corticosteroid dosing, demyelination, ischemic injury, infection, seizures, postoperative changes, or other treatment effects, before making a determination of progressive disease. Changes in T2/FLAIR signal that suggest infiltrating tumor include mass effect (as determined by sulcal effacement, ventricular compression, and thickening of the corpus callosum), infiltration of the cortical ribbon, and location outside of the radiation field. Although it would be preferable to have an objective measure of progressive nonenhancing recurrent disease similar to contrast-enhancing disease, the Response Assessment in Neuro-Oncology (RANO) Working Group felt that this was not possible at present given the limitations of current technology.

The initiation of these changes can be subtle, and convincing non–contrast-enhancing growth may require one or two confirmatory scans. If nonenhancing progression is determined after retrospective review of images, the scan at which these changes were first detected should serve as the progression scan.

Information downloaded from jco.ascopubs.org and provided by at DFG on April 1, 2014 from 193.174.53.85
Copyright © 2010 American Society of Clinical Oncology. All rights reserved.

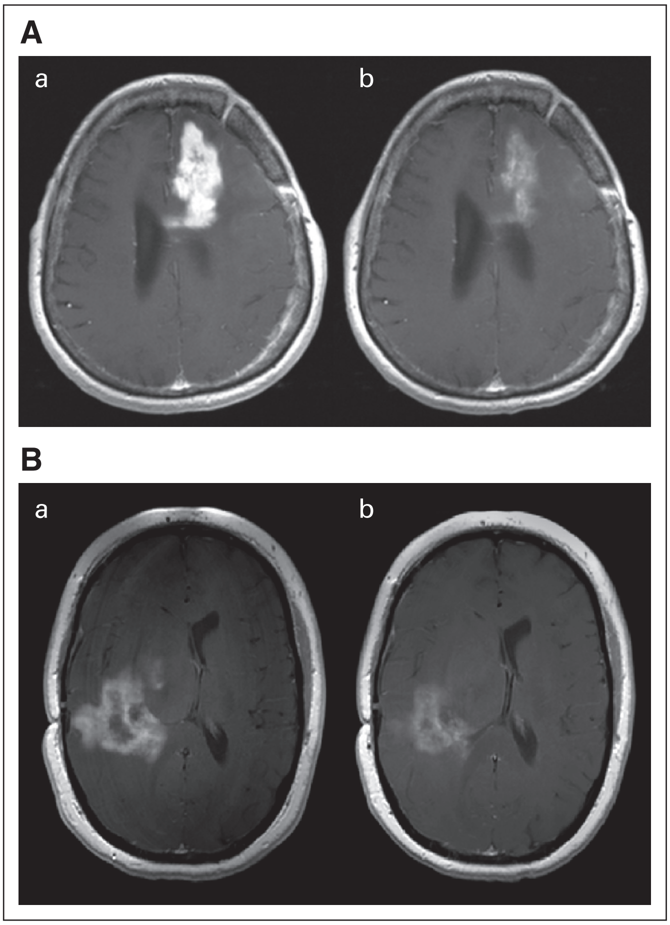

Fig 4. (A) Pseudoresponse. Axial T1-weighted contrast enhanced MRI of left frontal recurrent glioblastoma a) before and b) one day after therapy with cediranib (pan-VEGFR inhibitor) showing significant reduction in contrast enhancement. The reduction in contrast enhancement within 1 day of therapy is more likely to be caused by reduced vascular permeability to contrast than to a true antitumor effect. (Slide courtesy of A. Gregory Sorensen, Massachusetts General Hospital; Adapted with permission from Batchelor et al. Cancer Cell 11:83-95, 2007[43]). (B) Pseudoresponse. Axial T1-weighted contrast enhanced MRI of right parietal glioblastoma a) before and b) 1 day after therapy with XL184 (vascular endothelial growth factor receptor [VEGFR] and MET inhibitor) showing significant reduction in contrast enhancement. (Slide courtesy of A. Gregory Sorensen, Massachusetts General Hospital).

Progressive nonenhancing tumor is often associated with neurologic deterioration, and consequently, the clinical status of the patients may help in determining progressive disease. Given the lack of validated measures of neurologic function, a precise definition of neurologic deterioration is not included in the proposed response criteria. However, it is recommended that a decline in the Karnofsky performance score (KPS), Eastern Cooperative Oncology Group performance status, or WHO performance score be considered in determining clinical deterioration. The specific details are discussed later in the section defining progression.

PROCESS OF DEVELOPMENT OF THE UPDATED RESPONSE CRITERIA IN HIGH-GRADE GLIOMAS

Because of the limitations of the Macdonald Criteria, there has been an international effort in neuro-oncology to improve imaging response assessments for high-grade glioma and to enhance the interpretation of clinical trials involving novel agents that affect the blood-brain barrier such as antiangiogenic therapies. The RANO Working Group consists of neuro-oncologists, neurosurgeons, radiation oncologists, neuroradiologists, neuropsychologists, and experts in quality-of-life measures, in collaboration with government and industry. The RANO Working Group includes members with leadership roles in the major neuro-oncology organizations and brain tumor cooperative groups in both the United States and Europe. Recognizing the challenges in other neuro-oncologic clinical scenarios, imaging response recommendations are also being generated for low-grade glioma and the evaluation of surgically based therapies and will be reported separately.

In the following section, we outline a proposal for updated response criteria in high-grade gliomas from the RANO Working Group. It must be emphasized that this represents a work in progress. In coming years, as new volumetric and physiologic imaging techniques (eg, perfusion, permeability, and diffusion imaging; magnetic resonance spectroscopy; and metabolic imaging)[55,56] and other end points such as neuropsychological testing and quality-of-life measures are developed and validated in neuro-oncology, the RANO Working Group anticipates incorporating these parameters into the response criteria.

STANDARDIZATION OF IMAGING DEFINITIONS

Specific lesions must be evaluated serially, and comparative analysis of changes in the area of contrast enhancement, as well as the nonenhancing component, should be performed. As with the Macdonald Criteria, the product of the maximal cross-sectional enhancing diameters will be used to determine the size of the contrast-enhancing lesions.

Measureable and Nonmeasurable Disease for Contrast-Enhancing Lesions

Measurable disease is defined as bidimensionally contrast-enhancing lesions with clearly defined margins by CT or MRI scan, with two perpendicular diameters of at least 10 mm, visible on two or more axial slices that are preferably, at most, 5 mm apart with 0-mm skip. As with RECIST version 1.1, in the event the MRI is performed with thicker slices, the size of a measurable lesion at baseline should be two times the slice thickness.[10] In the event there are interslice gaps, this also needs to be considered in determining the size of measurable lesions at baseline. Measurement of tumor around a cyst or surgical cavity represents a particularly difficult challenge. In general, such lesions should be considered nonmeasurable unless there is a nodular component measuring $\geq$ 10 mm in diameter. The cystic or surgical cavity should not be measured in determining response.

Nonmeasurable disease is defined as either unidimensionally measurable lesions, masses with margins not clearly defined, or lesions with maximal perpendicular diameters less than 10 mm.

Patients without measurable disease, such as those who undergo a gross total resection, cannot respond and can only achieve stable disease as their best radiographic outcome. Therefore, if response rate is the primary end point of the study, patients with measurable disease are required for study eligibility. If duration of

© 2010 by American Society of Clinical Oncology

Information downloaded from jco.ascopubs.org and provided by at DFG on April 1, 2014 from 193.174.53.85
Copyright © 2010 American Society of Clinical Oncology. All rights reserved.

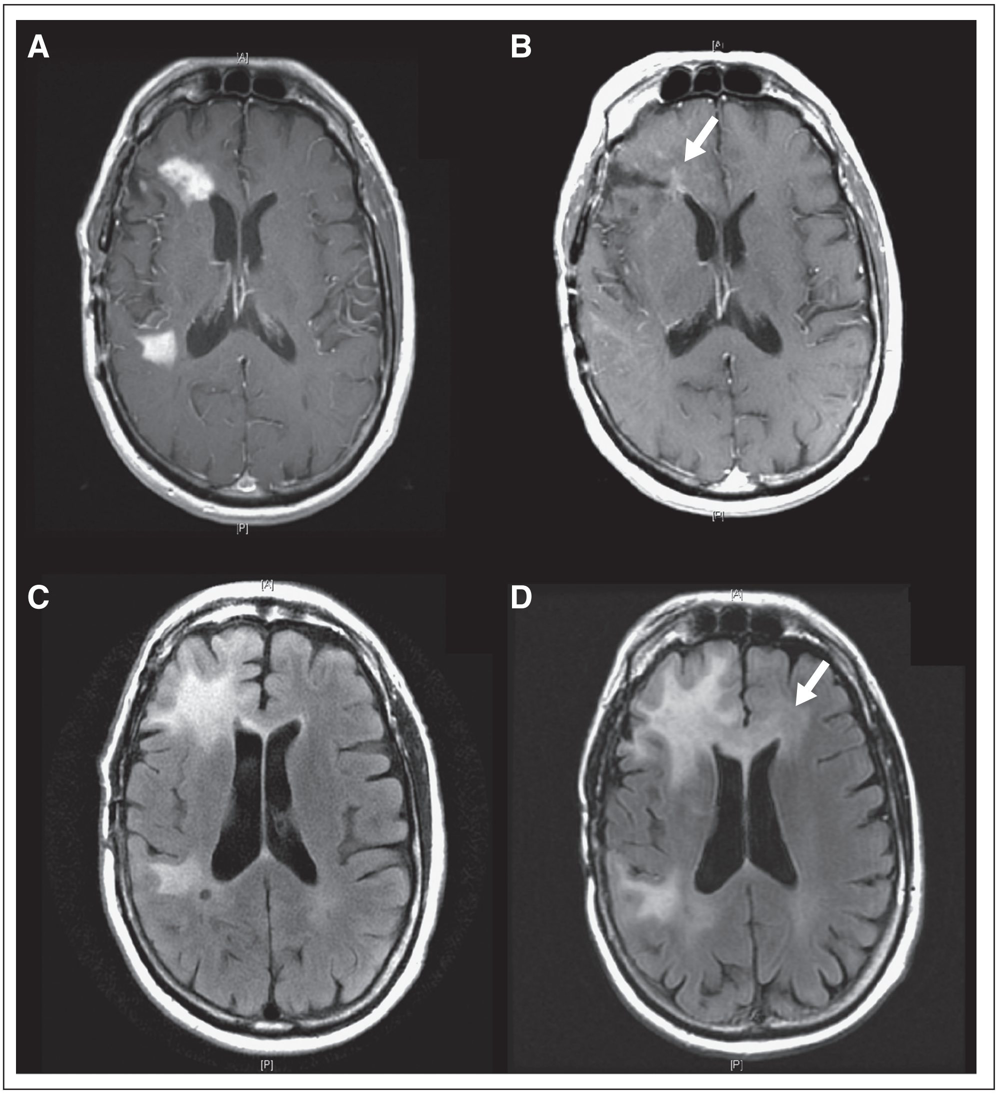

Fig 5. A 54-year-old patient with recurrent glioblastoma showing nonenhancing progression after bevacizumab therapy. Axial contrast-enhanced, T1-weighted images show (A) scan at recurrence showing multifocal right frontal glioblastoma; (B) decreased enhancement after 7 months of therapy that qualifies by Macdonald Criteria as partial response; (C) axial fluid-attenuated inversion recovery image at baseline and (D) after 7 months of therapy showing nonenhancing tumor progressing through corpus callosum to the left frontal lobe.

tumor control or survival is the primary end point, then patients with both measurable and nonmeasurable disease would be eligible for assessment because the determination of disease progression would be the primary interest.

Number of Lesions

If there are multiple contrast-enhancing lesions, a minimum of the two largest lesions should be measured, and the sum of the products of the perpendicular diameters of these lesions should be determined, similar to the criteria proposed for systemic tumors in RECIST version 1.1.[10] However, given the heterogeneity of high-grade gliomas and the difficulty in measuring some lesions, a maximum of five of the largest lesions may be measured. In general, the largest enlarging lesion(s) should be selected. However, emphasis should also be placed on lesions that allow reproducible repeated measurements. Occasionally, the largest lesions may not lend themselves to reproducible measurements, and the next largest lesions that can be measured reproducibly should be selected.

For patients with recurrent disease who have multiple lesions of which only one or two are increasing in size, the enlarging lesions should be considered the target lesions for evaluation of response. The other lesions will be considered nontarget lesions and should also be recorded. Rarely, unequivocal progression of a nontarget lesion requiring discontinuation of therapy or development of a new contrast-enhancing lesion may occur, even in the setting of stable disease or partial response in the target lesions. These changes would qualify as progression.

CRITERIA FOR DETERMINING FIRST PROGRESSION DEPENDING ON TIME FROM INITIAL CHEMORADIOTHERAPY

As mentioned earlier, 20% to 30% of patients develop pseudoprogression after chemoradiotherapy, especially within the first 3 months after completion of radiotherapy.[27] Given the difficulty of differentiating pseudoprogression from true progression in the first 12 weeks after irradiation, we propose excluding these patients from clinical trials for recurrent disease unless the progression is clearly outside the radiation field (eg, beyond the high-dose region or 80% isodose line)

Information downloaded from jco.ascopubs.org and provided by at DFG on April 1, 2014 from 193.174.53.85
Copyright © 2010 American Society of Clinical Oncology. All rights reserved.

Table 3. Criteria for Response Assessment Incorporating MRI and Clinical Factors

Response	Criteria
Complete response	Requires all of the following: complete disappearance of all enhancing measurable and nonmeasurable disease sustained for at least 4 weeks; no new lesions; stable or improved nonenhancing (T2/FLAIR) lesions; patients must be off corticosteroids (or on physiologic replacement doses only); and stable or improved clinically. Note: Patients with nonmeasurable disease only cannot have a complete response; the best response possible is stable disease.
Partial response	Requires all of the following: ≥ 50% decrease compared with baseline in the sum of products of perpendicular diameters of all measurable enhancing lesions sustained for at least 4 weeks; no progression of nonmeasurable disease; no new lesions; stable or improved nonenhancing (T2/FLAIR) lesions on same or lower dose of corticosteroids compared with baseline scan; the corticosteroid dose at the time of the scan evaluation should be no greater than the dose at time of baseline scan; and stable or improved clinically. Note: Patients with nonmeasurable disease only cannot have a partial response; the best response possible is stable disease.
Stable disease	Requires all of the following: does not qualify for complete response, partial response, or progression; stable nonenhancing (T2/FLAIR) lesions on same or lower dose of corticosteroids compared with baseline scan. In the event that the corticosteroid dose was increased for new symptoms and signs without confirmation of disease progression on neuroimaging, and subsequent follow-up imaging shows that this increase in corticosteroids was required because of disease progression, the last scan considered to show stable disease will be the scan obtained when the corticosteroid dose was equivalent to the baseline dose.
Progression	Defined by any of the following: ≥ 25% increase in sum of the products of perpendicular diameters of enhancing lesions compared with the smallest tumor measurement obtained either at baseline (if no decrease) or best response, on stable or increasing doses of corticosteroids*; significant increase in T2/FLAIR nonenhancing lesion on stable or increasing doses of corticosteroids compared with baseline scan or best response after initiation of therapy* not caused by comorbid events (eg, radiation therapy, demyelination, ischemic injury, infection, seizures, postoperative changes, or other treatment effects); any new lesion; clear clinical deterioration not attributable to other causes apart from the tumor (eg, seizures, medication adverse effects, complications of therapy, cerebrovascular events, infection, and so on) or changes in corticosteroid dose; failure to return for evaluation as a result of death or deteriorating condition; or clear progression of nonmeasurable disease.

NOTE. All measurable and nonmeasurable lesions must be assessed using the same techniques as at baseline.
Abbreviations: MRI, magnetic resonance imaging; FLAIR, fluid-attenuated inversion recovery.
*Stable doses of corticosteroids include patients not on corticosteroids.

or there is pathologic confirmation of disease progression. Table 2 lists these recommendations.

CRITERIA FOR ENTRY ONTO CLINICAL TRIALS FOR RECURRENT HIGH-GRADE GLIOMA

Currently, patients with any worsening of their imaging studies are eligible for entry onto clinical trials for recurrent gliomas, even if the change is minimal. We propose that patients should be required to have a 25% increase in the sum of the products of perpendicular diameters of the contrast-enhancing lesions, while on stable or increasing doses of corticosteroids, before they are considered to have progressive disease and are entered onto clinical trials for recurrent/progressive disease. Patients with new contrast-enhancing nonmeasurable disease may be considered for clinical trials in which PFS is the primary end point. Clinical deterioration or increase in corticosteroid dosing alone would not be sufficient to indicate progressive disease for entry onto clinical studies.

A particularly difficult problem involves patients receiving first-line antiangiogenic agents who develop predominantly nonenhancing disease at progression. This can be difficult to differentiate from treatment effects. If it seems clear that the nonenhancing changes represent tumor progression, these patients would also be eligible for enrollment onto clinical trials for recurrent disease, although their tumor will be considered nonmeasurable. As noted previously, although it would be preferable to have a more objective measure of progressive nonenhancing recurrent disease similar to contrast-enhancing disease, the RANO Working Group felt that this was not possible at present given the limitations of current technology.

DEFINITION OF RADIOGRAPHIC RESPONSE

Radiographic response should be determined in comparison to the tumor measurement obtained at pretreatment baseline for determination of response, and the smallest tumor measurement at either pretreatment baseline or after initiation of therapy should be used for determination of progression. Table 3 lists the criteria for radiographic changes after therapy. In the event that the radiographic changes are equivocal and it is unclear whether the patient is stable or has developed progressive disease, it is permissible to continue treatment and observe the patient closely, for example at 4-week intervals. If subsequent imaging studies demonstrate that progression has occurred, the date of progression should be the date of the scan at which this issue was first raised. The determination of radiographic response after treatment with agents, such as antiangiogenic therapies, that affect vascular permeability is particularly difficult. In these patients, consideration should be given to performing a second scan at 4 weeks to confirm the presence of response or stable disease.

All measurable and nonmeasurable lesions should be assessed using the same techniques as at baseline. Ideally, patients should be imaged on the same MRI scanner, or at least with the same magnet strength, for the duration of the study to reduce difficulties in interpreting changes.

Complete Response

Complete response requires all of the following: complete disappearance of all enhancing measurable and nonmeasurable disease sustained for at least 4 weeks; no new lesions; stable or improved nonenhancing (T2/FLAIR) lesions; and patient must be off corticosteroids or on physiologic replacement doses only, and stable or improved clinically. In the absence of a confirming scan 4 weeks later, this response will be considered only stable disease.

Partial Response

Partial response requires all of the following: ≥ 50% decrease, compared with baseline, in the sum of products of perpendicular

© 2010 by American Society of Clinical Oncology

Information downloaded from jco.ascopubs.org and provided by at DFG on April 1, 2014 from 193.174.53.85
Copyright © 2010 American Society of Clinical Oncology. All rights reserved.

Table 4. Summary of the Proposed RANO Response Criteria

Criterion	CR	PR	SD	PD
T1 gadolinium enhancing disease	None	≥ 50% ↓	< 50% ↓ but < 25% ↑	≥ 25% ↑*
T2/FLAIR	Stable or ↓	Stable or ↓	Stable or ↓	↑*
New lesion	None	None	None	Present*
Corticosteroids	None	Stable or ↓	Stable or ↓	NA†
Clinical status	Stable or ↑	Stable or ↑	Stable or ↑	↓*
Requirement for response	All	All	All	Any*

Abbreviations: RANO, Response Assessment in Neuro-Oncology; CR, complete response; PR, partial response; SD, stable disease; PD, progressive disease; FLAIR, fluid-attenuated inversion recovery; NA, not applicable.
*Progression occurs when this criterion is present.
†Increase in corticosteroids alone will not be taken into account in determining progression in the absence of persistent clinical deterioration.

diameters of all measurable enhancing lesions sustained for at least 4 weeks; no progression of nonmeasurable disease; no new lesions; stable or improved nonenhancing (T2/FLAIR) lesions on same or lower dose of corticosteroids compared with baseline scan; and patient must be on a corticosteroid dose not greater than the dose at time of baseline scan and is stable or improved clinically. In the absence of a confirming scan 4 weeks later, this response will be considered only stable disease.

Stable Disease

Stable disease occurs if the patient does not qualify for complete response, partial response, or progression (see next section) and requires the following: stable nonenhancing (T2/FLAIR) lesions on same or lower dose of corticosteroids compared with baseline scan and clinically stable status. In the event that the corticosteroid dose was increased for new symptoms and signs without confirmation of disease progression on neuroimaging, and subsequent follow-up imaging shows that this increase in corticosteroids was required because of disease progression, the last scan considered to show stable disease will be the scan obtained when the corticosteroid dose was equivalent to the baseline dose.

Progression

Progression is defined by any of the following: ≥ 25% increase in sum of the products of perpendicular diameters of enhancing lesions (compared with baseline if no decrease) on stable or increasing doses of corticosteroids; a significant increase in T2/FLAIR nonenhancing lesions on stable or increasing doses of corticosteroids compared with baseline scan or best response after initiation of therapy, not due to comorbid events; the appearance of any new lesions; clear progression of nonmeasurable lesions; or definite clinical deterioration not attributable to other causes apart from the tumor, or to decrease in corticosteroid dose. Failure to return for evaluation as a result of death or deteriorating condition should also be considered as progression.

Increase in corticosteroid dose alone, in the absence of clinical deterioration related to tumor, will not be used as a determinant of progression. Patients with stable imaging studies whose corticosteroid dose was increased for reasons other than clinical deterioration related to tumor do not qualify for stable disease or progression. They should be observed closely. If their corticosteroid dose can be reduced back to baseline, they will be considered as having stable disease; if further clinical deterioration related to tumor becomes apparent, they will be considered to have progression. The date of progression should be the first time point at which corticosteroid increase was necessary.

The definition of clinical deterioration is left to the discretion of the treating physician, but it is recommended that a decline in the KPS from 100 or 90 to 70 or less, a decline in KPS of at least 20 from 80 or less, or a decline in KPS from any baseline to 50 or less, for at least 7 days, be considered neurologic deterioration unless attributable to comorbid events or changes in corticosteroid dose. Similarly, a decline in the Eastern Cooperative Oncology Group and WHO performance scores from 0 or 1 to 2 or 2 to 3 would be considered neurologic deterioration.

Patients with nonmeasurable enhancing disease whose lesions have significantly increased in size and become measurable (minimal bidirectional diameter of ≥ 10 mm and visible on at least two axial slices that are preferably, at most, 5 mm apart with 0-mm skip) will also be considered to have experienced progression. The transition from a nonmeasurable lesion to a measurable lesion resulting in progression can theoretically occur with relatively small increases in tumor size (eg, a 9 × 9 mm lesion [nonmeasurable] increasing to a 10 × 11 mm lesion [measurable]). Ideally, the change should be significant (> 5 mm increase in maximal diameter or ≥ 25% increase in sum of the products of perpendicular diameters of enhancing lesions). In general, if there is doubt about whether the lesion has progressed, continued treatment and close follow-up evaluation will help clarify whether there is true progression.

If there is uncertainty regarding whether there is progression, the patient may continue on treatment and remain under close observation (eg, evaluated at 4-week intervals). If subsequent evaluations suggest that the patient is in fact experiencing progression, then the date of progression should be the time point at which this issue was first raised.

MULTIFOCAL TUMORS

For multifocal lesions, progressive disease is defined as ≥ 25% increase in the sum of products of perpendicular diameters of all measurable lesions compared with the smallest tumor measurements after initiation of therapy (Table 3). The appearance of a new lesion or unequivocal progression of nontarget lesions will also be considered progression. Partial response is defined as ≥ 50% decrease, compared with baseline, in the sum of products of perpendicular diameters of all measurable lesions sustained for at least 4 weeks with stable or decreasing corticosteroid doses.

 © 2010 by American Society of Clinical Oncology

Information downloaded from jco.ascopubs.org and provided by at DFG on April 1, 2014 from 193.174.53.85
Copyright © 2010 American Society of Clinical Oncology. All rights reserved.

ROLE OF VOLUMETRIC AND ADVANCED MRI ASSESSMENT

Given the limitations of two-dimensional tumor measurements, there is significant interest in volumetric anatomic assessment. The use of volumetric assessment would allow more accurate determination of the contrast-enhancing and nonenhancing volumes and overcome the limitations of two-dimensional measurements of lesions surrounding a surgical cavity.[14-16] However, the RANO Working Group and colleagues in neuroradiology do not believe that there is sufficient standardization and availability to recommend adoption of volumetric assessment of tumor volume at present. Nonetheless, this is an important area of research. Eventually, as volumetric imaging becomes more standardized and widely available and as data validating this approach emerge, it may be possible to incorporate volumetric measurements in the response assessment of high-grade gliomas.

Emerging data also suggest that advanced MRI techniques such as perfusion imaging (dynamic susceptibility MRI), permeability imaging (dynamic contrast-enhanced MRI), diffusion imaging, magnetic resonance spectroscopy, and [^{18}F]-fluorothymidine and amino acid positron emission tomography may predict tumor response or allow the differentiation of nonenhancing tumor from other causes of increased FLAIR signal. These techniques will require rigorous clinical validation studies before they can be incorporated into response criteria used in clinical trials in high-grade gliomas.

OTHER METHODS OF DETERMINING EFFICACY

Growing data suggest that other end points such as neurocognitive function, quality of life, and corticosteroid use may be used to measure clinical benefit. At present, these end points are not sufficiently validated to be incorporated into the current response criteria but could be added in the future as further data emerge.

CONCLUSION

We propose updated response assessments for the evaluation of therapies in high-grade gliomas incorporating MRI characteristics to address the recognized and accepted limitations of the current Macdonald Criteria. These recommendations were generated as part of an international neuro-oncology effort with consensus building and are an attempt to develop standardized assessment criteria. Implementation into future clinical trials will be critical so we can validate the criteria as a surrogate to end points such as survival and, ultimately, improve the accuracy and efficiency of the early evaluation of novel therapies.

AUTHORS' DISCLOSURES OF POTENTIAL CONFLICTS OF INTEREST

Although all authors completed the disclosure declaration, the following author(s) indicated a financial or other interest that is relevant to the subject matter under consideration in this article. Certain relationships marked with a "U" are those for which no compensation was received; those relationships marked with a "C" were compensated. For a detailed description of the disclosure categories, or for more information about ASCO's conflict of interest policy, please refer to the Author Disclosure Declaration and the Disclosures of Potential Conflicts of Interest section in Information for Contributors.

Employment or Leadership Position: None **Consultant or Advisory Role:** Michael A. Vogelbaum, AstraZeneca (C), Schering-Plough (C) **Stock Ownership:** None **Honoraria:** Michael A. Vogelbaum, BrainLAB, BrainLAB, Genentech, Eisai **Research Funding:** None **Expert Testimony:** None **Other Remuneration:** None

AUTHOR CONTRIBUTIONS

Conception and design: Patrick Y. Wen, David R. Macdonald, David A. Reardon, Timothy F. Cloughesy, A. Gregory Sorensen, Evanthia Galanis, John DeGroot, Wolfgang Wick, Mark R. Gilbert, Andrew B. Lassman, Christina Tsien, Tom Mikkelsen, Eric T. Wong, Marc C. Chamberlain, Roger Stupp, Kathleen R. Lamborn, Michael A. Vogelbaum, Martin J. van den Bent, Susan M. Chang
Administrative support: Patrick Y. Wen
Provision of study materials or patients: Patrick Y. Wen, Susan M. Chang, A. Gregory Sorensen
Collection and assembly of data: Patrick Y. Wen, David R. Macdonald, David A. Reardon, Timothy F. Cloughesy, A. Gregory Sorensen, Evanthia Galanis, John DeGroot, Wolfgang Wick, Mark R. Gilbert, Andrew B. Lassman, Christina Tsien, Tom Mikkelsen, Eric T. Wong, Marc C. Chamberlain, Roger Stupp, Kathleen R. Lamborn, Michael A. Vogelbaum, Martin J. van den Bent, Susan M. Chang
Data analysis and interpretation: Patrick Y. Wen, David R. Macdonald, David A. Reardon, Timothy F. Cloughesy, A. Gregory Sorensen, Evanthia Galanis, John DeGroot, Wolfgang Wick, Mark R. Gilbert, Andrew B. Lassman, Christina Tsien, Tom Mikkelsen, Eric T. Wong, Marc C. Chamberlain, Roger Stupp, Kathleen R. Lamborn, Michael A. Vogelbaum, Martin J. van den Bent, Susan M. Chang
Manuscript writing: Patrick Y. Wen, David R. Macdonald, David A. Reardon, Timothy F. Cloughesy, A. Gregory Sorensen, Evanthia Galanis, John DeGroot, Wolfgang Wick, Mark R. Gilbert, Andrew B. Lassman, Christina Tsien, Tom Mikkelsen, Eric T. Wong, Roger Stupp, Kathleen R. Lamborn, Michael A. Vogelbaum, Martin J. van den Bent, Susan M. Chang
Final approval of manuscript: Patrick Y. Wen, David R. Macdonald, David A. Reardon, Timothy F. Cloughesy, A. Gregory Sorensen, Evanthia Galanis, John DeGroot, Wolfgang Wick, Mark R. Gilbert, Andrew B. Lassman, Christina Tsien, Tom Mikkelsen, Eric T. Wong, Marc C. Chamberlain, Roger Stupp, Kathleen R. Lamborn, Michael A. Vogelbaum, Martin J. van den Bent, Susan M. Chang

REFERENCES

1. Central Brain Tumor Registry of the United States: Statistical report: Primary brain tumors in the United States, 2000-2004. http://www.cbtrus.org/reports/2007-2008/2007report.pdf

2. Wen PY, Kesari S: Malignant gliomas in adults. N Engl J Med 359:492-507, 2008

3. Wong ET, Hess KR, Gleason MJ, et al: Outcomes and prognostic factors in recurrent glioma patients enrolled onto phase II clinical trials. J Clin Oncol 17:2572-2578, 1999

4. Lamborn KR, Yung WK, Chang SM, et al: Progression-free survival: An important end point in evaluating therapy for recurrent high-grade gliomas. Neuro Oncol 10:162-170, 2008

5. Macdonald D, Cascino T, Schold SJ, et al: Response criteria for phase II studies of supratentorial malignant glioma. J Clin Oncol 8:1277-1280, 1990

6. WHO: WHO Handbook for Reporting Results of Cancer Treatment. World Health Organization Offset Publication No. 48. Geneva, Switzerland, WHO, 1979

7. Miller A, Hoogstraten B, Staquet M, et al: Reporting results of cancer treatment. Cancer 47: 207-214, 1981

8. Rees J: Advances in magnetic resonance imaging of brain tumours. Curr Opin Neurol 16:643-650, 2003

9. Therasse P, Arbuck S, Eisenhauer E, et al: New guidelines to evaluate the response to treatment in solid tumors: European Organization for

© 2010 by American Society of Clinical Oncology

Information downloaded from jco.ascopubs.org and provided by at DFG on April 1, 2014 from 193.174.53.85
Copyright © 2010 American Society of Clinical Oncology. All rights reserved.

Research and Treatment of Cancer, National Cancer Institute of the United States, National Cancer Institute of Canada. J Natl Cancer Inst 92:205-216, 2000

10. Eisenhauer EA, Therasse P, Bogaerts J, et al: New Response Evaluation Criteria in Solid Tumours: Revised RECIST guideline (version 1.1). Eur J Cancer 45:228-247, 2009

11. Warren KE, Patronas N, Aikin AA, et al: Comparison of one-, two-, and three-dimensional measurements of childhood brain tumors. J Natl Cancer Inst 93:1401-1405, 2001

12. Shah GD, Kesari S, Xu R, et al: Comparison of linear and volumetric criteria in assessing tumor response in adult high-grade gliomas. Neuro Oncol 8:38-46, 2006

13. Galanis E, Buckner JC, Maurer MJ, et al: Validation of neuroradiologic response assessment in gliomas: Measurement by RECIST, two-dimensional, computer-assisted tumor area, and computer-assisted tumor volume methods. Neuro Oncol 8:156-165, 2006

14. Sorensen A, Patel S, Harmath C, et al: Comparison of diameter and perimeter methods for tumor volume calculation. J Clin Oncol 19:551-557, 2001

15. Sorensen AG, Batchelor TT, Wen PY, et al: Response criteria for glioma. Nat Clin Pract Oncol 5:634-644, 2008

16. Henson JW, Ulmer S, Harris GJ: Brain tumor imaging in clinical trials. Am J Neuroradiol 29:419-424, 2008

17. van den Bent MJ, Vogelbaum MA, Wen PY, et al: End point assessment in gliomas: Novel treatments limit usefulness of classical Macdonald's Criteria. J Clin Oncol 27:2905-2908, 2009

18. Cairncross JG, Macdonald DR, Pexman JH, et al: Steroid-induced CT changes in patients with recurrent malignant glioma. Neurology 38:724-726, 1988

19. Watling CJ, Lee DH, Macdonald DR, et al: Corticosteroid-induced magnetic resonance imaging changes in patients with recurrent malignant glioma. J Clin Oncol 12:1886-1889, 1994

20. Henegar MM, Moran CJ, Silbergeld DL: Early postoperative magnetic resonance imaging following nonneoplastic cortical resection. J Neurosurg 84:174-179, 1996

21. Kumar AJ, Leeds NE, Fuller GN, et al: Malignant gliomas: MR imaging spectrum of radiation therapy- and chemotherapy-induced necrosis of the brain after treatment. Radiology 217:377-384, 2000

22. Ulmer S, Braga TA, Barker FG 2nd, et al: Clinical and radiographic features of peritumoral infarction following resection of glioblastoma. Neurology 67:1668-1670, 2006

23. Finn MA, Blumenthal DT, Salzman KL, et al: Transient postictal MRI changes in patients with brain tumors may mimic disease progression. Surg Neurol 67:246-250, 2007

24. Stupp R, Mason WP, van den Bent MJ, et al: Radiotherapy plus concomitant and adjuvant temozolomide for glioblastoma. N Engl J Med 352:987-996, 2005

25. Brem SS, Bierman PJ, Black P, et al: Central nervous system cancers. J Natl Compr Canc Netw 6:456-504, 2008

26. de Wit MC, de Bruin HG, Eijkenboom W, et al: Immediate post-radiotherapy changes in malignant glioma can mimic tumor progression. Neurology 63:535-537, 2004

27. Brandsma D, Stalpers L, Taal W, et al: Clinical features, mechanisms, and management of pseudoprogression in malignant gliomas. Lancet Oncol 9:453-461, 2008

28. Chamberlain MC, Glantz MJ, Chalmers L, et al: Early necrosis following concurrent Temodar and radiotherapy in patients with glioblastoma. J Neurooncol 82:81-83, 2007

29. Taal W, Brandsma D, de Bruin HG, et al: Incidence of early pseudo-progression in a cohort of malignant glioma patients treated with chemoirradiation with temozolomide. Cancer 113:405-410, 2008

30. Brandes AA, Franceschi E, Tosoni A, et al: MGMT promoter methylation status can predict the incidence and outcome of pseudoprogression after concomitant radiochemotherapy in newly diagnosed glioblastoma patients. J Clin Oncol 26:2192-2197, 2008

31. Cairncross JG, Pexman JH, Rathbone MP, et al: Postoperative contrast enhancement in patients with brain tumor. Ann Neurol 17:570-572, 1985

32. Sato N, Bronen RA, Sze G, et al: Postoperative changes in the brain: MR imaging findings in patients without neoplasms. Radiology 204:839-846, 1997

33. Cairncross JG, Pexman JH, Rathbone MP: Post-surgical contrast enhancement mimicking residual brain tumour. Can J Neurol Sci 12:75, 1985

34. Smith JS, Cha S, Mayo MC, et al: Serial diffusion-weighted magnetic resonance imaging in cases of glioma: Distinguishing tumor recurrence from postresection injury. J Neurosurg 103:428-438, 2005

35. Matheus MG, Castillo M, Ewend M, et al: CT and MR imaging after placement of the GliaSite radiation therapy system to treat brain tumor: Initial experience. Am J Neuroradiol 25:1211-1217, 2004

36. Parney IF, Kunwar S, McDermott M, et al: Neuroradiographic changes following convection-enhanced delivery of the recombinant cytotoxin interleukin 13-PE38QQR for recurrent malignant glioma. J Neurosurg 102:267-275, 2005

37. Floeth FW, Aulich A, Langen KJ, et al: MR imaging and single-photon emission CT findings after gene therapy for human glioblastoma. Am J Neuroradiol 22:1517-1527, 2001

38. Ross DA, Sandler HM, Balter JM, et al: Imaging changes after stereotactic radiosurgery of primary and secondary malignant brain tumors. J Neurooncol 56:175-181, 2002

39. Young GS: Advanced MRI of adult brain tumors. Neurol Clin 25:947-973, 2007

40. Chen W: Clinical applications of PET in brain tumors. J Nucl Med 48:1468-1481, 2007

41. Soares DP, Law M: Magnetic resonance spectroscopy of the brain: Review of metabolites and clinical applications. Clin Radiol 64:12-21, 2009

42. Sibtain NA, Howe FA, Saunders DE: The clinical value of proton magnetic resonance spectroscopy in adult brain tumours. Clin Radiol 62:109-119, 2007

43. Batchelor T, Sorensen A, di Tomaso E, et al: AZD2171, a pan-VEGF receptor tyrosine kinase inhibitor, normalizes tumor vasculature and alleviates edema in glioblastoma patients. Cancer Cell 11:83-95, 2007

44. Vredenburgh JJ, Desjardins A, Herndon JE 2nd, et al: Bevacizumab plus irinotecan in recurrent glioblastoma multiforme. J Clin Oncol 25:4722-4729, 2007

45. Friedman HS, Prados MD, Wen PY, et al: Bevacizumab alone and in combination with irinotecan in recurrent glioblastoma. J Clin Oncol 27:4733-4740, 2009

46. Kreisl TN, Kim L, Moore K, et al: Phase II trial of single-agent bevacizumab followed by bevacizumab plus irinotecan at tumor progression in recurrent glioblastoma. J Clin Oncol 27:740-745, 2009

47. Norden AD, Drappatz J, Muzikansky A, et al: An exploratory survival analysis of anti-angiogenic therapy for recurrent malignant glioma. J Neurooncol 92:149-155, 2009

48. Sorensen AG, Batchelor TT, Zhang WT, et al: A "vascular normalization index" as potential mechanistic biomarker to predict survival after a single dose of cediranib in recurrent glioblastoma patients. Cancer Res 69:5296-5300, 2009

49. Norden AD, Young GS, Setayesh K, et al: Bevacizumab for recurrent malignant gliomas: Efficacy, toxicity, and patterns of recurrence. Neurology 70:779-787, 2008

50. Narayana A, Raza S, Golfinos JG, et al: Bevacizumab therapy in recurrent high grade glioma: Impact on local control and survival. J Clin Oncol 26:691s, 2008 (suppl; abstr 13000)

51. Norden AD, Drappatz J, Wen PY: Novel anti-angiogenic therapies for malignant gliomas. Lancet Neurol 7:1152-1160, 2008

52. Rubenstein J, Kim J, Ozawa T, et al: Anti-VEGF antibody treatment of glioblastoma prolongs survival but results in increased vascular cooption. Neoplasia 2:306-314, 2000

53. Paez-Ribes M, Allen E, Hudock J, et al: Anti-angiogenic therapy elicits malignant progression of tumors to increased local invasion and distant metastasis. Cancer Cell 15:220-231, 2009

54. Bergers G, Hanahan D: Modes of resistance to anti-angiogenic therapy. Nat Rev Cancer 8:592-603, 2008

55. Chawla S, Poptani H, Melhem ER: Anatomic, physiologic and metabolic imaging in neuro-oncology. Cancer Treat Res 143:3-42, 2008

56. Ullrich RT, Kracht LW, Jacobs AH: Neuroimaging in patients with gliomas. Semin Neurol 28:484-494, 2008

Information downloaded from jco.ascopubs.org and provided by at DFG on April 1, 2014 from 193.174.53.85
Copyright © 2010 American Society of Clinical Oncology. All rights reserved.
